# A Chemically Induced CRISPR/dCas13^FCPF^ Platform for Precise and Programmable RNA Regulation

**DOI:** 10.1021/acs.jmedchem.5c01609

**Published:** 2025-10-22

**Authors:** Sebastian Hasselbeck, Jianhui Wang, Zhaodai Bai, Tobias Hüfner, Gerhard Hummer, Phillip Grote, Xinlai Cheng

**Affiliations:** † Buchmann Institute for Molecular Life Sciences, 686742 Goethe University Frankfurt am Main, 60438 Frankfurt am Main, Germany; ‡ Institute of Pharmaceutical Chemistry, Goethe University Frankfurt am Main, 60438 Frankfurt am Main, Germany; § Frankfurt Cancer Institute, 40063 Goethe University Frankfurt am Main, 60596 Frankfurt am Main, Germany; ∥ Institute for Tumor Biology and Experimental Therapy, Georg-Speyer-Haus, 60596 Frankfurt am Main, Germany; ⊥ 28273 Max-Planck-Institute of Biophysics, 60438 Frankfurt am Main, Germany; # University Cancer Center (UCT) Frankfurt, 60590 Frankfurt am Main, Germany; ∇ Mildred-Scheel-Nachwuchszentrum (MSNZ) Frankfurt, 60590 Frankfurt am Main Germany

## Abstract

Alternative splicing enhances proteomic diversity, yet its dysregulation drives cancer, neurodegeneration, and inherited disease. Small-molecule splicing modulators, while clinically validated, like risdiplam, often lack locus specificity, producing off-target effects. CRISPR/Cas13 enables programmable transcript-level targeting, but dCas13 fusion effectors are bulky and can hamper delivery and RNA homeostasis. Building on our previous Chem-CRISPR/dCas9^FCPF^ system for epigenome editing, we now introduce Chem-CRISPR/dCas13^FCPF^, a modular platform that covalently tethers a perfluorobiphenyl-tagged small molecule to dCas13 via a four-residue FCPF π-clamp tag. Guided by crRNAs, dRfxCas13d^FCPF^ recruits a risdiplam-derived conjugate to the *SMN2* exon 7 splice region, inducing exon inclusion at ligand doses ∼500-fold lower than those of free risdiplam and with no detectable effects at known risdiplam-sensitive transcripts in our assays. The approach generalizes to additional transcripts by crRNA redesign. By coupling CRISPR addressability with dose-sparing chemical action, Chem-CRISPR/dCas13^FCPF^ establishes a proximity-induced, chemically controllable route to precise RNA modulation suitable for therapeutic exploration.

## Graphical abstract

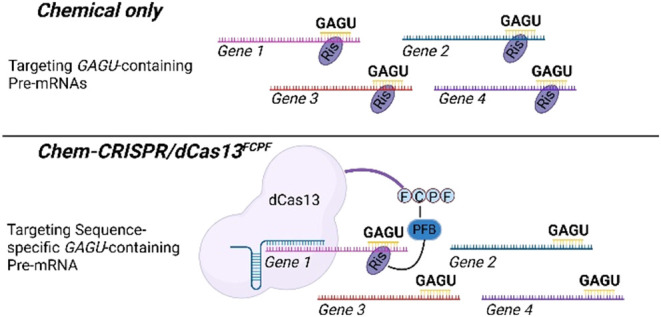

## Introduction

Alternative splicing generates multiple mRNA isoforms from a single primary transcript by excising introns and selecting alternative exons.^1,2^ Through regulated spliceosome assembly and exon choice—including occasional intron retention—this process expands proteomic diversity primarily in eukaryotes.^1–3^ Despite its importance, splicing is error-prone, and aberrant events are implicated in numerous genetic disorders.^3,4^

A prominent example is spinal muscular atrophy (SMA), a neuromuscular disease caused by insufficient expression of survival motor neuron (SMN) protein, which is crucial for RNA metabolism and motor neuron function.^4–6^ In SMA, loss-of-function mutations or deletions in *SMN1* reduce full-length SMN protein expression.^5,7^ The nearly identical paralog, *SMN2*, partially compensates; however, a C-to-T transition at the 3′ end of exon 7 disrupts splice site (ss) recognition, leading to exon 7 skipping in ∼ 80–90% of *SMN2* transcripts (*SMN2_*Δ*7*) and a truncated, nonfunctional protein^5,7^

Several FDA-approved SMA therapies address this deficiency. Onasemnogene abeparvovec delivers a functional *SMN1* gene via adeno-associated virus, but remains costly.^8,9^ Nusinersen (Spinraza), an antisense oligonucleotide, promotes inclusion of exon 7 by modulating spliceosome assembly.^8,10^ Risdiplam (1), a small-molecule splicing modifier, similarly enhances exon 7 inclusion by binding *SMN2* pre-mRNA and stabilizing spliceosomal engagement.^4,11^ Although nusinersen retains a significant clinical presence due to its early approval, risdiplam is gaining traction owing to its once-daily oral administration and at-home convenience.^12,13^ Risdiplam is designed to enhance uridine-rich subunit small nuclear ribonucleoprotein 1 (U1 snRNP)^14^ recruitment at the *SMN2* exon 7 splice site (Figure 1A,B).^4^ However, it also modulates GA/GU 5′ splice-sites (5′ss) contexts in other transcripts, such as *FOXM1*, *APLP2*, *STRN3*, and *MADD*, resulting in dose-limiting side effects, including fever, diarrhea, and upper respiratory tract infection.^15,16^

**Figure 1:**
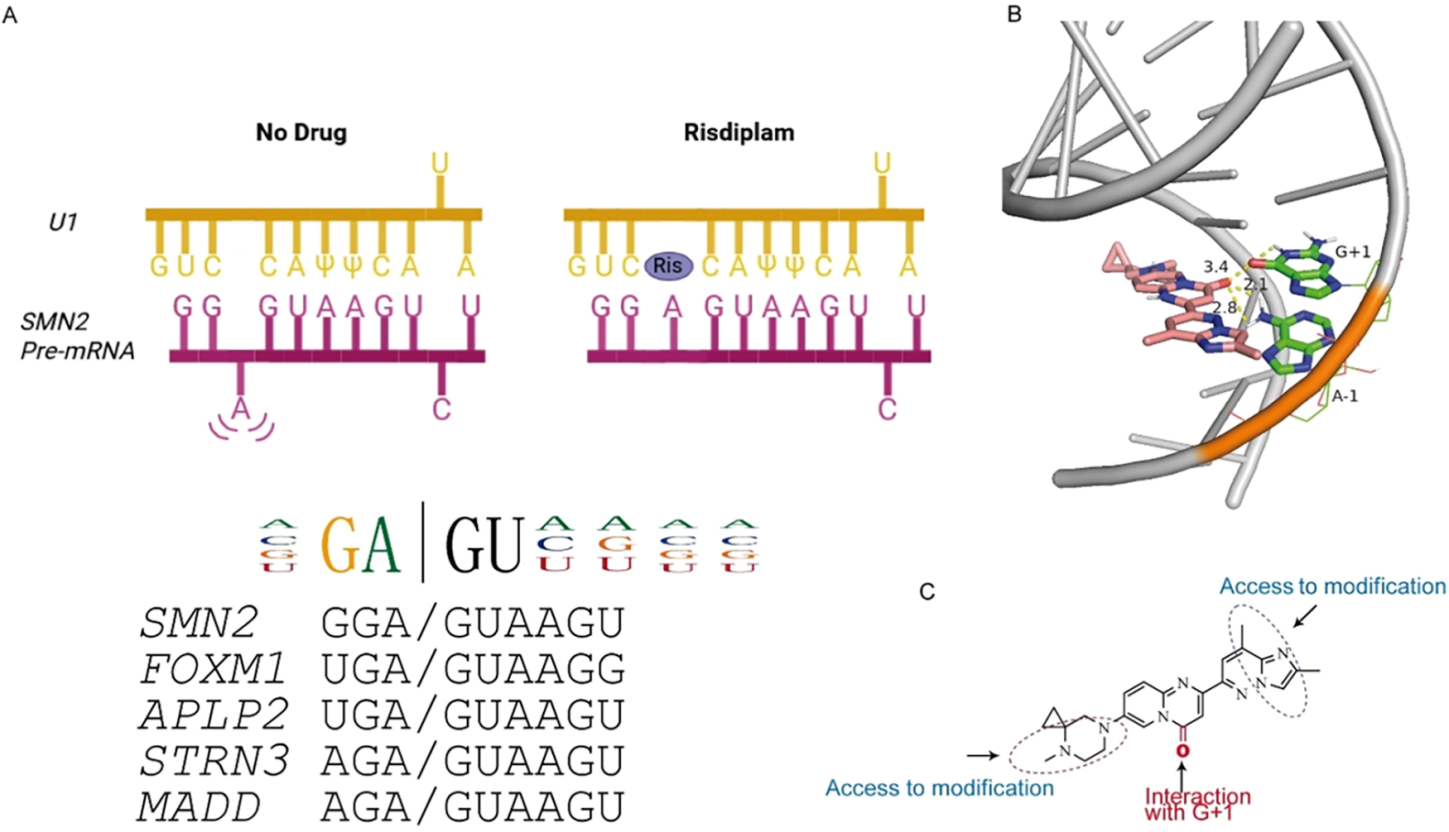
Designing the exit vector and synthesis. (A) Risdiplam binding at the *SMN2* exon 7 splice site. In the absence of a drug, a bulged adenosine impedes U1 snRNP assembly on exon 7. Risdiplam restores this interaction, stabilizing the *SMN2* pre-mRNA–U1 complex. The risdiplam IUPAC motif is shown at the left, alongside motifs from key off-targets (*FOXM1, APLP2, STRN3, MADD*). Created by BioRender (B) NMR structure of *SMN2* pre-mRNA (gray) in complex with risdiplam (pink; PDB entry 8R62). The G+1 and A-1 nucleotides, which coordinate the keto group of risdiplam, are highlighted in green. (C) Chemical structure of risdiplam **1**. The keto group (red) engages in A-1/G+1, while the peripheral regions serve as exit vectors for derivatization.

CRISPR–Cas13 systems (class 2, type VI), including Cas13a–d, X, and Y, offer a programmable RNA-targeting platform for transcriptome engineering in mammalian systems.^17–26^ Cas13 recognizes single-stranded RNA via a CRISPR RNA (crRNA) consisting of a direct repeat (DR) and a 22–30-nt spacer.^18,22,23,27^ Catalytically inactive Cas13 (dCas13) retains RNA-binding activity and has been fused to effector domains for targeted splicing modulation,^28^ RNA editing,^17^ m^6^A modification,^29^ and live-cell RNA imaging.^30^ However, the large size and complexity of such fusions hinder delivery and may perturb endogenous RNA homeostasis, limiting translational potential.^17,28–31^

To address these challenges, we previously developed Chem-CRISPR/dCas9^FCPF^ system, in which dCas9 carries a four-amino-acid tag (FCPF; Phe–Cys–Pro–Phe) that undergoes selective conjugation with perfluorobiphenyl (PFB **2**) via a π-clamp mechanism.^32–34^ The π-clamp relies on phenylalanine-mediated positioning of PFB for selective aromatic substitution at the cysteine sulfur atom.^34,35^ This platform enabled chemically induced, locus-specific epigenome editing in mammalian cells.^36,37^ Here, we adapt the strategy to a CRISPR/dCas13 framework using risdiplam-derived PFB conjugates (Ris-PFBs) to achieve site-specific RNA modulation at substantially lower ligand concentrations. This work establishes a modular and chemically controllable dCas13 platform for programmable RNA editing.

## Chemistry

### Designing the Exit Vector

Risdiplam represents the first FDA-approved small molecule for SMA, acting by restoring *SMN2* exon 7 inclusion by stabilizing a critical bulged adenosine within the RNA duplex formed between GA/GU-containing *SMN2* exon 7 and U1 snRNA, thereby facilitating spliceosome assembly at the 5′ss of intron 7 (Figure 1A,B).^4,38–40^ Despite clinical success, transcriptome-wide analyses in SMA patient-derived biroblasts treated with risdiplam (1 μM) revealed extensive off-target modulating, affecting over 10,000 genes and more than 2,000 unintended splicing events,^15,41^ including *FOXM1*, *APLP2*, *STRN3*, and *MADD*, which harbor GA/GU motifs at exon 5′ss (Figure 1A).^15,41^

Previously, we demonstrated that the Chem-CRISPR/dCas9^FCPF^ platform channel ardently enhances the specificity of chemically induced epigenetic modulation at the single-gene level.^32^ To extend this precision-targeting strategy to RNA modulation using the CRISPR/dCas13 system, we conducted structural analyses guided by recent NMR data for the risdiplam-RNA complex (PDB: 8R62 and Figure 1B).^42^ We noted that the keto group of risdiplam (Figure 1B, in blue and pink; Figure 1C, keto group in red) engages the A-1/G+1 nucleotides (Figure 1B, in green and blue) within the *SMN2* pre-mRNA/U1 duplex (Figure 1B, in gray), an interaction critical for productive intercalation.^42^ Additional features primarily shape pharmacology; for instance, the cyclopropyl on the piperazine lowers basicity, mitigating volume of distribution and phospholipidosis risk.^4^ On this basis, we selected peripheral linker exit vectors that project away from the binding pharmacophore and RNA-contacting elements to permit chemical modification.^43^ Positions distal to the keto and A-1/G+1 interaction were chosen to incorporate PFB moiety for covalent capture by dCas13^FCPF^, establishing a chemical inducible CRISPR/dCas13 platform (Figure 1C).^32,34–36,42^

### Chemical Synthesis

The initial attempt to introduce the exit vector involved modifications of the imidazo­[1,2-*b*]­pyridazine moiety of risdiplam. The synthetic route, adapted from Ratni et al.,^4^ began with the preparation of 8-bromo-6-chloro-2-methyl-imidazo­[1,2-*b*]­pyridazine **5**, synthesized by reacting 3-amino-4-bromo-6-chloropyradizine **3** with 1-brom-2,2-dimethoxypropane **4** in isopropanol in the presence of catalytic pyridinium *p*-toluenesulfonate (PPTS). Subsequent nucleophilic aromatic substitution with cysteamine hydrochloride **6** in acetonitrile (ACN) and diisopropylethylamine (DIPEA) afforded intermediate 2-[(6-chloro-2-methyl-imidazo­[1,2-*b*]­pyridazine-8-yl)­amino]­ethanethiole **7**, which was subsequently protected with a *tert*-butyloxycarbonyl (Boc) group to yield compound **8**.

Compound **8** was intended for coupling via a Suzuki cross-reaction with 2-chloro-7-fluoro-4*H*-pyrido­[1,2-*a*]­pyrimidin-4-one **9**. Compound **9** was obtained in a reaction of 5-fluoropyridin-2-amine **10** with dimethylmalonate **11** to yield 7-fluoro-2-hydroxy-pyrido­[1,2-*a*]­pyrimidin-4-one **12**, followed by chlorination with POCl_3_. Despite successful synthesis of the individual coupling partners, multiple attempts to achieve Suzuki coupling between activated intermediate 8*, generated by Miyaura borylation with Bis­(pinacolato)­dibor (B_2_Pin_2_), KOAc, and Pd­(dppf)_2_Cl_2_·DCM ((diphenylphosphin)­ferrocene = dppf), and compound **9** failed. Reaction conditions employing K_2_CO_3_ and Pd­(PPh_3_)_4_ in aqueous-acetonitrile mixtures repeatedly failed to produce the desired coupled product (**13**). These difficulties were attributed to structural modifications introduced by the cysteamine and Boc-protected groups, which significantly altered the reactivity compared to that of unmodified risdiplam. The synthesis route is summarized in Scheme 1.

**Scheme 1:**
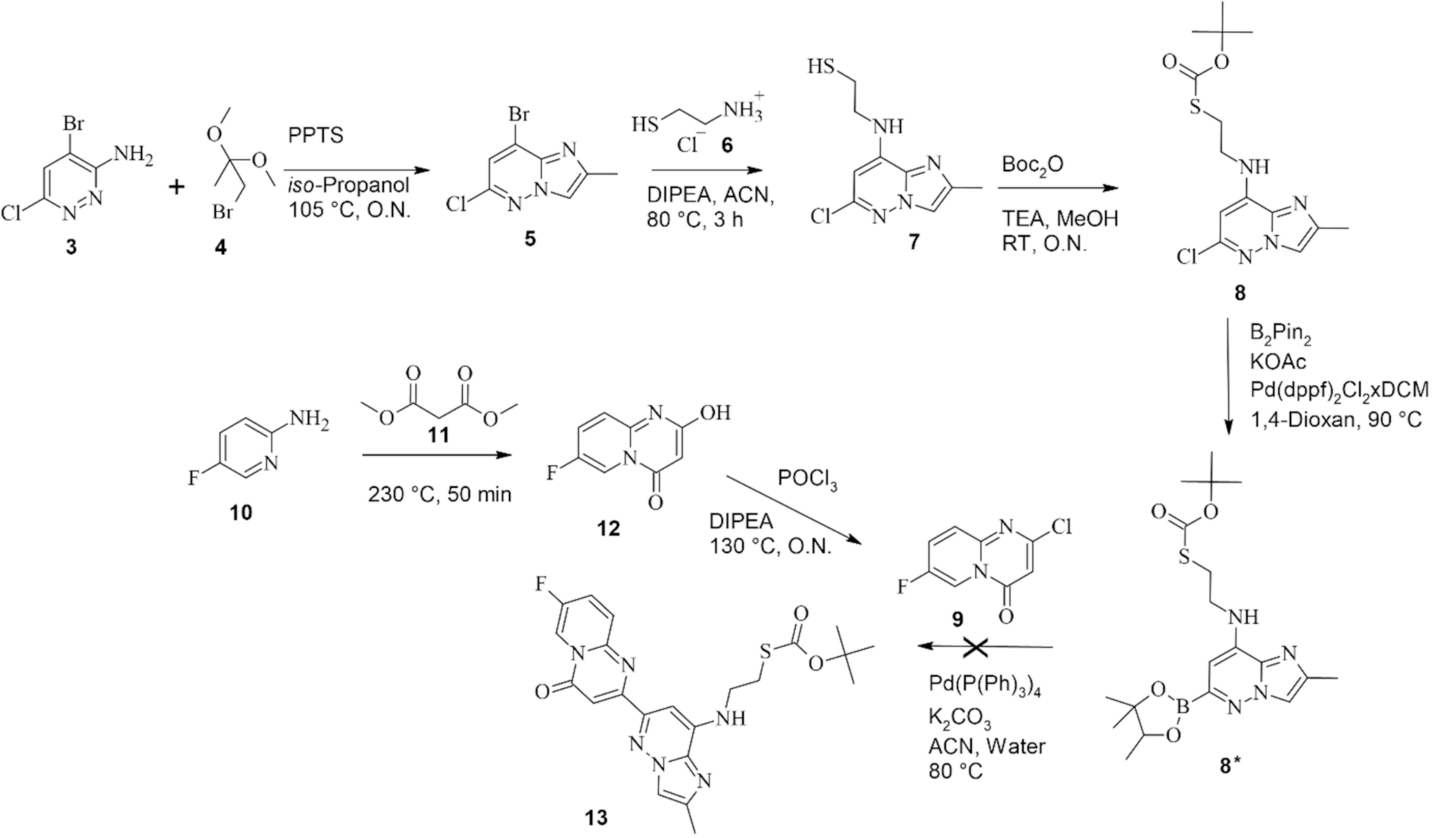
Initial Strategy to Introduce a PFB Exit Vector on Risdiplam^a^

a Compound **5** was prepared by successive SN reactions of precursors **3** and **4** in isopropanol with PPTS catalysis. Nucleophilic aromatic substitution of **5** with cysteamine **6** in acetonitrile yielded intermediate **7**. Boc protection of **7** in methanol delivered carbamate **8**, which was then converted to boronate ester 8* via Miyaura borylation. The intended coupling partner **9** was synthesized by alkylation of precursor **10** with dimethylmalonate **11** to give malonate **12**, followed by chlorination using POCl_3_. However, Suzuki–Miyaura cross-coupling between boronate 8* and aryl chloride **9** failed under the conditions tested.

Due to the unsuccessful attempt at modifying the imidazol­[1,2-*b*]­pyridazine moiety, we turned our attention to the piperazine group as an alternative anchoring point for the exit vector. A simplified risdiplam derivative lacking both methylation and spirocyclopropane substituent on the piperazine was employed.^4^ Initially, 6-chloro-2-methyl-imidazo­[1,2-*b*]­pyridazine **14** was activated through Miyaura borylation and then coupled via Suzuki cross-coupling to intermediate **9**, yielding 7-fluoro-2-(2-methylimidazo­[1,2-*b*]­pyridazin-6-yl)­pyrido­[1,2-*a*]­pyrimidin-4-on **15**. Subsequent aromatic substitution with *tert*-butyl piperazine-1-carboxylate **16**, followed by trifluoracetic acid (TFA)-mediated deprotection, provided 2-(2-methylimidazo­[1,2-*b*]­pyridazin-6-yl)-7-piperazine-1-yl-pyrido­[1,2-*a*]­pyrimidin-4-one **17**. This intermediate served as the anchor point for coupling with PFB-linked derivatives synthesized from decafluoro-biphenyl (‘PFB’) **2**. The coupling reaction employed *O*-(7-Azabenzotriazol-1-yl)-*N,N,N′,N*′-tetramethyluronium-hexafluorophosphate (HATU)-mediated peptide coupling, resulting in compounds with different linker lengths (**20**, **24a**-**c**, Ris-PFB 1–4). In the simplest case, coupling was carried out with 3-[2,3,5,6-tetrafluoro-4-(2,3,4,5,6-pentafluorophenyl)­phenyl]­sulfanyl-propanic acid **18**. This was synthesized by reacting PFB with 3-mercaptopropionic acid **19** in ACN at room temperature (RT) with triethylamine (TEA) present overnight (O.N.). In the simplest case, this yielded 2-(2-methylimidazo­[1,2-*b*]­pyridazine-6-yl)-7-[4-[3-[2,3,5,6-tetrafluoro-4-(2,3,4,5,6-pentafluoro-phenyl)­phenyl]­sulfanylpropanoyl]-piperazin-1-yl]­pyrido­[1,2-*a*]­pyrimidin-4-one **20**. Further compounds were synthesized with different linkers between the risdiplam structure and the PFB. For that, PFB was reacted with cysteamine hydrochloride **6** to yield PFB-derivative **21**. Specific reaction at the para-position is ensured by the directing effects of the fluoride-substitutes.^44^ Afterward, peptide coupling with various ^*t*^Bu-protected diacids **22 a**-**c** was carried out. After deprotection using TFA in DCM, species with additional linkers **23 a**-**c** were obtained. Those were then coupled to **17** using the same HATU-based peptide coupling to give **24 a**-**c**. The reaction scheme is shown in Scheme 2.

**Scheme 2:**
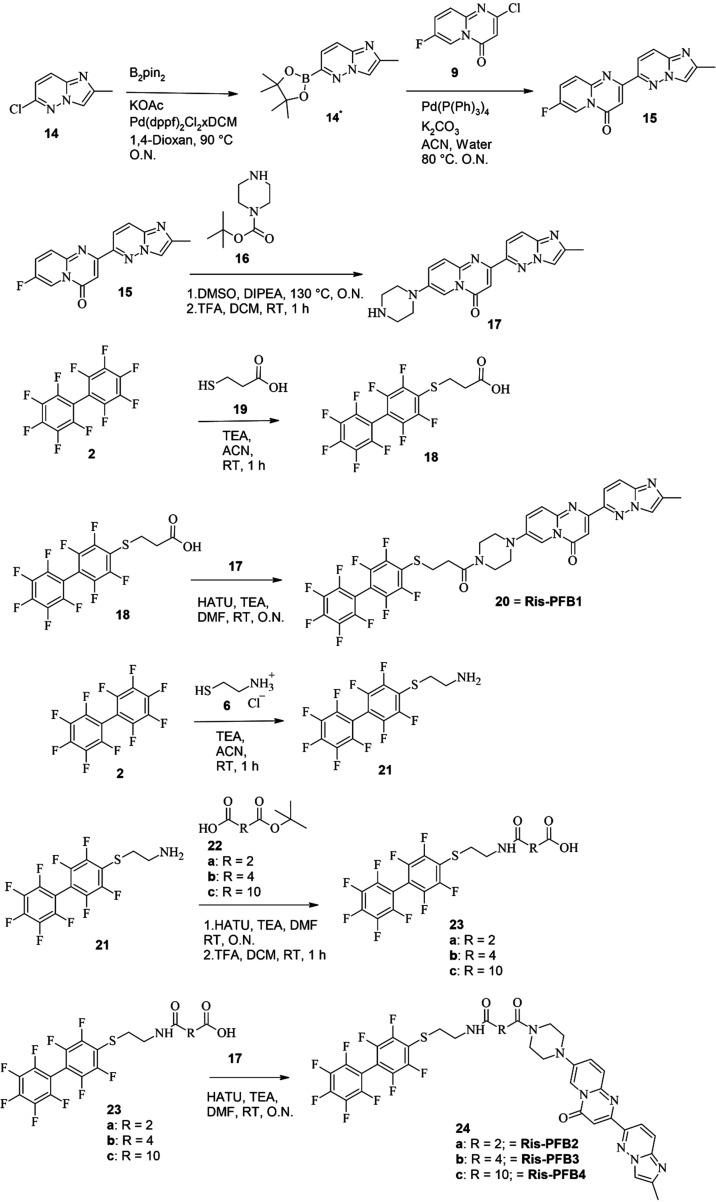
Synthesis of Risdiplam–PFB Chimeric Molecules^a^

a Boronate ester **14** was generated via Miyaura borylation and then coupled to aryl chloride **9** by Suzuki cross-coupling to afford intermediate **15**. Heating **15** with linker **16** in DMSO at 130 °C, followed by TFA-mediated deprotection in DCM, produced key intermediate **17**. Parallel HATU-mediated amide couplings of **17** with PFB-derivative acids **18** or **23** a–c furnished the final chimeras **20** and **24 a–c** (Ris-PFB 1–4). The PFB acids **18** and **23 a–c** were prepared either by thiol coupling of mercaptopropionic acid **19** or cysteamine hydrochloride **6** to t-butyl-protected diacids **22 a–c**, followed by HATU activation and global deprotection with TFA/DCM.

### *In Silico* Physicochemical and ADME Characterization

Physicochemical properties strongly shape pharmacokinetic/pharmacodynamic (PK/PD) behavior.^50,51^ For classical small molecules, optimizing lipophilicity, molecular weight, and hydrogen-bonding capacity within Rule-of-5 (Ro5) guidelines has been central to achieving acceptable ADME (Absorption, distribution, metabolism, and elimination) and oral exposure.^52^ By contrast, beyond-Ro5 (bRo5) modalities, most notably heterobifunctional chemotypes such as PROTACs, pose distinct permeability and formulation challenges.^53,54^ Recent AI-based ADME tools enable rapid *in silico* triage; here we used SwissADME^55^ (accessed on Jul-22–25) to profile our PROTAC-like Ris-PFB series and, for context, five clinical PROTACs (BGB-16673,^45^ ARV-110,^46^ ARV-471,^47^ ARV-776,^48^ and CFT1946 ^49^ (Figure 2)). We evaluated Consensus LogP, Topological Polar Surface Area (TPSA), predicted aqueous solubility (ESOL) LogS, rotatable bonds, gastrointestinal (GI) absorption, blood–brain barrier (BBB) permeability, cytochrome p (CYP) inhibition, and P-glycoprotein (P-gp) substrate status (Tables 1, SI1, and SI2).

**Figure 2:**
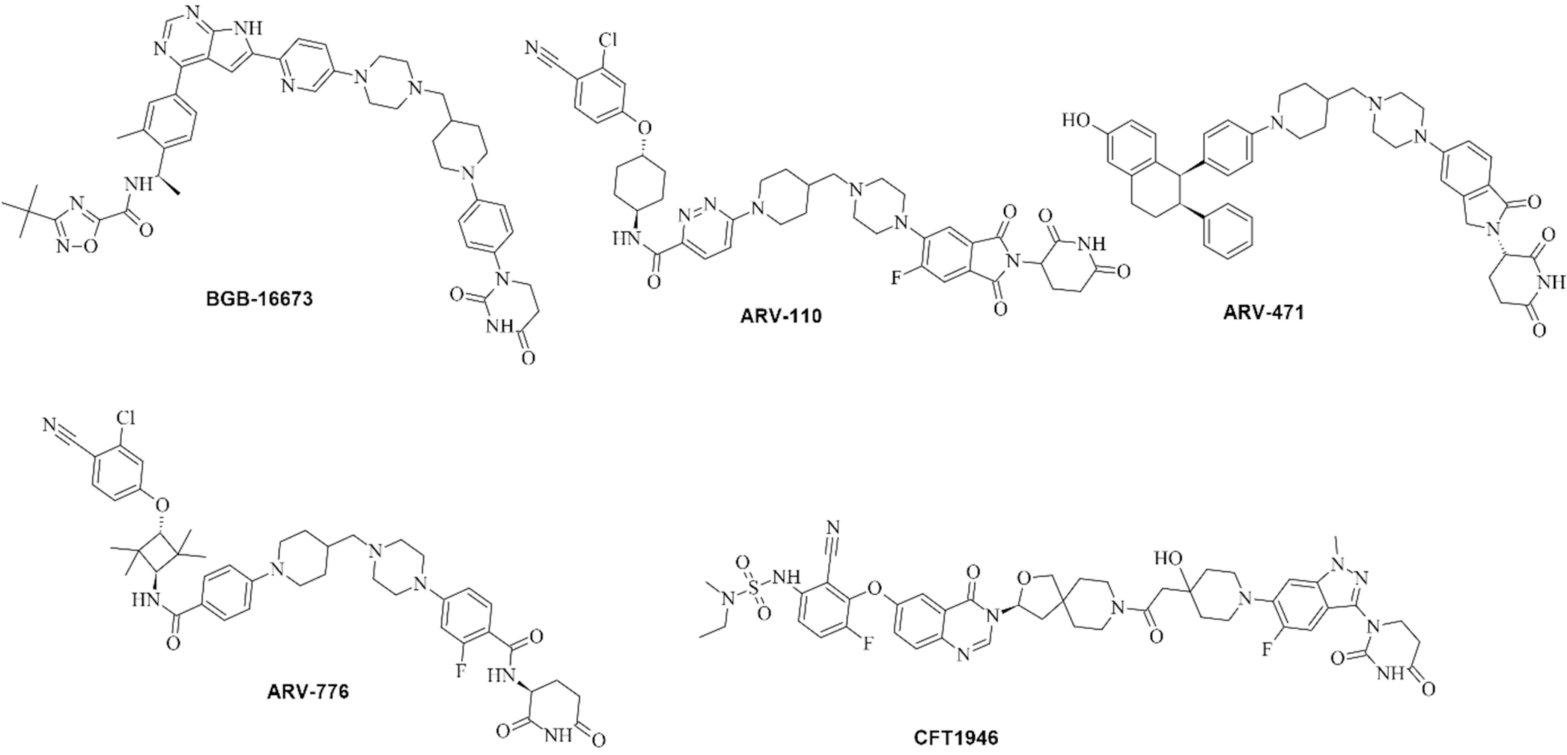
PROTACs in clinical trials that were used to reference the SwissADME predictions of Ris-PFB 1–4. From left to right and from top to bottom: BGB-16673, ARV-110, ARV-471, ARV-776, and CFT1956.^45–49^

**Table 1:** Predicted Results from the AI-Based DMPK Analysis^a^

molecule	MW	HBA	HBD	Con_LogP	RotB	TPSA	ESOL_LogS	CYP3A4	CYP1A2
Ro5	≤500	≤10	≤5	≤5	≤10	≤140	≥-4		
Beyond_Ro5	>500	≤15	≤6	≤10	≤20	≤250	≥-10		
BGB-16673	851	10	3	4.82	12	182	–8,62	yes	no
ARV-110	812	11	2	3.5	10	181	–6,93	yes	no
ARV-471	724	5	2	5	7	96	–8,2	yes	no
ARV-776	808	9	3	4.55	13	156	–7,96	yes	no
CFT1946	958	15	3	3.06	12	246	–6,96	yes	no
Ris-PFB1	764	14	0	6.84	8	113	–7,24	yes	no
Ris-PFB2	835	15	1	6.43	12	143	–6,91	yes	no
Ris-PFB3	863	15	1	7.04	14	143	–7,38	yes	no
Ris-PFB4	947	15	1	9.13	20	143	–9,52	yes	no

a Ro5 and bRo5 were reported by Cantrill et al.^53^

All test compounds reside in bRo5 space with high molecular weight and polarity. Ris-PFB1–4 display MW 764–947 g/mol, TPSA 113–143 Å^2^, and 8–20 rotatable bonds, consistent with limited passive permeability and non-BBB permeation. Consensus lipophilicity is elevated (cLogP_cons 6.4–9.1), and ESOL LogS indicates poor to very poor intrinsic aqueous solubility (≈ −6.9 to −9.5). The clinical comparators display broadly similar patterns (MW 724–958 g/mol; TPSA 96–246 Å^2^; cLogP_cons ≈ 3.1–5.0) and likewise poor predicted solubility (ESOL ≈ −6.9 to −8.6). SwissADME predicts CYP3A4 inhibition for all compounds and no CYP2A1 inhibition. These *in silico* results situate the Ris-PFBs within the expected heterobifunctional drug metabolism and pharmacokinetics (DMPK) envelope and support development strategies emphasizing dose-sparing (enabled here by chemical recruitment) alongside delivery/formulation solutions and medicinal-chemistry optimization to overcome solubility and permeability constraints.

MW: Molecular Weight; HBA: H-Bond Acceptors; HBD: H-Bond Donors; Con_LogP: consensus LogP; RB: Rotatable Bonds; TPSA: Topological Polar Surface Area; ESOL_LogS: predicted aqueous solubility (Log10 mol/L). The completed prediction can be found in Supporting Information Tables SI1 and SI2.

## Biology

### Functional Evaluation of New Ris-PFB Derivatives

Functional assessment of the newly synthesized Ris-PFB derivatives (Ris-PFB1–4) employed a well-established *SMN2* splicing model system (pCI_SMN2; Addgene #72287).^40,56,57^ To quantify alternative splicing, primer pairs specific to different *SMN2* splice variants were designed for quantitative reverse transcription PCR (qRT-PCR). As shown in Figure 3A, RT-PCR gel analysis confirmed the expected products: *SMN2_7–8* (primer pair 1; 85 bp, exon 7/8 junction) and *SMN2_6–7–8* (primer pair 2; 141 bp, exons 6–7–8), both reflecting the unspliced isoforms. Dose–response experiments in risdiplam-treated cells demonstrated similar EC_50_ values for unspliced *SMN2* detection using primer pairs 1 and 2, validating their use in subsequent qRT-PCR assays (Figure 3B). Although specific amplification of the *SMN2_*Δ*7* variant remained challenging, primer pair 3 yielded a mixture amplification of *SMN2_6–7–8* (141 bp) and *SMN2_*Δ*7* (87 bp), suitable for qualitative assessments of splicing efficiency by RT-PCR gel analysis.

**Figure 3:**
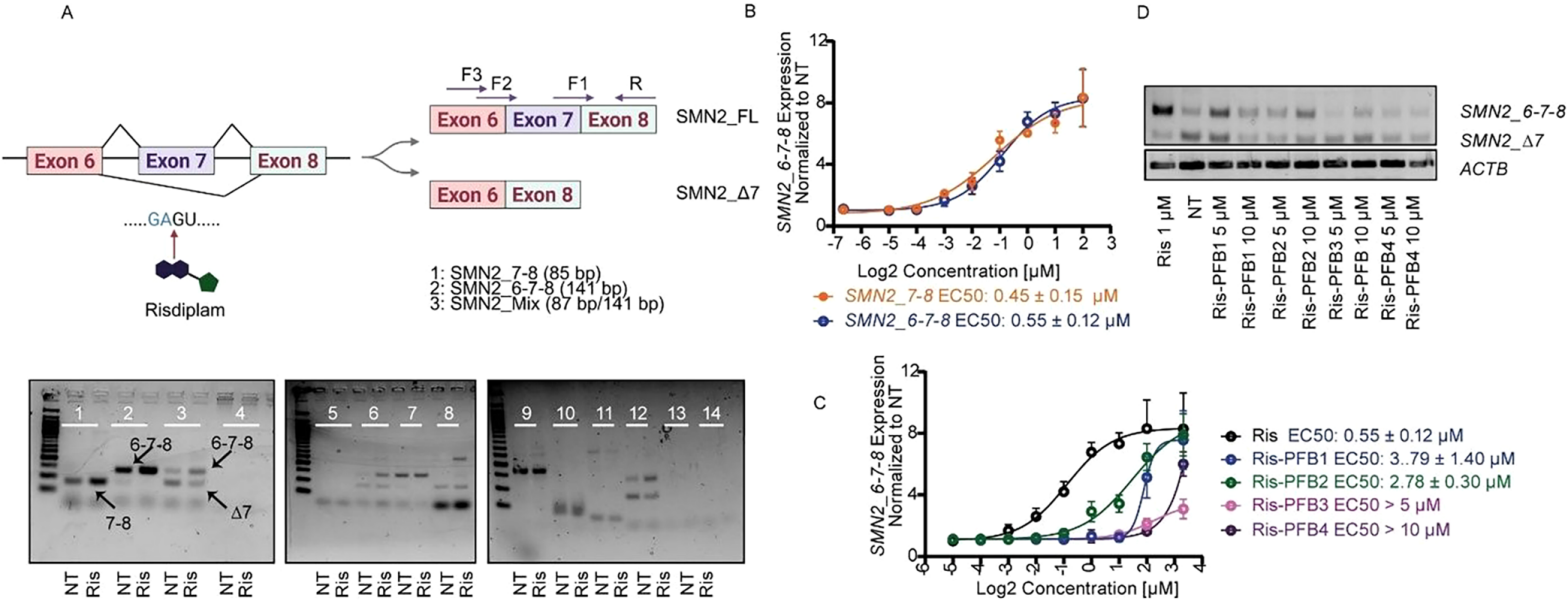
Functional evaluation of new Ris-PFB derivatives. (A) Schematic representation of *SMN2* exon 7 inclusion and exclusion events and design for primer pairs used for qRT-PCR. Risdiplam enhances U1 snRNP binding at the GA/GU motif upstream of exon 7, thereby promoting exon inclusion. Fourteen primer pairs were initially tested; among them, primer pair 1 (*SMN2_7–8*/85 bp), primer pair 2 (*SMN2_6–7–8*/141 bp), and primer pair 3 (*SMN2_Mix*, *SMN2_*Δ*7*/87 bp, and *SMN2_6–7–8*/141 bp) were validated by RT-PCR gel analysis and selected for further experiments. NT: nontreated control, Ris: risdiplam-treated cells (1 μM). (B) Dose–response analysis of *SMN2_7–8* and *SMN2_6–7–8* transcripts in HEK293T cells transfected with the pCl-SMN2 vector and treated with increasing concentrations of risdiplam. Transcript levels were quantified by qRT-PCR using primer pairs 1 (*SMN2_7–8*) and 2 (*SMN2_6–7–8*). EC_50_ values were 0.45 ± 0.15 and 0.55 ± 0.12 μM, respectively. (C) Comparative dose–response of *SMN2_6–7–8* expression following treatment with risdiplam or Ris-PFB1–4. Risdiplam showed the highest potency (EC_50_ = 0.55 ± 0.12 μM), followed by Ris-PFB2 (2.78 ± 0.30 μM) and Ris-PFB1 (3.79 ± 1.40 μM). Longer linkers (Ris-PFB3 and 4) showed significantly reduced or undetectable activity. (D) RT-PCR gel analysis of *SMN2* splicing patterns using primer pair 3 confirms the qRT-PCR findings. Bands corresponding to *SMN2_6–7–8* and *SMN2_*Δ*7* are indicated. β-actin (ACTB) serves as a loading control. NT: nontreated control; Ris: risdiplam (5 and 10 μM).

We next compared the efficacy of Ris-PFB1–4 in modulating *SMN2_6–7–8* levels. HEKT293T cells were treated with concentration gradients of each derivative, and unspliced transcript levels were quantified by qRT-PCR using primer pair 2. Incorporation of the PFB exit vector reduced the potency relative to risdiplam (Figure 3C). Among the derivatives, Ris-PFB1 and Ris-PFB2, which bear the shortest linkers, retained the highest potency, with EC_50_ values around 3 μM. Increasing the linker length progressively decreased efficacy: Ris-PFB3, which includes two additional methylene units, showed a marked potency loss, and Ris-PFB4 (ten-methylene linker) was inactive (Figure 3C), a trend corroborated by gel electrophoresis results (Figures 3D and SI1). These results indicate that linker length is a critical determinant of the intrinsic activity of PFB-conjugated risdiplam derivatives in modulating *SMN2* splicing.

### Optimization of dCas13^FCPF^ and crRNA Design for Chemically Induced SMN2 Splicing Modulation

Building on our Chem-CRISPR/dCas9^FCPF^ platform—where epigenetic modulation depended critically on guide RNA positioning^36^ we optimized crRNA target sites for dCas13^FCPF^ system. We designed three crRNAs targeting distinct regions of *SMN2* pre-mRNA: Region 1 upstream of the risdiplam binding motif, Region 2 overlapping the motif, and Region 3 downstream of the motif (Figure 4A).

**Figure 4:**
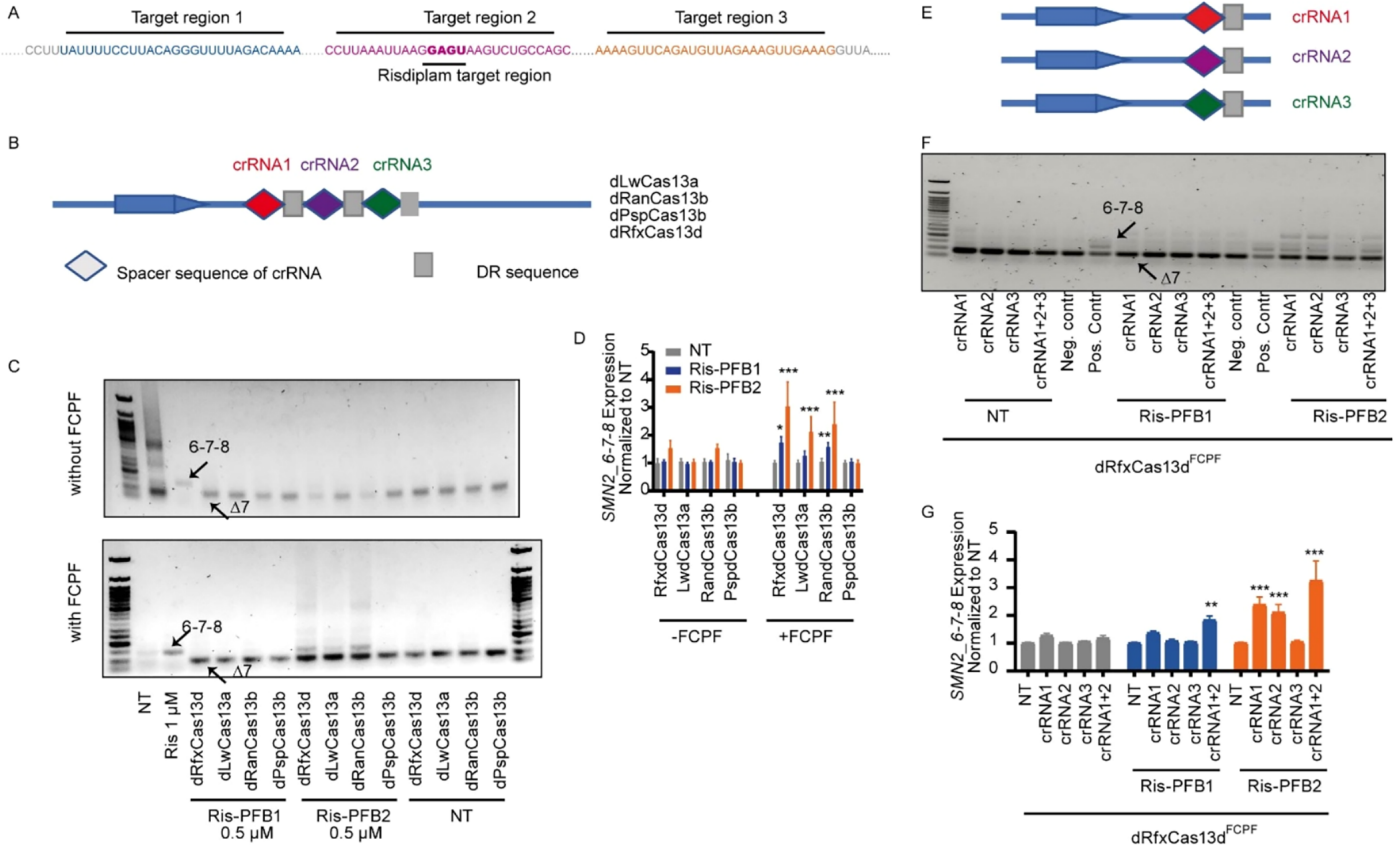
Optimization of the dCas13FCPF and crRNA Design for chemically induced SMN2 splicing modulation. (A) Sequence layout of *SMN2* pre-mRNA and positioning of crRNA1–3. crRNA target regions were selected upstream (crRNA1), overlapping (crRNA2), and downstream (crRNA3) of the risdiplam binding motif. Risdiplam-targeting motif is highlighted. (B) Schematic of multiplexed crRNA array and Cas13 constructs used in this study. Four catalytically inactive Cas13 variants (dLwaCas13a, dRanCas13b, dPspCas13b, and dRfxCas13d) were tested, with each fused C-terminally to the FCPF tag. (C) RT-PCR gel analysis comparing the activity of dCas13 variants with or without FCPF tagging in the presence of Ris-PFB1 or Ris-PFB2 (0.5 μM). *SMN2_6–7–8* and *SMN2_*Δ*7* splice variants are indicated. NT: nontreated; Ris: risdiplam-treated control (1 μM). (D) qRT-PCR quantification of *SMN2_6–7–8* levels from (C). dRfxCas13d^FCPF^ and dRanCas13b^FCPF^ showed significant activation in response to Ris-PFB2. Bars represent the mean ± SD (*n* = 6). (E) Diagram showing the expression of individual or multiplexed crRNAs to evaluate positional effects. (F) RT-PCR validation of crRNA-specific activity using dRfxCas13d^FCPF^ in combination with Ris-PFB1 or Ris-PFB2. crRNA1 + 2+3: crRNA array containing crRNA1, crRNA2, and crRNA3. (G) qRT-PCR quantification of (F). Only crRNA1 and crRNA2 significantly enhanced *SMN2_6–7–8* levels with Ris-PFB2. Data represent mean ± SD (*n* = 6). ***p < 0.001, **p < 0.01, *p < 0.05 (two-way ANOVA with *t* test).

To identify an optimal Cas13 scaffold, we compared four catalytically inactive Cas13 variants: *Leptotrichia wadei* Cas13a (LwaCas13a), *Riemerella anatipestifer* Cas13b (RanCas13b), *Prevotella sp. P5–125* Cas13b (PspCas13b), and *Ruminococcus flavefaciens* Cas13d (RfxCas13d).^18,22,58–60^ Each variant was C-terminally tagged with FCPF by insertion of a chemically synthesized DNA fragment (5′-TTC TGC CCA TTC-3′), as we reported previously.^36^ We also generated constructs to coexpress a multiplexed crRNA array comprising three associated DR spacer units (Figure 4B), which are required for proper pre-crRNA processing and Cas13 recognition.^18^

For ligand screening, we selected a concentration of 0.5 μM for both Ris-PFB1 and Ris-PFB2, which exhibited minimal baseline activity in previous assays (Figure 3C). In HEK293T cells, neither vehicle (nontreatment) nor expression of dCas13 lacking FCPF altered *SMN2* splicing, confirming that both the FCPF tag and small-molecule recruitment are required (Figure 4C). Upon treatment with 0.5 μM Ris-PFB2, cells expressing dRfxCas13d^FCPF^ or dRanCas13b^FCPF^ exhibited the largest increase in unspliced *SMN2_6–7–8* levels. A modest effect was observed with dLwaCas13a^FCPF^, whereas dPspCas13b^FCPF^ was inactive (Figure 4C). In contrast, Ris-PFB1 did not elicit a clear response with any dCas13^FCPF^ variant. These results, corroborated by qRT-PCR, demonstrate that dRfxCas13d^FCPF^ and dRanCas13b^FCPF^ in combination with Ris-PFB2 are the most effective scaffolds for chemically induced *SMN2* splicing modulation. We selected dRfxCas13d^FCPF^ for further development of our Chem-CRISPR/dCas13^FCPF^ system due to its smaller size and lower reported toxicity,^61^ making it more suitable for *in vivo* applications (Figure 4D).

To refine the guide specificity, we next expressed each crRNA individually with dRfxCas13d^FCPF^ and evaluated splicing modulation by Ris-PFB1 and Ris-PFB2. Consistent with prior results, in the presence dRfxCas13d^FCPF^, vehicle (no ligand) or Ris-PFB1 produced no detectable change in *SMN2* splicing with any single crRNA or their combination (Figure 4F). In contrast, Ris-PFB2 significantly increased unspliced *SMN2_6–7–8* levels when directed by crRNA1 or crRNA2, but not by crRNA3. These findings, validated by RT-PCR, underscore the critical role of crRNA target-region selection in achieving efficient chemically induced RNA modulation. Accordingly, we selected the combination of crRNA1 and crRNA2 to guide Ris-PFBs in targeting *SMN2* pre-mRNA in subsequent experiments (Figure 4E–G).

### Chem-dRfxCas13d^FCPF^ Significantly Enhances Potency and Selectivity of Risdiplam

Using the optimized dRfxCas13d^FCPF^ construct and crRNAs, we next assessed the potency and specificity of Ris-PFB2 for *SMN2* splicing modulation. Under dRfxCas13d^FCPF^-crRNAs guidance, Ris-PFB2 achieved a nearly 500-fold increase in potency compared to risdiplam alone, as indicated by marked EC_50_ shifts in qRT-PCR assays (Figure 5A). In contrast, cells expressing dRfxCas13d lacking the FCPF tag showed no enhancement (Figure 5B), confirming that site-specific covalent recruitment is required.

**Figure 5:**
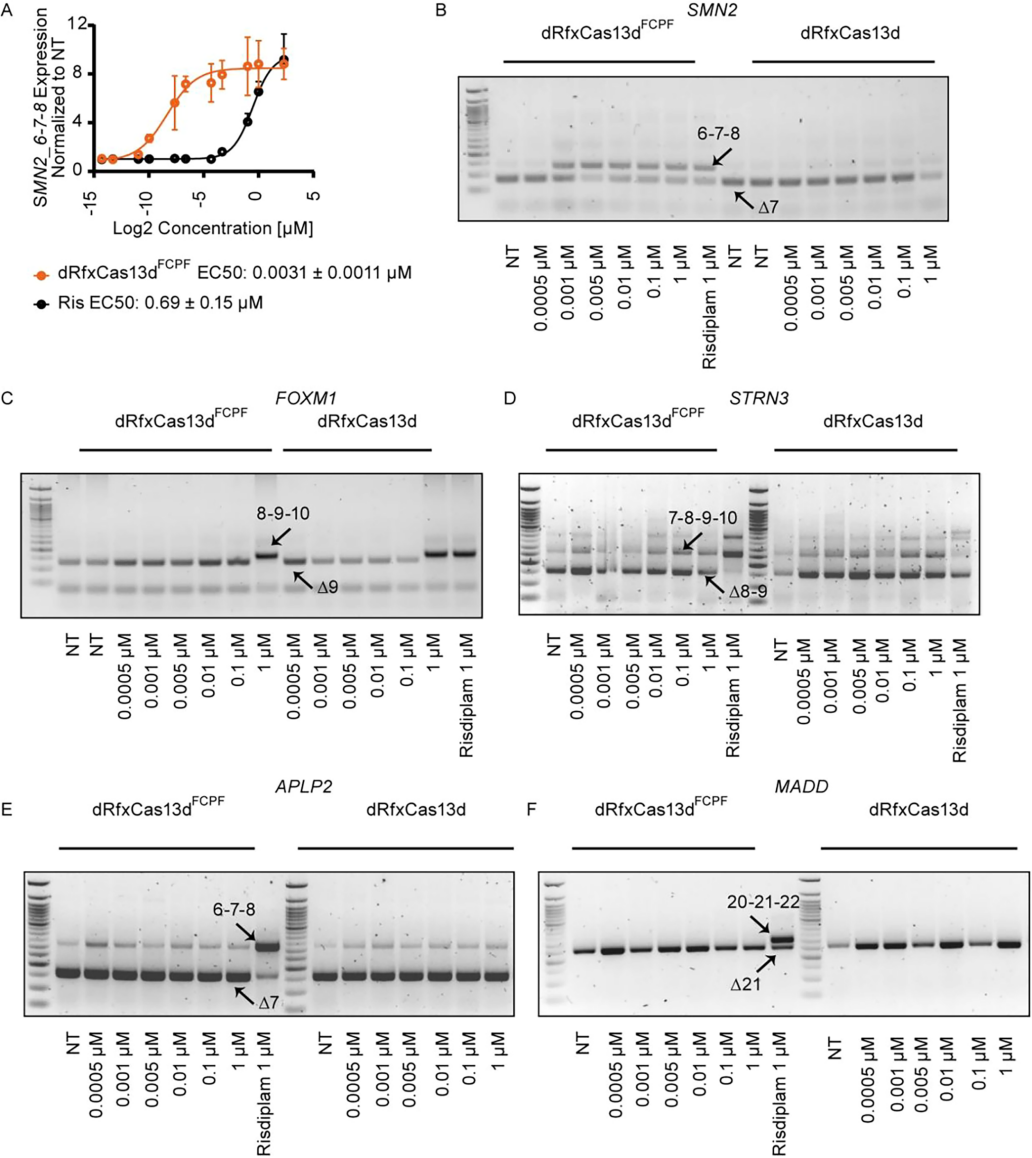
Chem-dRfxCas13d^FCPF^ significantly enhanced the target selectivity of risdiplam. (A) Dose–response comparison of risdiplam and Ris-PFB2 in *SMN2_6–7–8* splicing activation analyzed by qRT-PCR. Cells expressing dRfxCas13d^FCPF^ showed a dramatic potency enhancement with Ris-PFB2 (EC_50_ = 0.0031 ± 0.0011 μM), compared to free risdiplam (EC_50_ = 0.69 ± 0.15 μM). (B) RT-PCR validation of *SMN2* splicing patterns in cells expressing either dRfxCas13d^FCPF^ or dRfxCas13d (without the FCPF tag) across a range of Ris-PFB2 concentrations. (C–F) Off-target splicing analysis of four known risdiplam-sensitive genes: *FOXM1* (C), *STRN3* (D), *APLP2* (E), and *MADD* (F). Cells were treated with increasing concentrations of Ris-PFB2, and splicing patterns were analyzed by RT-PCR. Risdiplam (1 μM) was used as a positive control in each panel. NT: nontreated control.

To evaluate off-target effects, we first monitored four transcripts previously reported as risdiplam-sensitive and bearing GA/GU motifs: *FOXM1, STRN3, APLP2*, and *MADD*.^15,62^ In HEK293T cells expressing dRfxCas13d^FCPF^, treatment with Ris-PFB2 produced no detectable splicing changes in these transcripts (Figure 5C–F). We next designed triplicate crRNA sets per gene (Figure 6A). As expected, the CRISPR/dRfxCas13d^FCPF^ system modulated alternative splicing only when guided by cognate crRNAs and in the presence of Ris-PFB2 (0.01 μM); omission of the ligand or crRNAs had no effect (Figure 6B–D). These data demonstrate sequence-dependent target engagement and high locus selectivity, achieved by positioning the small-molecule modulator precisely at a defined RNA site via programmable crRNA guidance.

**Figure 6:**
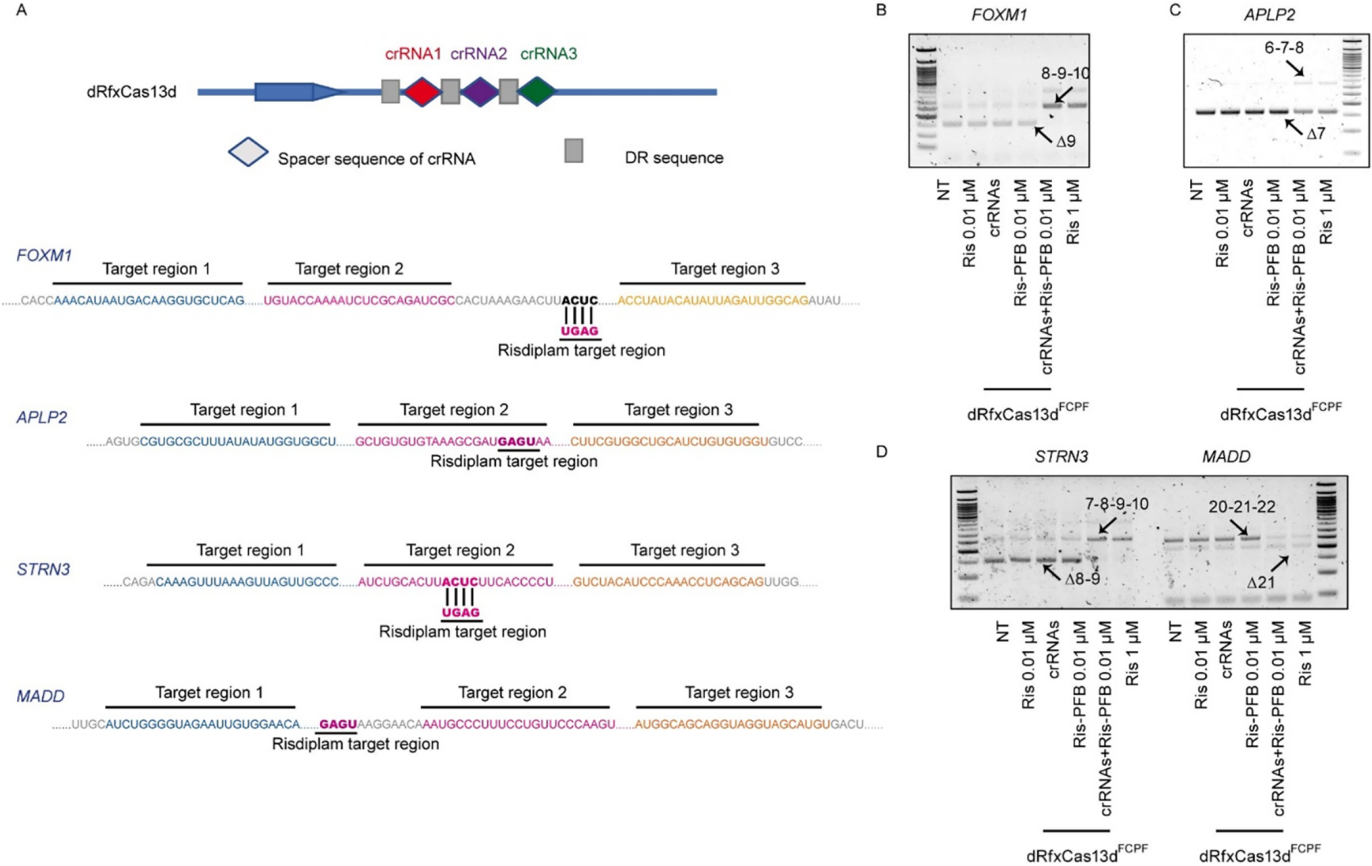
Chem-dRfxCas13d^FCPF^ affects alternative splicing in a sequence-dependent manner. (A) Sequence layout of *FOXM1, APLP2, STRN3, and MADD* pre-mRNA and positioning of crRNA1–3. Risdiplam-targeting motif is highlighted. (B–D) RT-PCR validation of splicing patterns in cells under various conditions: NT, risdiplam 0.01 μM, risdiplam 1 μM, or cells expressing dRfxCas13d^FCPF^ in the presence of crRNAs, Ris-PFB 0.01 μM, or in combination for (B) FOXM1, (C) APLP2, and (D) STRN3 and MADD.

## Discussion

Off-target effects remain a central obstacle in the development of small-molecule therapeutics, particularly in DNA/RNA-targeted drug discovery.^15,62^ Unlike proteins, many RNA motifs are conserved across transcripts, complicating sequence-specific recognition and increasing the risk of activity at unintended loci.^63^ This challenge is exemplified by small-molecule splicing modulators for SMA: both risdiplam (approved) and branaplam have been reported to alter splicing at numerous GA/GU motifs transcriptome-wide.^15,62^ In the case of branaplam, such broad, nonspecific activity has been associated with dose-limiting toxicities, and clinical programs in SMA and Huntington’s disease were discontinued.^15,62,64,65^

Indeed, compounds that globally engage spliceosomal components or splice-associated RNA motifs frequently induce widespread alternative splicing changes.^62^ While valuable as research tools, their lack of sequence-level specificity hinders safe therapeutic application.^15,62,66^ This observation underscores the need for programmable platforms that combine chemical activity and molecular specificity.

CRISPR/Cas13 systems offer an attractive solution because they provide programmable RNA recognition, do not depend on host DNA repair machinery, and modulate endogenous RNAs without altering the genome.^17,28–30^ However, their therapeutic translation is limited by several factors: the large size of Cas13 fusion proteins complicates delivery;^31^ catalytically active Cas13 carries a risk of collateral RNA cleavage;^67,68^ and most dCas13 effectors rely on noncovalent, bulky protein fusions that may interfere with endogenous RNA metabolism or localization.^29,69^

Our Chem-dRfxCas13d^FCPF^ system addresses these issues by integrating the precision of CRISPR-based RNA recognition with the modularity and tunability of chemically induced modulation. By covalently recruiting small-molecule effectors such as Ris-PFB2 to dCas13^FCPF^ via π-clamp-mediated binding, we demonstrated substantial improvement in both potency and selectivity. This system enables targeted RNA modulation at significantly lower compound concentrations (over 500-fold lower than free risdiplam) while eliminating detectable off-target activity at known risdiplam-sensitive transcripts. Furthermore, we establish that guide RNA positioning and Cas13 scaffold selection critically influence functional output with dRfxCas13d^FCPF^ offering an optimal balance of activity, compact size, and minimal toxicity.

Because Chem-CRISPR/dCas13^FCPF^ is modular at two layers, guide-defined addressability, and exchangeable small-molecule effector, it can, in principle, be repurposed beyond risdiplam to create locus-selective, chemically induced proximity at diverse RNA sites. By swapping the PFB-tagged ligand, the platform could recruit or inhibit RNA-modulating proteins, for example, the m^6^A machinery: METTL3 inhibitors (e.g., STM2457, UZH1a/b)^70,71^ to locally suppress m^6^A deposition, or demethylase inhibitors (e.g., FTO inhibitor FB23–2; ALKBH5 tool inhibitors)^72,73^ to enhance m^6^A at selected transcripts. Likewise, small-molecule modulators of splicing-regulatory kinases (e.g., SRPK or CLK inhibitors)^74,75^ could be tethered to bias splice-factor engagement near programmed pre-mRNA sites. Under crRNA guidance, dCas13^FCPF^ positions these effectors at virtually any RNA locus, offering a general route to site-restricted RNA modification or processing while reducing free-ligand exposure; the ultimate performance will depend on effector access and local RNP context at the targeted site.

Heterobifunctional small molecules are central to the Chem-CRISPR/dCas13^FCPF^ concept, but typically occupy bRo5 space, exceeding classical limits on molecular weight and polar surface area.^76^ This profile depresses passive permeability, heightens susceptibility to efflux and first-pass metabolism, and complicates oral absorption.^77–80^ Even so, our data demonstrate a dose-sparing effect: covalent recruitment of the ligand in the immediate vicinity of the programmed RNA site markedly lowers the amount of small molecule required to achieve the desired modulation (∼500-fold versus free risdiplam under matched conditions; Figure 5A).

Although protein/toxin-based vehicles^81^ and adeno-associated viruses (AAVs)^82^ have enabled *in vivo* delivery of CRISPR/Cas systems, including Cas13 payloads, effective coadministration of dCas13 with a relatively bulky, low-permeability ligand such as Ris-PFB2 remains challenging. Lipid nanoparticles (LNPs) provide a practical route, having been used to deliver nucleic acids, proteins/RNPs, and small molecules.^83–85^ Prior to evaluation in patient-derived induced pluripotent stem cells (iPSC) SMA^86^ and SMNΔ7 mouse models,^87^ development of LNP, either single-vehicle coformulation of a preassembled dCas13–crRNA–ligand complex,^88,89^ or dual-vehicle or sequential dosing (vector/LNP for dCas13–crRNA followed by ligand). In parallel, medicinal-chemistry optimization, including permeability-enhancing substitutions, prodrug designs, and linker/exit vector tuning (including rigidification/macrocyclization), together with salt or amorphous solid dispersion (ASD) formulation, may further reduce exposure requirements while preserving on-target potency.

## Conclusion

The Chem-CRISPR/dCas13^FCPF^ platform provides a generalizable strategy for small-molecule–controlled, programmable RNA modulation. By coupling CRISPR-level addressability with covalent chemical recruitment, transcript-specific splicing control can be achieved at markedly reduced ligand doses and with high specificity in human cells. *In silico* ADME profiles of the Ris-PFB ligands are consistent with heterobifunctional chemotypes (e.g., low BBB permeability and CYP3A4 liability), liabilities that can be managed through dose-sparing, medicinal-chemistry optimization, and delivery/formulation strategies. Recent clinical setbacks with some RNA-targeting splicing modulators attributed to insufficient specificity highlight the need for locus-restricted mechanisms; the programmability of Chem-CRISPR/dCas13^FCPF^ directly addresses this requirement and is readily extendable beyond SMN2 by crRNA redesign and effector swapping. Collectively, these results establish a modular, chemically controllable dCas13 modality with clear potential for therapeutic RNA modulation and a rational roadmap toward *in vivo* validation, delivery solutions, and medicinal-chemistry optimization.

## Experimental Section

### Chemistry

Solvents and reagents were provided by commercial suppliers, meeting at least reagent-grade standards, or were purified according to established methods before use if required. A scale with a precision of 0.1 mg was used to weigh chemicals. Reactions conducted under nitrogen protection were purged with nitrogen gas to eliminate the presence of oxygen. For these procedures, predegassed anhydrous solvents were utilized, unless stated otherwise. A PuriFlash XS520Plus system from Advion Interchim was employed to monitor chemical reactions and used for purification using reversed-phase flash column chromatography (RP FCC) and a UV detector with the usage of a 254 nm channel. A PF-30C18HP-F0012 column was used. Elution was performed with 1% formic acid in water and acetonitrile. General method:(1) 0:00 min–2:50 min ACN/water 5:95; (2) 2:50 min–6:50 min increase to ACN/water 100:0; (3) Hold of 100:0 ACN/water. Method used for Ris-PFB 3: (1) 0:00 min–0:30 min ACN/water 5:95; (2) 0:30 min–3:00 min increase to ACN/water 70:30; (3) 3:00 min–5:00 min hold of 70:30 ACN/water; (4) 5:00 min–6:00 min increase to ACN/water 100:00; (5) Hold of 100:0 ACN/water. This method was later shown to be superior for other reactions as well. Normal phase (NP) silica column chromatography was carried out using a silica gel column and the given solvents. NMR spectroscopy was performed on Bruker instruments ranging from 300 to 500 MHz. The purities of compounds were verified through HPLC “LC/MS” analysis. A Poroshell 120 EC-C18 (Agilent, 3 × 150 mm^2^, 2.7 μm) reversed-phase column was used, and elution was performed with 0.1% formic acid in water (A) and 0.1% formic acid in acetonitrile (B) as a mobile phase. The following gradients were used: Method 1 (M1): 0 min: 5% B—2 min: 80% B—5 min: 95% B—7 min: 95% B (flow rate of 0.6 mL/min). Method 2 (M2): 0 min: 5% B—2.8 min: 75% B—7.2 min: 100% B—10 min: 100% B (flow rate of 0.6 mL/min). UV-detection was performed at 254 nm. All nonintermediate compounds are >95% pure by HPLC analysis. Figures were made using various programs, such as Biorender, ChemSketch, and Adobe Illustrator.

#### Synthesis of 8-Bromo-6-chloro-2-methyl-imidazo­[1,2-*b*]­pyridazine **5**

1999.6 mg (9.59 mmol; 1.00 equiv) of 3-amino-4-bromo-6-chloropyradizine was suspended in 100 mL of isopropanol, and then 1.58 mL (11.51 mmol; 1.20 equiv) of 1-bromo-2,2-dimethoxypropane and 19.6 mg (0.67 mmol; 0.07 equiv) of PPTS were added. The mixture was heated to 105 °C for 20 h and filtered after cooling to RT. The filter cake and filtrate were purified separately using RP FCC, and the product was recovered as 1274.3 mg of a yellow solid with a yield of 53.91%.

^1^H NMR (400 MHz, DMSO-*d*_6_): δ 2.358 (s, H_A_, 3 H); 7.837 (s, H_Y_, 1 H); 8.220 (s, H_Z_, 1 H).

^13^C NMR (400 MHz, DMSO-*d*_6_): δ 14.468; 116.519; 120.343; 122.446; 135.812; 144.322; 144.480.

ESI-MS: C_7_H_5_BrClN_3_ 246.49 (calc.), *m*/*z* = 247.85 [M + H]^+^ (found).

HRMS: C_7_H_5_BrClN_3_ 246.4917 (calc.), *m*/*z* = 247.4908 [M + H]^+^ (found).

LC-ESI-MS: C_7_H_5_BrClN_3_ 246.49 (calc.), *m*/*z* = 245.95 [M-H]^−^ (found).

#### Synthesis of 2-[(6-Chloro-2-methyl-imidazo­[1,2-*b*]­pyridazin-8-yl)­amino]­ethanethiol **7**

242.8 mg (0.99 mmol; 1.00 equiv) of 8-bromo-6-chloro-2-methyl-imidazo­[1,2-*b*]­pyridazine were dissolved in 10 mL of ACN and 0.28 mL (1.58 mmol; 1.60 equiv) of DIPEA, 145.7 mg (1.28 mmol; 1.30 equiv) of cysteamine hydrochloride was added, and the solution was heated to 80 °C for 3 h. After being diluted with MeOH, the reaction solution was adsorbed on Celite and purified using RP FCC. The product was recovered as a white solid in quantitative yield.

^1^H NMR (300 MHz, DMSO-*d*_6_): δ 2.353 (s, H_A_, 3 H); 3.090 (t, ^3^ *J* = 5.9 Hz, H_B_, 2 H); 3.438 (t, ^3^ *J* = 6.4 Hz, H_C_, 2 H); 7.244 (s, H_X_, 1 H); 8.032 (s, H_Y_, 1 H); 8.267 (s, H_Z_, 1 H).

^13^C NMR (400 MHz, DMSO-*d*_6_): δ 14.349; 27.773; 110.434; 115.037; 134.703; 139.836; 142.621; 144.991; 164.272.

ESI-MS: C_9_H_11_ClN_4_S 242.73 (calcd), *m*/*z* = 242.95 [M] (found).

HRMS: C_9_H_11_ClN_4_S 242.7284 (calc.), *m*/*z* = 243.0475 [M + H]^+^ (found).

LC-ESI-MS: C_9_H_11_ClN_4_S 242.73 (calcd), *m*/*z* = 243.00 [M] (found).

#### Synthesis of *tert*-Butyl 2-[(6-Chloro-2-methyl-imidazo­[1,2-*b*]­pyridazin-8-yl)­amino]­ethylsul-fanylformate **8**

237.2 mg (0.97 mmol; 1.00 equiv) of 2-[(6-chloro-2-methyl-imidazo­[1,2-*b*]­pyridazin-8-yl)­amino]­ethanethiol was dissolved in 20 mL of MeOH, and 259.7 mg (1.17 mmol; 1.20 equiv) of Boc_2_O and 0.27 mL (1.95 mmol; 2.00 equiv) of TEA were added, and the solution was stirred at RT overnight. After removing all volatiles under reduced pressure, the product remained as 286.9 mg of a beige solid with a yield of 77.24%.

^1^H NMR (400 MHz, DMSO-*d*_6_): δ 1.379 (s, H_A_, 9 H); 2.345 (s, H_B_, 3 H); 3.250 (s, H_C+D_, 4 H); 7.153 (t, ^3^ *J* = 5.9 Hz, H_X_, 1 H); 7.219 (s, H_Y_, 1 H); 8.006 (s, H_Z_, 1 H).

^13^C NMR (400 MHz, DMSO-*d*_6_): δ 8.973; 14.342; 28.170; 29.205; 38.581; 45.657; 78.003; 110.260; 114.926; 134.759; 140.881; 142.472; 144.947; 155.632.

ESI-MS: C_14_H_19_ClN_4_O_2_S 342.84 (calcd), *m*/*z* = 343.03 [M] (found).

HRMS: C_14_H_19_ClN_4_O_2_S 342.8443 (calcd), *m*/*z* = 343.1006 [M] (found).

LC-ESI-MS: C_14_H_19_ClN_4_O_2_S 342.84 (calcd), *m*/*z* = 343.10 [M] (found).

#### Synthesis of 2-Chloro-7-fluoro-4*H*-pyrido­[1,2-*a*]­pyrimidin-4-one **9**

875.7 mg (7.81 mmol; 1.00 equiv) and 4.49 mL (39.06 mmol; 5.00 equiv) of dimethylmalonate were heated to 230 °C for 50 min until dryness. The solid formed (7-fluoro-2-hydroxy-pyrido­[1,2-*a*]­pyrimidin-4-one **12**) was suspended in 1.36 mL (7.81 mmol; 1.00 equiv) of DIPEA and 3.65 mL (39.06 mmol; 5.00 equiv) of POCl_3_ and heated to 130 °C overnight. After cooling the solution, EtOH was added, and the solution was diluted with acetone, adsorbed on Celite, and purified using column chromatography with 1:1 to 7:3 acetone/CH and contaminated fractions using RP FCC. The product was recovered as 717.1 mg of a red solid with a yield of 46.24%.

**12**: ^1^H NMR (400 MHz, DMSO-*d*_6_): δ 5.192 (s, 1H); 7.481–7.505 (m, 1H); 8.143–8.160 (m, 1H); 8.889 (s, 1 H); 11.985 (s, 1H).

ESI-MS: C_8_H_5_FN_2_O_2_ 180.14 (calc.), *m*/*z* = 181.05 [M + H]^+^ (found).

HRMS: C_8_H_5_FN_2_O_2_ 180.1359 (calcd), *m*/*z* = 218.2106 [M + K] (found).

LC-ESI-MS: C_8_H_5_FN_2_O_2_ 180.14 (calc.), *m*/*z* = 181.05 [M + H]^+^ (found).

**9**: ^1^H NMR (300 MHz, DMSO-*d*_6_): δ 6.550 (s, H_A_, 1 H); 7.869 (ddd, ^3^ *J* = 9.6 Hz, ^4^ *J* = 5.3 Hz, ^5^ *J* = 0.6 Hz, H_B_, 1H); 8.211–8.277 (m, H_C_, 1 H); 8.994 (ddt, ^3^ *J* = 4.7 Hz, ^4^ *J* = 2.9 Hz, ^5^ *J* = 0.4 Hz, H_D_, 1H).

^13^C NMR (400 MHz, DMSO-*d*_6_): δ 100.882; 114.483; 127.713; 131.510; 148.418; 153.241; 155.662; 156.709.

^19^F-NMR (400 MHz, DMSO-*d*_6_): δ −131.590 – (−131.545) (m, 1 F).

ESI-MS: C_8_H_4_ClFN_2_O 198.58 (calcd), *m*/*z* = 199.03 [M] (found).

HRMS: C_8_H_4_ClFN_2_O 198.5816 (calc.), *m*/*z* = 274.2743 [M + 2K + H]^+^ (found).

LC-ESI-MS: C_8_H_4_ClFN_2_O 198.58 (calcd), *m*/*z* = 199.00 [M] (found).

#### Synthesis of 7-Fluoro-2-(2-methylimidazo­[1,2-*b*]­pyridazin-6-yl)­pyrido­[1,2-*a*]­pyrimidin-4-one **15**

99.5 mg (0.60 mmol; 1.20 equiv) of 6-chloro-2-methyl-imidazo­[1,2-*b*]­pyridazine, 152.7 mg (0.60 mmol; 1.20 equiv) of B_2_pin_2_, 117.2 mg (1.20 mmol; 2.40 equiv) of KOAc, and 49.0 mg (0.06 mmol; 0.10 equiv) of Pd­(dppf)_2_Cl_2_·DCM were suspended in 5 mL of dioxane and heated at 90 °C for 3 h under nitrogen protection. The solution was then diluted with ethyl acetate (EA), filtered through Celite, and washed with EA, and all volatile components were removed under reduced pressure. The intermediate product obtained was combined with 99.6 mg (0.50 mmol; 1.00 equiv) of 2-chloro-7-fluoro-4*H*-pyrido­[1,2-*a*]­pyrimidin-4-one and 31.9 mg (0.03 mmol; 0.06 equiv) of Pd­(P­(Ph)_3_)_4_ and suspended in 12 mL of ACN, and further 169.1 mg (1.20 mmol; 2.40 equiv) of K_2_CO_3_ in 3 mL of water were added. The suspension was heated to 90 °C overnight under nitrogen protection. The precipitate was filtered, washed with 20 mL each of ether and water, and dried. The product was obtained in quantitative yield as a brown-black solid.

HRMS: C_15_H_10_FN_5_O 295.2712 (calc.), *m*/*z* = 296.0947 [M + H]^+^ (found).

LC-ESI-MS: C_15_H_10_FN_5_O 295.27 (calc.), *m*/*z* = 296.10 [M + H]^+^ (found).

#### Synthesis of 2-(2-Methylimidazo­[1,2-*b*]­pyridazin-6-yl)-7-piperazin-1-yl-pyrido­[1,2-*a*]­pyrimidin-4-one **17**

78.6 mg (0.27 mmol; 1.00 equiv) of 7-fluoro-2-(2-methylimidazo­[1,2-*b*]­pyridazin-6-yl)­pyrido­[1,2-*a*]­pyrimidine-4-one and 177.1 mg (0.95 mmol; 3.50 equiv) of *tert*-butyl piperazine-1-carboxylate were heated in 2 mL of analytical grade DMSO at 125 °C for 24 h under nitrogen protection. Purification was carried out using RP FCC. The product was recovered as 43.6 mg of a yellow solid with a yield of 34.99%. For deprotection, the product was dissolved in DCM and TFA, and all volatile components were removed after stirring at RT for at least 1 h.

##### Protected

HRMS: C_24_H_27_N_7_O_3_ 461.5163 (calc.), *m*/*z* = 462.2265 [M + H]^+^ (found).

LC-ESI-MS: C_24_H_27_N_7_O_3_ 461.52 (calc.), *m*/*z* = 462.15 [M + H]^+^ (found).

##### Deprotected

HRMS: C_19_H_19_N_7_O 361.4005 (calc.), *m*/*z* = 362.1721 [M + H]^+^ (found).

LC-ESI-MS: C_19_H_19_N_7_O 361.40 (calc.), *m*/*z* = 362.10 [M + H]^+^ (found).

^1^H NMR (300 MHz, DMSO-*d*_6_): δ 2.439 (s, H_A_, 3H); 3.000 (s, H_B_, 4H); 3.273 (s, H_C_, 4H); 7.106 (s, H_T_, 1H); 7.842 (d, ^3^ *J* = 9.9 Hz, H_U_, 1H); 8.083–8.190 (m, H_Arom_, 5H).

#### Synthesis of 3-[2,3,5,6-Tetrafluoro-4-(2,3,4,5,6-pentafluorophenyl)­phenyl]­sulfanylpropanoic Acid **18**

1116.5 mg (3.33 mmol; 1.00 equiv) of decafluoro-biphenyl and 353.4 mg (3.33 mmol; 1.00 equiv) of 3-mercaptopropanoic acid were dissolved in 20 mL of ACN, and 0.93 mL (6.66 mmol; 2.00 equiv) of TEA was dissolved and stirred at RT for 1 h. The solution was coevaporated with Celite and purified using RP FCC. The product was recovered as 592.8 mg of a white solid with a yield of 42.36%.

^1^H NMR (400 MHz, DMSO-*d*_6_): δ 2.922 (t, ^3^ *J* = 6.9 Hz, H_A_, 2H); 3.233 (t, ^3^ *J* = 6.8 Hz, H_B_, 2H); 12.427 (s, H_C_, 2H).

^19^F-NMR (300 MHz, DMSO-*d*_6_): δ −160.872–(−160.716) (m, 2F); −149.773 (t, *J* = 22.4 Hz, 1 F); −138.747–(−138.306) (m, 4F); −133.149–(−132.960) (m, 2F).

ESI-MS: C_15_H_5_F_9_O_2_S 420.25 (calcd), *m*/*z* = 418.79 [M-H]^−^ (found).

MALDI-MS: C_15_H_5_F_9_O_2_S 420.24963 (calc.), *m*/*z* = 421.33981 [M-H]^−^ (found).

#### Synthesis of 2-(2-Methylimidazo­[1,2-*b*]­pyridazin-6-yl)-7-[4-[3-[2,3,5,6-tetrafluoro-4-(2,3,4,5,6-pentafluorophenyl)­phenyl]­sulfanylpropanoyl]­piperazin-1-yl]­pyrido­[1,2-*a*]­pyrimidin-4-one **20**

19.3 mg (0.05 mmol; 1.00 equiv) of 2-(2-Methylimidazo­[1,2-*b*]­pyridazin-6-yl)-7-piperazin-1-yl-pyrido­[1,2-*a* ]­pyrimidin-4-one, 23.7 mg (0.05 mmol, 1.00 equiv) of 3-[2,3,5,6-tetrafluoro-4-(2,3,4,5,6-pentafluorophenyl)­phenyl]­sulfanylpropanoic acid, and 26.4 mg (0.07 mmol; 1.20 equiv) of HATU were dissolved in 0.8 mL of DMF, 2 mL of ACN, and 0.01 mL (0.12 mmol; 2.00 equiv) of TEA. After stirring at RT for 4 days and under nitrogen protection, purification was carried out using RP FCC. The product was recovered as 4.8 mg of a yellow solid with a yield of 12.57%.

^1^H NMR (400 MHz, DMSO-*d*_6_): δ 2.437 (s, H_A_, 3H); 2.812 (t, ^3^ *J* = 6.6 Hz, H_B_, 2H); 2.866 (t, ^3^ *J* = 6.4 Hz, H_C_, 2H); 3.247–3.279 (m, H_D_, 2H); 3.453 (s, H_E_, 2H); 3.649 (t, ^3^ *J* = 4.9 Hz, H_F_, 4H); 7.100 (s, H_T_, 1H); 7.529–7.599 (m, H_U_, 1H); 7.848 (d, ^3^ *J* = 9.7 Hz, H_V_, 1H); 8.136 (d, ^3^ *J* = 11.5 Hz, H_W_, 1H); 8.160–8.190 (m, H_X+Y_, 2H); 8.322 (d, ^4^ *J* = 2.4 H_Z_, 1H).

^13^C NMR (500 MHz, DMSO-*d*_6_): 15.138; 28.620; 30.068; 30.593; 31.159; 33.792; 33.844; 35.192; 41.104; 42.297; 44.522; 44.741; 44.932; 45.317; 45.863; 48.071; 48.313; 97.499; 109.411; 115.017; 115.682; 124.834; 127.133; 132.878; 138.592; 142.378; 147.267; 148.718; 155.317; 157.353; 161.624; 169.266.

^19^F-NMR (400 MHz, DMSO-*d*_6_): δ −160.806–(−160.637) (m, 2F); −149.755 (t, *J* = 22.1 Hz, 1F); −138.804–(−138.707) (m, 2F); −138.437–(−138.346) (m, 2F); −133.074–(−133.002) (m, 2F).

HRMS: C_34_H_22_F_9_N_7_O_2_S 763.6348 (calc.), *m*/*z* = 764.1497 [M + H]^+^ (found).

LC-ESI-MS: C_34_H_22_F_9_N_7_O_2_S 763.63 (calc.), *m*/*z* = 764.15 [M + H]^+^ (found).

#### Synthesis of 2-[2,3,5,6-Tetrafluoro-4-(2,3,4,5,6-pentafluorophenyl)­phenyl]­sulfanylethanamine **21**

215.9 mg (0.65 mmol; 1.00 equiv) of decafluoro-biphenyl and 73.8 mg (0.65 mmol; 1.00 equiv) of cysteamine hydrochloride were dissolved in 20 mL of ACN, and 0.18 mL (1.30 mmol; 1.00 equiv) of TEA was dissolved and stirred at RT for 1 h. The precipitate was dissolved in MeOH, adsorbed on Celite, and purified using RP FCC. The product was recovered as 125.7 mg of a white solid with a yield of 49.43%.

^1^H NMR (300 MHz, DMSO-*d*_6_): δ 2.927 (s, H_A_, 2H); 3.204 (s, H_B_, 2H); 8.244 (s, H_C_, 2H).

^19^F-NMR (300 MHz, DMSO-*d*_6_): δ −160.772–(−160.635) (m, 2F); −149.617 (t, *J* = 22.8 Hz, 1F); −138.472–(−138.362) (m, 4F); −132.743–(−132.665) (m, 2F).

ESI-MS: C_14_H_6_F_9_NS 391.25 (calc.), *m*/*z* = 391.82 [M + H]^+^ (found).

MALDI-MS: C_14_H_6_F_9_NS 391.25477 (calc.), *m*/*z* = 392.01505 [M + H]^+^ (found).

LC-ESI-MS: C_14_H_6_F_9_NS 391.25 (calc.), *m*/*z* = 391.95 [M + H]^+^ (found).

#### Synthesis of 4-Oxo-4-[2-[2,3,5,6-tetrafluoro-4-(2,3,4,5,6-pentafluorophenyl)­phenyl]­sulfanylethylamino]­butanoic Acid **23a**

49.0 mg (0.28 mmol; 1.00 equiv) of 4*-tert*-butoxy-4-oxobutanoic acid, 110.4 mg (0.28 mmol, 1.00 equiv) of 2-[2,3,5,6-tetrafluoro-4-(2,3,4,5,6-pentafluorophenyl)­phenyl]­sulfanylethanamine, and 130.8 mg (0.34 mmol; 1.20 equiv) of HATU were dissolved in 0.5 mL of DMF and 0.08 mL (0.56 mmol; 2.00 equiv) TEA. After stirring at RT for 3 days under nitrogen protection, purification was carried out using RP FCC. The intermediate product was dissolved in TFA and DCM and, after stirring for 1 h at RT, purified again using RP FCC. The product was recovered as 45.6 mg of a white solid with a yield of 33.14%.

##### Protected

^1^H NMR (400 MHz, DMSO-*d*_6_): δ 1.348 (s, H_A_, 9H); 2.215 (t, ^3^ *J* = 6.9 Hz, H_B_, 2H); 2.349 (t, ^3^ *J* = 7.2 Hz, H_C_, 2H); 3.161 (t, ^3^ *J* = 6.4 Hz, H_D_, 2H); 3.296 (q, ^3^ *J* = 6.6 Hz, H_E_, 2H); 8.037 (s, H_Z_, 1H).

^19^F-NMR (400 MHz, DMSO-*d*_6_): δ −160.810-(−160.669) (m, 2F); −149.743 (t, *J* = 22.7 Hz, 1F); −138.719–(−138.659) (m, 2F); −138.376–(−138.330) (m, 2F); −132.902 (q, *J* = 11.5 Hz, 2F).

HRMS: C_22_H_18_F_9_NO_3_S 547.4338 (calc.), *m*/*z* = 548.0939 [M + H]^+^ (found).

LC-ESI-MS: C_22_H_18_F_9_NO_3_S 547.43 (calc.), *m*/*z* = 570.05 [M + Na]^+^ (found).

##### Deprotected

^1^H NMR (300 MHz, DMSO-*d*_6_): δ 2.253 (t, ^3^ *J* = 6.5 Hz, H_A_, 2H); 2.395 (t, ^3^ *J* = 7.1 Hz, H_B_, 2H); 3.171 (t, ^3^ *J* = 6.2 Hz, H_C_, 2H); 3.288 (q, ^3^ *J* = 6.0 Hz, H_D_, 2H); 8.053 (d, ^3^ *J* = 5.3 Hz, H_Y_, 1H); 12.030 (s, H_Z_, 1H).

^13^C NMR (300 MHz, DMSO-*d*_6_): δ 29.457; 30.294; 31.158; 33.641; 101.900; 105.357; 117.710; 137.237; 139.192; 143.038; 143.443; 145.026; 145.462; 146.182; 148.137; 171.639; 174.159.

^19^F-NMR (300 MHz, DMSO-*d*_6_): δ −160.848–(−160.639) (m, 2F); −149.736 (tt, *J* = 22.4 Hz, *J* = 3.0 Hz, 1F); −138.778–(−138.573) (m, 2F); −138.428-(−138.258) (m, 2 F); −132.983–(−132.842) (m, 2F).

HRMS: C_18_H_10_F_9_NO_3_S 491.3275 (calc.), *m*/*z* = 492.0323 [M + H]^+^ (found).

LC-ESI-MS: C_18_H_10_F_9_NO_3_S 491.33 (calc.), *m*/*z* = 492.01 [M + H]^+^ (found).

#### Synthesis of 4-[4-[2-(2-Methylimidazo­[1,2-*b*]­pyridazin-6-yl)-4-oxo-pyrido­[1,2-*a*]­pyrimidin-7-yl]­piperazin-1-yl]-4-oxo-*N*-[2-[2,3,5,6-tetrafluoro-4-(2,3,4,5,6-pentafluorophenyl)­phenyl]­sul-fanylethyl]­butanamide **24a**

22.8 mg (0.06 mmol; 1.00 equiv) of 2-(2-methylimidazo­[1,2-*b*]­pyridazin-6-yl)-7-piperazin-1-yl-pyrido­[1,2-*a* ]­pyrimidin-4-one, 29.4 mg (0.06 mmol, 1.00 equiv) of 4-oxo-4-[2-[2,3,5,6-tetrafluoro-4-(2,3,4,5,6-pentafluorophenyl)­phenyl]­sulfanylethylamino]­butanoic acid, and 30.7 mg (0.08 mmol; 1.20 equiv) of HATU were dissolved in 0.5 mL of DMF, 2 mL of ACN, and 0.02 mL (0.12 mmol; 2.00 equiv) of TEA. After stirring at RT overnight under nitrogen protection, purification was carried out using RP FCC. The product was recovered as 18.6 mg of a yellow solid with a yield of 37.14%.

^1^H NMR (500 MHz, DMSO-*d*_6_): δ 2.312 (t, ^3^ *J* = 6.8 Hz, H_A_, 2H); 2.439 (s, H_B_, 3H); 2.596 (t, ^3^ *J* = 7.3 Hz, H_C_, 2H); 3.171 (t, *J* = 6.5 Hz, H_D+E_, 4H); 3.654 (s, H_F+G_, 8H); 7.095 (s, H_S_, 1H); 7.840 (d, ^3^ *J* = 10.1 Hz, H_T_, 1H); 8.064 (t, ^3^ *J* = 5.5 Hz, H_U_, 1H); 8.108 (d, ^3^ *J =* 9.6 Hz, H_V_, 1H); 8.140 (d, ^3^ *J* = 4.2 Hz, H_W_, 1H); 8.158–8.169 (m, H_X_, 1H); 8.189 (s, H_Y_, 1H); 8.320 (d, ^4^ *J* = 2.6 H_Z_, H_Z_, 1H).

^13^C NMR (500 MHz, DMSO-*d*_6_): 15.143; 28.108; 29.458; 30.294; 30.692; 31.160; 31.241; 33.721; 36.250; 41.046; 43.358; 44.591; 48.075; 48.313; 97.451; 109.328; 115.015; 115.668; 117.727; 124.834; 127.110; 132.813; 138.594; 142.423; 146.150; 147.228; 148.716; 155.293; 157.344; 163.494; 170.455; 172.103.

^19^F-NMR (500 MHz, DMSO-*d*_6_): δ −160.791-(−160.688) (m, 2 F); −149.770 (t, *J* = 22.3 Hz, 1F); −138.715–(−138.645) (m, 2F); −138.355–(−138.305) (m, 2F); −132.955–(−132.882) (m, 2F).

HRMS: C_37_H_27_F_9_N_8_O_3_S 834.7127 (calc.), *m*/*z* = 835.1874 [M + H]^+^ (found).

LC-ESI-MS: C_37_H_27_F_9_N_8_O_3_S 834.71 (calcd), *m*/*z* = 835.20 [M + H]^+^ (found).

#### Synthesis of 6-Oxo-6-[2-[2,3,5,6-tetrafluoro-4-(2,3,4,5,6-pentafluorophenyl)­phenyl]­sulfanylethylamino]­hexanoic Acid **23b**

114.6 mg (0.57 mmol; 1.20 equiv) of 6-*tert*-butoxy-6-hexanoic acid, 183.4 mg (0.47 mmol, 1.00 equiv) of 2-[2,3,5,6-tetrafluoro-4-(2,3,4,5,6-pentafluorophenyl)­phenyl]­sulfanylethanamine, and 212.7 mg (0.56 mmol; 1.20 equiv) of HATU were dissolved in 0.5 mL of DMF and 0.13 mL (0.94 mmol; 2.00 equiv) of TEA. After stirring at RT overnight under nitrogen protection, purification was carried out using RP FCC. The intermediate product was dissolved in TFA and DCM, and after stirring at RT for 1 h, all volatile components were removed under reduced pressure. The product was recovered as 89.8 mg of a white solid with a yield of 36.79%.

##### Protected

^1^H NMR (300 MHz, DMSO-*d*_6_): δ 1.373 (s, H_A_, 9H); 1.457 (quint, ^3^ *J* = 3.1 Hz, H_B+C_, 4H); 1.978 (t, ^3^ *J* = 6.2 Hz, H_D_, H); 2.170 (t, ^3^ *J* = 6.7 Hz, H_E_, 2H); 3.185 (t, ^3^ *J* = 6.0 Hz, H_F_, 2H); 3.271 (q, ^3^ *J* = 5.7 Hz, H_G_, 1H); 7.978 (t, ^3^ *J* = 5.2 Hz, H_Z_, 1H).

^19^F-NMR (300 MHz, DMSO-*d*_6_): δ −160.885–(−160.656) (m, 2F); −149.762 (tt, *J* = 22.3 Hz, *J* = 3.1 Hz, 1F); −138.851–(−138.647) (m, 2F); −138.532–(−138.363) (m, 2 F); −132.989–(−132.848) (m, 2F).

HRMS: C_24_H_22_F_9_NO_3_S 575.4870 (calcd), *m*/*z* = 576.1251 [M + H]^+^ (found).

LC-ESI-MS: C_24_H_22_F_9_NO_3_S 575.49 (calc.), *m*/*z* = 598.10 [M + Na]^+^ (found).

##### Deprotected

^1^H NMR (300 MHz, DMSO-*d*_6_): δ 1.469 (quint, ^3^ *J* = 3.7 Hz, H_A+B_, 4H); 2.004 (t, ^3^ *J* = 6.6 Hz, H_C_, 2H); 2.170 (t, ^3^ *J* = 6.4 Hz, H_D_, 2H); 3.183 (t, ^3^ *J* = 6.6 Hz, H_E_, 2H); 3.309 (q, ^3^ *J* = 6.6 Hz, H_F_, 1H); 5.322 (s, H_Y_, 1H); 7.983 (t, ^3^ *J* = 5.6 Hz, H_Z_, 1H).

^13^C NMR (400 MHz, DMSO-*d*_6_): δ 17.204; 18.560; 24.551; 25.107; 31.160; 33.686; 33.831; 35.348; 54.071; 117.756; 172.564; 174.774.

^19^F-NMR (300 MHz, DMSO-*d*_6_): δ −160.844–(−160.636) (m, 2F); −149.742 (tt, *J* = 22.4 Hz, *J* = 2.7 Hz, 1F); −138.838–(−138.633) (m, 2F); −138.523–(−138.336) (m, 2F); −132.983–(−132.843) (m, 2F).

HRMS: C_20_H_14_F_9_NO_3_S 519.3807 (calcd), *m*/*z* = 520.0636 [M + H]^+^ (found).

LC-ESI-MS: C_20_H_14_F_9_NO_3_S 519.38 (calc.), *m*/*z* = 520.05 [M + H]^+^ (found).

#### Synthesis of 6-[4-[2-(2-Methylimidazo­[1,2-*b*]­pyridazin-6-yl)-4-oxo-pyrido­[1,2-*a*]­pyrimidin-7-yl]­piperazin-1-yl]-6-oxo-*N*-[2-[2,3,5,6-tetrafluoro-4-(2,3,4,5,6-pentafluorophenyl)­phenyl]­sulfa-nylethyl]­hexanamide **24b**

18.2 mg (0.05 mmol; 1.00 equiv) of 2-(2-methylimidazo­[1,2-*b*]­pyridazin-6-yl)-7-piperazin-1-yl-pyrido­[1,2-*a* ]­pyrimidin-4-one, 26.2 mg (0.05 mmol, 1.00 equiv) of 6-oxo-6-[2-[2,3,5,6-tetrafluoro-4-(2,3,4,5,6-pentafluorophenyl)­phenyl]­sulfanylethylamino]­hexanoic acid, and 23.3 mg (0.06 mmol, 1.20 equiv) of HATU were dissolved in 1.1 mL of DMF, 2 mL of ACN, and 0.01 mL (0.10 mmol; 2.00 equiv) of TEA. After stirring at RT overnight under nitrogen protection, purification was carried out using RP FCC. The product was recovered as 15.5 mg of a yellow solid with a yield of 35.93%.

^1^H NMR (400 MHz, DMSO-*d*_6_): δ 1.497 (quint, ^3^ *J* = 6.7 Hz, H_A+B_, 4H); 2.027 (t, ^3^ *J* = 6.7 Hz, H_C_, 2H); 2.366 (t, ^3^ *J* = 6.6 Hz, H_D_, 2H); 2.456 (s, H_E_, 3H); 3.181 (t, ^3^ *J* = 6.3 Hz, H_F_, 2H); 3.261–3.291 (m, H_G+H_, 6H); 3.653 (t, ^3^ *J* = 4.9 Hz, H_I_, 4H); 7.093 (s, H_S_, 1H); 7.840 (d, ^3^ *J* = 9.6 Hz, H_T_, 1H); 8.006 (t, ^3^ *J* = 5.2 Hz, H_U_, 1H); 8.131 (s, H_V_, 1H); 8.168 (d, ^3^ *J* = 9.4 Hz, H_W_, 1H); 8.218 (d, ^3^ *J* = 9.4 Hz, H_X_, 1H); 8.240 (s, H_Y_, 1H); 8.320 (d, ^4^ *J* = 2.4 H_Z_, H_Z_, 1H).

^13^C NMR (500 MHz, DMSO-*d*_6_): 14.685; 24.848; 25.368; 31.160; 32.447; 33.783; 35.527; 38.717; 40.912; 44.742; 48.157; 48.425; 97.513; 109.370; 115.224; 124.550; 127.098; 132.848; 138.288; 142.475; 147.218; 148.100; 155.046; 157.319; 163.493; 171.124; 172.700.

^19^F-NMR (400 MHz, DMSO-*d*_6_): δ −160.797–(−160.641) (m, 2F); −149.759 (t, *J* = 22.3 Hz, 1F); −138.778–(−138.640) (m, 2F); −138.423–(−138.340) (m, 2F); −132.952–(−132.856) (m, 2 F). HRMS: C_39_H_31_F_9_N_8_O_3_S 862.7659 (calc.), *m*/*z* = 863.2181 [M + H]^+^ (found).

LC-ESI-MS: C_39_H_31_F_9_N_8_O_3_S 862.77 (calc.), *m*/*z* = 863.20 [M + H]^+^ (found).

#### Synthesis of 12-Oxo-12-[2-[2,3,5,6-tetrafluoro-4-(2,3,4,5,6-pentafluorophenyl)­phenyl]­sulfanylethylamino]­dodecanoic acid **23c**

112.0 mg (0.38 mmol; 1.00 equiv) of 12-*tert*-butoxy-12-oxododecanoic acid, 150.0 mg (0.38 mmol, 1.00 equiv) of 2-[2,3,5,6-tetrafluoro-4-(2,3,4,5,6-pentafluorophenyl)­phenyl]­sulfanylethanamine, and 174.1 mg (0.46 mmol; 1.20 equiv) of HATU were dissolved in 1.0 mL of DMF and 0.11 mL (0.76 mmol; 2.00 equiv) of TEA. After stirring at RT for 5 days under nitrogen protection, purification was carried out using RP FCC. The intermediate product was dissolved in TFA and DCM, and after stirring at RT for 1 h, purified again using RP FCC. The product was recovered as 21.0 mg of a white resin with a yield of 9.24%.

##### Protected

^19^F-NMR (300 MHz, DMSO-*d*_6_): δ −160.834-(−160.663) (m, 2F); −149.735 (tt, *J* = 22.5 Hz, *J* = 2.6 Hz, 1F); −138.839-(−138.578) (m, 2F); −138.473–(−138.339) (m, 2F); −132.991–(−132.846) (m, 2F).

HRMS: C_26_H_26_F_9_NO_3_S 659.6465 (calc.), *m*/*z* = 660.2449 [M + H]^+^ (found).

##### Deprotected

^1^H NMR (400 MHz, DMSO-*d*_6_): δ 1.213 (s, H_A‑F_, 12 H); 1.426–1.462 (m, H_G+H_, 4 H); 1.984 (t, ^3^ *J* = 7.6 Hz, H_I_, 2H); 2.189 (t, ^3^ *J* = 7.2 Hz, H_J_, 2H); 3.189 (t, ^3^ *J* = 5.7 Hz, H_K_, 2H); 3.289 (q, ^3^ *J* = 5.7 Hz, H_L_, 1 H); 7.946 (t, ^3^ *J* = 5.0 Hz, H_Y_, 1H); 11.924 (s, H_Z_, 1H).

^19^F-NMR (300 MHz, DMSO-*d*_6_): δ −160.841-(−160.633) (m, 2F); −149.690 (tt, *J* = 22.7 Hz, *J* = 2.7 Hz, 1F); −138.874-(−138.670) (m, 2F); −138.505-(−138.336) (m, 2F); −132.999-(−132.859) (m, 2F).

HRMS: C_26_H_26_F_9_NO_3_S 603.5402 (calc.), *m*/*z* = 604.1576 [M + H]^+^ (found).

LC-ESI-MS: C_26_H_26_F_9_NO_3_S 603.54 (calc.), *m*/*z* = 604.20 [M + H]^+^ (found).

#### Synthesis of 12-[4-[2-(2-Methylimidazo­[1,2-*b*]­pyridazin-6-yl)-4-oxo-pyrido­[1,2-*a*]­pyrimidin-7-yl]­piperazin-1-yl]-12-oxo-*N*-[2-[2,3,5,6-tetrafluoro-4-(2,3,4,5,6-pentafluorophenyl)­phenyl]-sulfa-nylethyl]­dodecanamide **24c**

11.9 mg (0.03 mmol; 1.00 equiv) of 2-(2-methylimidazo­[1,2-*b*]­pyridazin-6-yl)-7-piperazin-1-yl-pyrido­[1,2-*a* ]­pyrimidin-4-one, 19.5 mg (0.03 mmol, 1.00 equiv) of 12-oxo-12-[2-[2,3,5,6-tetrafluoro-4-(2,3,4,5,6-pentafluorophenyl)­phenyl]­sulfanylethylamino]­dodecanoic acid, and 15.9 mg (0.04 mmol, 1.20 equiv) of HATU were dissolved in 0.6 mL of DMF, 2 mL of ACN, and 0.01 mL (0.06 mmol; 2.00 equiv) of TEA. After stirring at RT overnight under nitrogen protection, purification was carried out using RP FCC. The product was recovered as 2.2 mg of a yellow solid with a yield of 7.75%.

^1^H NMR (500 MHz, DMSO-*d*_6_): δ 1.220–1.272 (m, H_A‑F_, 12H); 1.454 (quint, ^3^ *J* = 7.1 Hz, H_G_, 2H); 1.516 (quint, ^3^ *J* = 6.6 Hz, H_H_, 2H); 1.973 (t, ^3^ *J* = 7.5 Hz, H_I_, 2H); 2.357–2.370 (m, H_J_, 2H); 2.442 (s, H_K_, 3 H); 3.177 (t, ^3^ *J* = 6.3 Hz, H_L_, 2H); 3.257–3.295 (m, H_M+N_, 6H); 3.664 (t, ^3^ *J* = 4.5 Hz, H_O_, 4H); 7.108 (s, H_S_, 1H); 7.850 (d, ^3^ *J* = 9.6 Hz, H_T_, 1H); 7.943 (t, ^3^ *J* = 5.3 Hz, H_U_, 1 H); 8.103–8.200 (m, H_V–Y_, 4H); 8.335 (d, ^4^ *J* = 2.6 H_Z_, H_Z_, 1H).

^13^C NMR (500 MHz, DMSO-*d*_6_): δ 9.106; 15.089; 25.225; 25.565; 29.071; 29.211; 29.251; 29.352; 31.162; 32.676; 33.741; 35.664; 46.233; 48.455; 54.068; 97.488; 109.388; 115.045; 124.819; 127.123; 132.861; 142.464; 171.275; 172.793; 206.947.

^19^F-NMR (500 MHz, DMSO-*d*_6_): δ −160.783–(−160.681) (m, 2 F); −149.730 (t, *J* = 22.3 Hz, 1 F); −138.796–(−138.726) (m, 2 F); −138.427–(−138.360) (m, 2 F); −132.972–(−132.900) (m, 2 F).

HRMS: C_45_H_43_F_9_N_8_O_3_S 946.9253 (calc.), *m*/*z* = 947.3108 [M + H]^+^ (found).

LC-ESI-MS: C_45_H_43_F_9_N_8_O_3_S 946.93 (calc.), *m*/*z* = 947.30 [M + H]^+^ (found).

## Biology

### Construction of Cas13s and Related gRNAs Expression Plasmids

Catalytically inactive Cas13 variants (dRfxCas13d, dRanCas13b, dLwaCas13a, and dPspCas13b) were obtained in pHAGE backbones from Addgene (pHAGE-IRES-puro-NLS-dRfxCas13d-EGFP-NLS-3 × FLAG, #132411; pHAGE-IRES-puro-NLS-dRanCas13b-EGFP-NLS-3 × FLAG, #132409; pHAGE-IRES-puro-NLS-dLwaCas13a-EGFP-NLS-3 × FLAG, #132407; pHAGE-IRES-puro-NLS-dPspCas13b-EGFP-NLS-3 × FLAG, #132397). A four-amino acid π-clamp tag (FCPF; Phe–Cys–Pro–Phe) encoded by 5′-TTC TGC CCA TTC-3′ was inserted at the C-terminus of each construct (GenScript, custom gene synthesis/subcloning). Clones were sequence-verified across the junctions by Sanger sequencing. The original plasmids pC0040-LwaCas13a (#103851), pC0041-RanCas13b (#103852), pC0043-PspCas13b crRNA backbone (#103854), and pSLQ5429_pUC_hU6-crScaffold_EF1a-BFP (#155306) were purchased from Addgene. For Cas13 crRNA expression, plasmid construction from LwaCas13a, RanCas13b, and PspCas13b, BbsI (NEB, R0539L) was used for the digestion of pC0040, pC0041, and pC0043. For Cas13 gRNA expression, plasmid construction from RfxCas13d, BbsI, and *Eco*RI (NEB, R0101S) was used for the digestion of pSLQ5429. The resulting large fragments (linearized plasmid backbone) were purified after electrophoresis with the Kit (Gel and PCR cleanup kit, Macherey-Nagel, 740609.250). The oligos (up- and low-) including crRNAs and DR sequences were synthesized from IDT (Integrated DNA Technologies) (Table SI3). Each pair of oligos (up- and low-) was annealed according to the conditions: 30 min at 37 °C, 5 min at 95 °C, then ramped down to 15 °C at 5 °C/min. The annealed oligos were ligated with the purified plasmid backbone with T4 DNA ligase (NEB, M0202S) at room temperature for 1 h. After transformation and selection with antibiotics, the plasmid minipreps from the positive clones were prepared with a plasmid extraction kit (QIAprep Spin, Plasmid Miniprep Kit, Qiagen, cat. 27106) for further confirmation.

The constructed Cas13 crRNA expression plasmids were designed in two batches. The first batch was to test the effects of the different Cas13 subtypes. The 3xcrRNA (with three different DRs) together were cloned into 4 plasmid backbones from different Cas13 subtypes (LwaCas13a, RanCas13b, PspCas13b, RfCas13d) to get the constructs: LwaCas13a-3xcrRNA-3xDR; RanCas13b-3xcrRNA-3xDR; PspCas13b-3xcrRNA-3xDR; and RfxCas13d-3xcrRNA-3xDR. The second batch was to test the effect of different crRNAs (crRNA1, 2, and 3). The different crRNAs (cRNA1, cRNA2, and crRNA3) were cloned into RfxCas13d plasmid backbone, respectively, to get the constructs: RfxCas13d-crRNA1, RfxCas13d-crRNA2, and RfxCas13d-crRNA3. All of the constructs were confirmed by Sanger sequencing in Microsyth Seqlab. The sequences used in this work are listed in Table SI3.

### Cell Culture

HEK293T (ATCC, CRL-3216) were cultured in Dulbecco’s modified Eagle’s medium (DMEM; Thermo Fisher Scientific, 31966021). The media were supplemented with 10% fetal bovine serum (FBS) and 100 mg/mL (1%) penicillin/streptomycin (PS). The cells were cultivated at 37 °C in a humidified atmosphere with 5% CO_2_.

### SMN2/dCas13 Transfection

Before transfection, plasmids encoding SMN2, dCas13, and the corresponding crRNAs were expanded in *Escherichia coli*, followed by plasmid extraction using a Plasmid Miniprep kit (Sigma-Aldrich, Cat. No. PLN350). Transfections (plasmids: SMN2, dCas13, and crRNA) were performed as previously described.^36^ HEK293T cells were seeded in 12-well plates, with each well containing 150,000 cells (60–80% confluent) and incubated overnight in DMEM supplemented with 10% FBS and 1% PS. Lipofectamine 3000 (Thermo Fisher Scientific, L3000015) was used as the transfection reagent. For the preparation of the transfection mixture, in tube I, Lipofectamine (2 μL) was added to 50 μL of Opti-MEM medium. In tube II, P3000 (2 μL) and 2500 ng of plasmid were added to another 50 μL of Opti-MEM medium. The contents of tube II were then combined with those of tube I and incubated at room temperature for 5–10 min. The cell culture medium was refreshed with 1.5 mL of DMEM containing 10% FCS, without PS, and then the transfection mixture was added to the cells. The cells were incubated overnight, followed by medium replacement with DMEM supplemented with 10% FCS and 1% PS, and further incubated for 48 h before treatment with the compounds specified in the main text. Three consecutive crRNA sequence targeting exon 7 regions of *SMN2* were delivered using pC0041-RanCas13b crRNA backbone, pC0040-LwaCas13a crRNA backbone, PC0043-PspCas13b-crRNA backbone, and pSLQ5429_pUC_hU6-crScaffold_EF1a-BFP. Single-guide RNAs (sgRNAs) targeting exon 7 regions of *SMN2* were delivered using pSLQ5429_pUC_hU6-crScaffold_EF1a-BFP.

The full DNA sequences of the crRNA expression cassettes (promoter–DR–spacer–terminator; 5′ → 3′) are provided in Table SI3. The corresponding target genomic DNA sequences (sense strand, 5′ → 3′) are as follows

*SMN2 crRNA1:* TTTTGTCTAAAACCCTGTAAGGAAAATA

*SMN2 crRNA2:* GCTGGCAGACTTACTCCTTAATTTAAGG (risdiplam-targeting site)

*SMN2 crRNA3:* CTTTCAACTTTCTAACATCTGAACTTTT

### RT-PCR

Reverse transcription-polymerase chain reaction (RT-PCR) was performed according to the manufacturer’s instructions (CFX96 Touch Real-Time PCR Detection System, Bio-Rad, Germany).^90^ Cells were washed with PBS and lysed using 200 μL of Qiazol reagent (Qiagen, Germany). To the lysate, 40 μL of chloroform was added, followed by vortexing for 15 s. The mixture was then centrifuged at 12,000*g* for 15 min at 4 °C. The aqueous (upper) phase was transferred to a new tube, mixed with 100 μL of isopropanol, and incubated for 5 min. After another centrifugation at 12,000*g* for 15 min at 4 °C, the supernatant was discarded. The RNA pellet was washed with 70% ethanol, collected by centrifugation at 7000*g* for 5 min, and then resuspended in 20 μL of RNase-free water. RNA quality was assessed using a NanoDrop.

Equivalent amounts of RNA were reverse-transcribed using a FastGene Scriptase II complementary DNA (cDNA) kit (Nippon Genetics, Germany). To each RNA sample (1 μg) were added 1 μL of hexamer and 1 μL of dT primer, followed by incubation at 42 °C for 10 min and then at room temperature for 5 min. A master mixture containing deoxynucleotide triphosphates (dNTPs), RNase inhibitor, and reverse transcriptase in reaction buffer was prepared and added to each RNA sample to a final volume of 20 μL. The mixture was incubated at 42 °C for 1 h, and the reaction was halted by heating at 80 °C for 5 min.

PCR was performed using Taq PCR Master Mix (manufacturer, catalog number). Each 20 μL reaction mixture contained 10 μL of SYBR Green PCR Master Mix, 2 μL of 1:10 diluted cDNA, 1 μL of forward and reverse primer mix (final concentration 0.5 μM each; primer sequences are listed in Table SI4), and 7 μL of nuclease-free water. β-Actin (ACTB) was used as the internal reference gene for normalization. Thermal cycling was carried out using the following conditions: initial denaturation at 95 °C for 3 min; 34 amplification cycles of 95 °C for 30 s; annealing at the gene-specific melting temperature (Tm) (*SMN2*: 49 °C; *FOXM1*: 54 °C; *APLP2*: 53 °C; *STRN3*: 53 °C; *MADD*: 53 °C) for 30 s; and extension at 68 °C for 60 s; followed by a final extension at 72 °C for 5 min. The reactions were held at 4 °C until further analysis. PCR products were visualized by electrophoresis on a 2% (w/v) agarose gel stained with diamond nucleic acid dye (Promega, Cat. No. H1181) and imaged under UV illumination.

## Supplementary Material









## Notes

Chemical characterization; functional evaluation of new Ris-PFB derivatives (Figure SI1); optimized DNA sequences for construct cloning (Table SI3); and oligonucleotides pairs (Table SI4) (PDF)
Results from AI-based ADME­(T) modeling (Table SI1) (XLSX)
SwissADME-results-v2 (Table SI2) (XLSX)
Molecular formula strings (Table SI5) (CSV)

## Abbreviations Used

AAV: adeno-associated viruses
ACN: acetonitrile
ACTB: β-actin
ADME: absorption, distribution, metabolismus and elimination
ASD: amorphous solid dispersion
B_2_Pin_2_: Bis­(pinacolato)­dibor
BBB: blood–brain barrier
Boc: *tert*-butyloxycarbonyl
bRo5: beyond-Ro5
cDNA: complementary DNA
Con_LogP: consensus LogP
crRNA: CRISPR RNA
CYP: cytochrome P
dCas13: catalytically inactive Cas13
DIPEA: diisopropylethylamine
DMPK: drug metabolism and pharmacokinetics
Dppf: (diphenylphosphin)­ferrocene
DR: direct repeat
EA: ethyl acetate
ESOL_LogS: predicted aqueous solubility
GI: gastrointestinal
HATU: *O*-(7-Azabenzotriazol-1-yl)-*N,N,N′,N*′-tetramethyluronium-hexafluorophosphate
HBA: H-bond acceptors
HBD: H-Bond Donors
iPSC: induced pluripotent stem cells
LNP: Lipid nanoparticles
MW: molecular weight
NP: normal phase
O.N.: overnight
PD: pharmacodynamics
PFB: perfluorobiphenyl
PK: pharmacokinetics
P-pg: P-glycoprotein
PPTS: *p*-toluenesulfonate
PROTAC: proteolysis targeting chimeras
qRT-PCR: quantitative reverse transcription-polymerase chain reaction
RB: rotatable bonds
Ris-PFB: risdiplam-PFB conjugate
Ro5: Rule of 5
RP FCC: reversed-phase flash column chromatography
RT: room-temperature
SMA: spinal muscular atrophy
SMN: survival motor neuron
ss: splice site
TEA: triethylamine
TFA: trifluoracetic acid
Tm: melting temperature (Tm)
TPSA: Topological Polar Surface Area
U1 snRNP: uridine-rich subunit small nuclear ribonucleoprotein 1
