# Supplementary material for: A Chemically Induced CRISPR/dCas13FCPF Platform for Precise and Programmable RNA Regulation
Source: J Med Chem. 2025 Oct 22;68(21):22633–49. doi: 10.1021/acs.jmedchem.5c01609 (PMC12621252; doi:10.1021/acs.jmedchem.5c01609)
Supplement: Supplementary file 1 [file jm5c01609_si_001.pdf]

A Chemically Induced CRISPR/dCas13^{FCPF} Platform for Precise and Programmable RNA Regulation

Sebastian. Hasselbeck,[1,2] Jianhui Wang,[1,2] Zhaodai Bai,[3,4] Tobias Hüfner[5], Gerhard Hummer[5], Phillip Grote,[3,4] Xinlai Cheng[1,2,3,6,7*]

[1]Buchmann Institute for Molecular Life Sciences, Goethe University Frankfurt am Main, Germany, 60438 Frankfurt am Main

[2]Institute of Pharmaceutical Chemistry, Goethe University Frankfurt am Main, 60438 Frankfurt am Main

[3]Frankfurt Cancer Institute, Goethe University Frankfurt am Main, 60596 Frankfurt am Main Germany

[4] Institute for Tumor Biology and Experimental Therapy, Georg-Speyer-Haus, 60596 Frankfurt am Main, Germany

[5]Max-Planck-Institute of Biophysics, 60438 Frankfurt am Main

[6]University Cancer Center (UCT) Frankfurt, 60590 Frankfurt am Main

[7]Mildred-Scheel-Nachwuchszentrum (MSNZ) Frankfurt, 60590 Frankfurt am Main

**Corresponding author: Cheng@pharmchem.uni-frankfurt.de*

Content

S3 Figure SI1: Functional Evaluation of New Ris-PFB Derivatives.

S4 Table SI3: Optimized DNA sequences for construct cloning.

S4 Table SI4: Oligonucleotides pairs.

S5 ^{1}H-, ^{13}C-NMR-, ESI-, HRMS- and LC-MS-spectrum of 8-bromo-6-chloro-2-methyl-imidazo[1,2-b]pyridazine **5**

S7 ^{1}H-, ^{13}C-NMR-, ESI-, HRMS- and LC-MS-spectrum of 2-[(6-chloro-2-methyl-imidazo[1,2-b]pyridazin-8-yl)amino]ethanethiole **7**

S10 ^{1}H-, ^{13}C-NMR-, ESI-, HRMS- and LC-MS-spectrum of 2-[(6-chloro-2-methyl-imidazo[1,2-b]pyridazin-8-yl)amino]ethanethiole **8**

S12 ^{1}H-, ^{13}C-, ^{19}F-NMR-, ESI-, HRMS- and LC-MS-spectrum of 2-Chloro-7-fluoro-4H-pyrido[1,2-a]pyrimidin-4-one **9**

S15 ^{1}H-NMR-, ESI-, HRMS- and LC-MS-spectrum of 7-Fluoro-2-hydroxy-pyrido[1,2-a]pyrimidin-4-one **12**

S17 HRMS- and LC-MS-spectrum of 7-fluoro-2-(2-methylimidazo[1,2-b]pyridazin-6-yl)pyrido[1,2-a]pyrimidin-4-one **15**

S18 HRMS- and LC-ESI-MS-spectrum of *tert*-butyl 4-[2-(2-methylimidazo[1,2-b]pyridazin-6-yl)-4-oxo-pyrido-[1,2-a]pyrimidin-7-yl]piperazine-1-carboxylate and ^{1}H-, HRMS- and LC-ESI-MS-spectrum of 2-(2-Methylimidazo[1,2-b]pyridazin-6-yl)-7-piperazin-1-yl-pyrido[1,2-a]pyrimidin-4-one **17**

S20 ^{1}H-, ^{19}F-, ESI-, MALDI- and LC-MS spectrum of 3-[2,3,5,6-tetrafluoro-4-(2,3,4,5,6-penta-fluorophenyl)phenyl]sulfanylpropanic acid **18**

S23 ^{1}H-, ^{13}C-, ^{19}F-NMR-, HRMS- and LC-ESI-MS-Spectrum of 2-(2-methylimidazo[1,2-b]pyridazin-6-yl)-7-[4-[3-[2,3,5,6-tetrafluoro-4-(2,3,4,5,6-pentafluorophenyl)phenyl]sulfanylpropanoyl]piperazin-1-yl]pyrido[1,2-a]pyrimidin-4-one **20**

S26 ^{1}H-, ^{19}F-, ESI-, MALDI-, and LC-MS-spectrum of 2-[2,3,5,6-Tetrafluoro-4-(2,3,4,5,6-pentafluoro-phenyl)phenyl]sulfanylethanamine **21**

S28 ^{1}H-, ^{19}F-NMR-, HRMS- and HPLC-spectrum of *tert*-butyl 4-oxo-4-[2-[2,3,5,6-tetrafluoro-4-(2,3,4,5,6-pentafluorophenyl)phenyl]sulfanylethylamino]butanoat and ^{1}H-, ^{13}C-, ^{19}F-NMR-, HRMS- and LC-MS-ESI-spectrum of 4-oxo-4-[2-[2,3,5,6-tetrafluoro-4-(2,3,4,5,6-pentafluorophenyl)phenyl]sulfanylethylamino]butanic acid **23 a**

S33 ^{1}H-, ^{13}C-, ^{19}F-NMR-, HRMS- and LC-ESI-MS-spectrum of 4-[4-[2-(2-Methylimidazo[1,2-b]pyridazin-6-yl)-4-oxo-pyrido[1,2-a]pyrimidin-7-yl]piperazin-1-yl]-4-oxo-*N*-[2-[2,3,5,6-tetrafluoro-4-(2,3,4,5,6-pentafluorophenyl)phenyl]sulfanylethyl]butanamide **24 a**

S35 ^{1}H-, ^{19}F-NMR-, HRMS- and LC-MS-Spectrum of *tert*-butyl 6-oxo-6-[2-[2,3,5,6-tetrafluoro-4-(2,3,4,5,6-pentafluorophenyl)phenyl]sulfanylethylamino]hexanoate and ^{1}H-, ^{13}C-, ^{19}F-NMR-, HRMS- and LC-MS-spectrum of 6-oxo-6-[2-[2,3,5,6-tetrafluoro-4-(2,3,4,5,6-pentafluorophenyl)phenyl]sulfanylethylamino]hexanic acid **23 b**

S40 ^{1}H-, ^{13}C-, ^{19}F-NMR-, HRMS- and LC-ESI-MS-spectrum of 6-[4-[2-(2-methylimidazo[1,2-b]pyridazin-6-yl)-4-oxo-pyrido[1,2-a]pyrimidin-7-yl]piperazin-1-yl]-6-oxo-*N*-[2-[2,3,5,6-tetrafluoro-4-(2,3,4,5,6-pentafluorophenyl)phenyl]sulfanylethyl]hexanamide **24 b**

S42 ^{19}F-NMR-, HPLC- and HRMS-spectrum of *tert*-butyl 12-oxo-12-[2-[2,3,5,6-tetrafluoro-4-(2,3,4,5,6-pentafluorophenyl)phenyl]sulfanylethylamino]dodecanoate and ^{1}H-, ^{19}F-NMR-, HRMS- and LC-MS-spectrum of 12-Oxo-12-[2-[2,3,5,6-tetrafluoro-4-(2,3,4,5,6-pentafluorophenyl)phenyl]sulfanylethylamino]dodecanic acid **23 c**

S45 ^{1}H-, ^{13}C-, ^{19}F-NMR- and LC-ESI-MS-spectrum of 12-[4-[2-(2-methylimidazo[1,2-b]pyridazin-6-yl)-4-oxo-pyrido[1,2-a]pyrimidin-7-yl]piperazin-1-yl]-12-oxo-*N*-[2-[2,3,5,6-tetrafluoro-4-(2,3,4,5,6-pentafluorophenyl)phenyl]sulfanylethyl]dodecanamide **24 c**

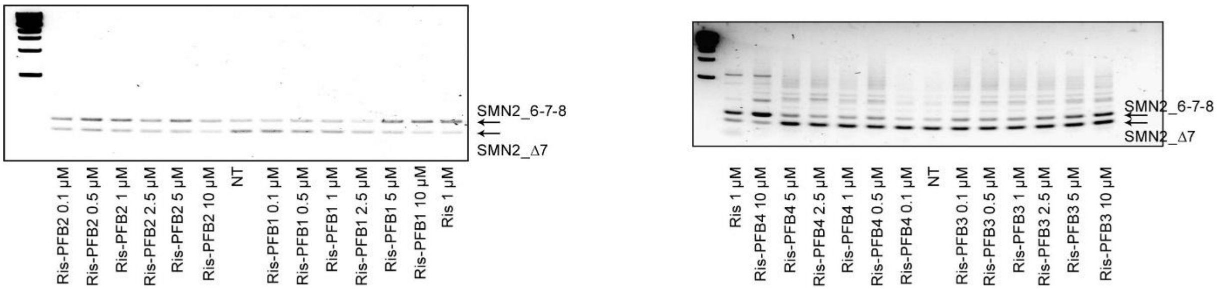

<u>Figure SI1</u>: Functional evaluation of new Ris-PFB derivatives.

Table SI3: Optimized DNA sequences for construct cloning.

**Oligos for Insert**	Sequence
**103851_crRNAs_up**	5‘-AAAAAAAAGTTCAGATGTTAGAAAGTTGAAAGGTTTTAGTCCCCTTCGTTTTTGGGGTAG-TCTAAATCCCTTAAATTAAGGAGTAAGTCTGCCAGCGTTTTAGTCCCCTTCGTTTTTGGGGTAGTCTAAATCTATTTTCCTTACAGGGTTTTAGACAAAA-3’
**103851_crRNAs_low**	5‘-AAACTTTTGTCTAAAACCCTGTAAGGAAAATAGATTTAGACTACCCCAAAAACGAAGGG-GACTAAAACGCTGGCAGACTTACTCCTTAATTTAAGGGATTTAGACTACCCCAAAAACGAAGGGGACTAAAACCTTTCAACTTTCTAACATCTGAACTTTT-3’
**103852_crRNAs_up**	5’-CACCGTTTTGTCTAAAACCCTGTAAGGAAAATAGTTGGGACTGCTCTCACTTTGAAGGG-TATTCGCTGGCAGACTTACTCCTTAATTTAAGGGTTGGGACTGCTCTCACTTTGAAGGGTATTCCTTTCAACTTTCTAACATCTGAACTTTT-3’
**103852_crRNAs_low**	5’-CAACAAAAGTTCAGATGTTAGAAAGTTGAAAGGAATACCCTTCAAAGTGAGAGCAG-TCCCAACCCTTAAATTAAGGAGTAAGTCTGCCAGCGAATACCCTTCAAAGTGAGAGCAGTCCCAACTATTTTCCTTACAGGGTTTTAGACAAAAC-3’
**103854_crRNAs_up**	5’-CACCGTTTTGTCTAAAACCCTGTAAGGAAAATAGTTGTGGAAGGTCCAGTTTTGAGGGGC-TATTACAACGCTGGCAGACTTACTCCTTAATTTAAGGGTTGTGGAAGGTCCAGTTTTGAGGGGCTATTACAACCTTTCAACTTTCTAACATCTGAACTTTT-3’
**103854_crRNAs_low**	5’-CAACAAAAGTTCAGATGTTAGAAAGTTGAAAGGTTGTAATAGCCCCTCAAAACTGGAC-CTTCCACAACCCTTAAATTAAGGAGTAAGTCTGCCAGCGTTGTAATAGCCCCTCAAAACTGGACCTTCCACAACTATTTTCCTTACAGGGTTTTAGACAAAAC-3’
**155306_crRNAs_up**	5’-AAACTTTTGTCTAAAACCCTGTAAGGAAAATACAAGTAAACCCCTAC-CAACTGGTCGGGGTTTGAAACGCTGGCAGACTTACTCCTTAATTTAAGGCAAGTAAACCCCTACCAACTGGTCGGGGTTTGAAACCTTTCAACTTTCTAACATCTGAACTTTTTTTTTTG-3’
**155306_crRNAs_low**	5’-AATTCAAAAAAAAAAAGTTCAGATGTTAGAAAGTTGAAAGGTTTCAAACCCCGACCAGTTGG-TAGGGGTTTACTTGCCTTAAATTAAGGAGTAAGTCTGCCAGCGTTTCAAACCCCGACCAGTTGGTAGGGGTTTACTTGTATTTTCCTTACAGGGTTTTAGACAAAA-3’
**155306-crRNA1-up**	AAACTTTTGTCTAAAACCCTGTAAGGAAAATATTTTTTG
**155306-crRNA1-low**	AATTCAAAAAATATTTTCCTTACAGGGTTTTAGACAAAA
**155306-crRNA2-up:**	AAACGCTGGCAGACTTACTCCTTAATTTAAGG TTTTTTG
**155306-crRNA2-low**	AATTCAAAAAACCTTAAATTAAGGAGTAAGTCTGCCAGC
**155306-crRNA3-up:**	AAACCTTTCAACTTTCTAACATCTGAACTTTTTTTTTTG
**155306-crRNA3-low**	AATTCAAAAAAAAAAGTTCAGATGTTAGAAAGTTGAAAG
SMN2 crRNA1	TTTTGTCTAAAACCCTGTAAGGAAAATA
SMN2 crRNA2	GCTGGCAGACTTACTCCTTAATTTAAGG
SMN2 crRNA3	CTTTCAACTTTCTAACATCTGAACTTTT
MADD crRNA1	TGTTCCACAATTCTACCCCAGAT
MADD crRNA2	ACTTGGGAACAGGAAAGGGCATT
MADD crRNA3	ACATGCTACCTACCTGCTGCCAT
APLP2 crRNA1	AGCCACCATATATAAAGCGCACG
APLP2 crRNA2	TTACTCATCGCTTTACACACAGC
APLP2 crRNA3	ACCACACAGATGCAGCCACGAAG
STRN3 crRNA1	GTCTACATCCCAAACCTCAGCAG
STRN3 crRNA2	ATCTGCACTTACTCTTCACCCCT
STRN3 crRNA3	CAAAGTTTAAAGTTAGTTGCCCC
FOXM1 crRNA1	ACCTATACATATTAGATTGGCAG
FOXM1 crRNA2	TGTACCAAAATCTCGCAGATCGC
FOXM1 crRNA3	AAACATAATGACAAGGTGCTCAG
FOXM1 crRNA4	AATAACCAATTCATAGAGGACAA

Table SI4: Oligonucleotides pairs.

	Forward	Reverse
SMN2_7-8 (RT-PCR)	GCTCACATTCCTTAAATTAAGGA-GAAA	TCCAGATCTGTCTGATCGTTTCTT
SMN2_6-7-8 (RT-PCR)	TGGCTATCATACTGGCTATTA-TATGGGTTT	TCCAGATCTGTCTGATCGTTTCTT
SMN2_FL (PCR)	TGGCTATCATACTGGCTATTA-TATGGAAAT	TCCAGATCTGTCTGATTGTTTCTT
FOXM1 (PCR)	GGTACCTATCCAGTTCCCGG	TATCCCCTCCTCAGCTAGCAGCAC-TGATAAA
APLP2 (PCR)	AAGAAGGGGAGGAAGTGGTG	TCATTGGTTGGCAGAGGAGT
STRN3 (PCR)	TGGTGAAGGAGCTGGAGAAG	TACCGTCAAGTCTGCAAGGT
MADD (PCR)	ACCAGACAAGCGGAAGAGAA	CCTCTGTCTCACCAAGGTCA

Chemical Characterization

^{1}H-, ^{13}C-NMR-, ESI-, HRMS- and LC-MS-spectrum of 8-bromo-6-chloro-2-methyl-imid-azo[1,2-b]pyridazine **5**

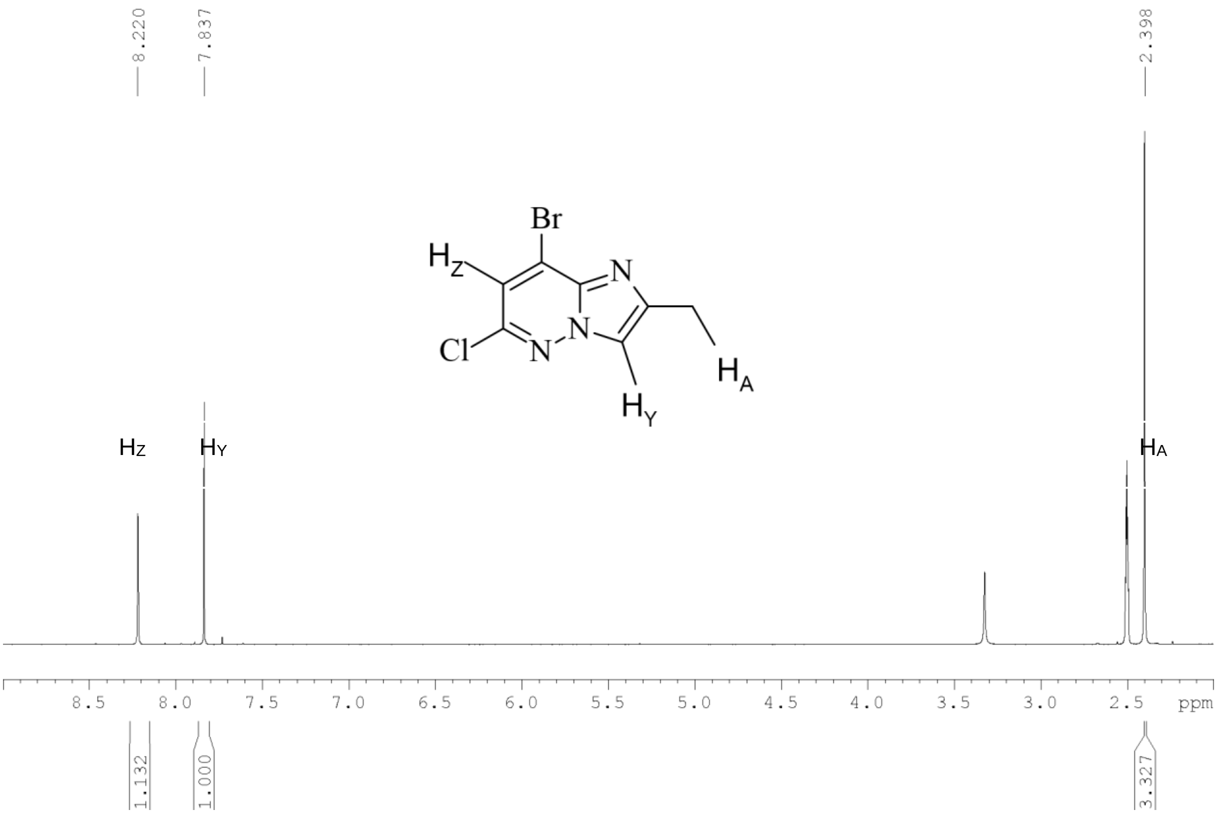

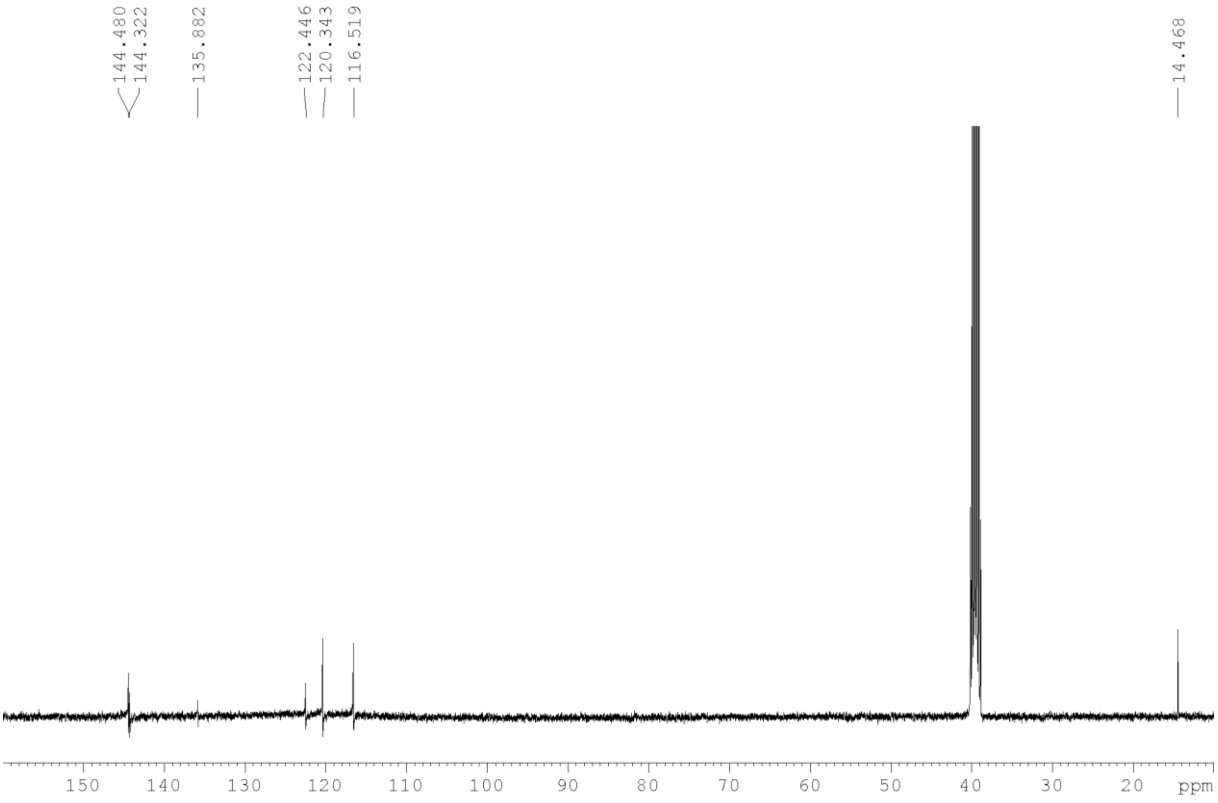

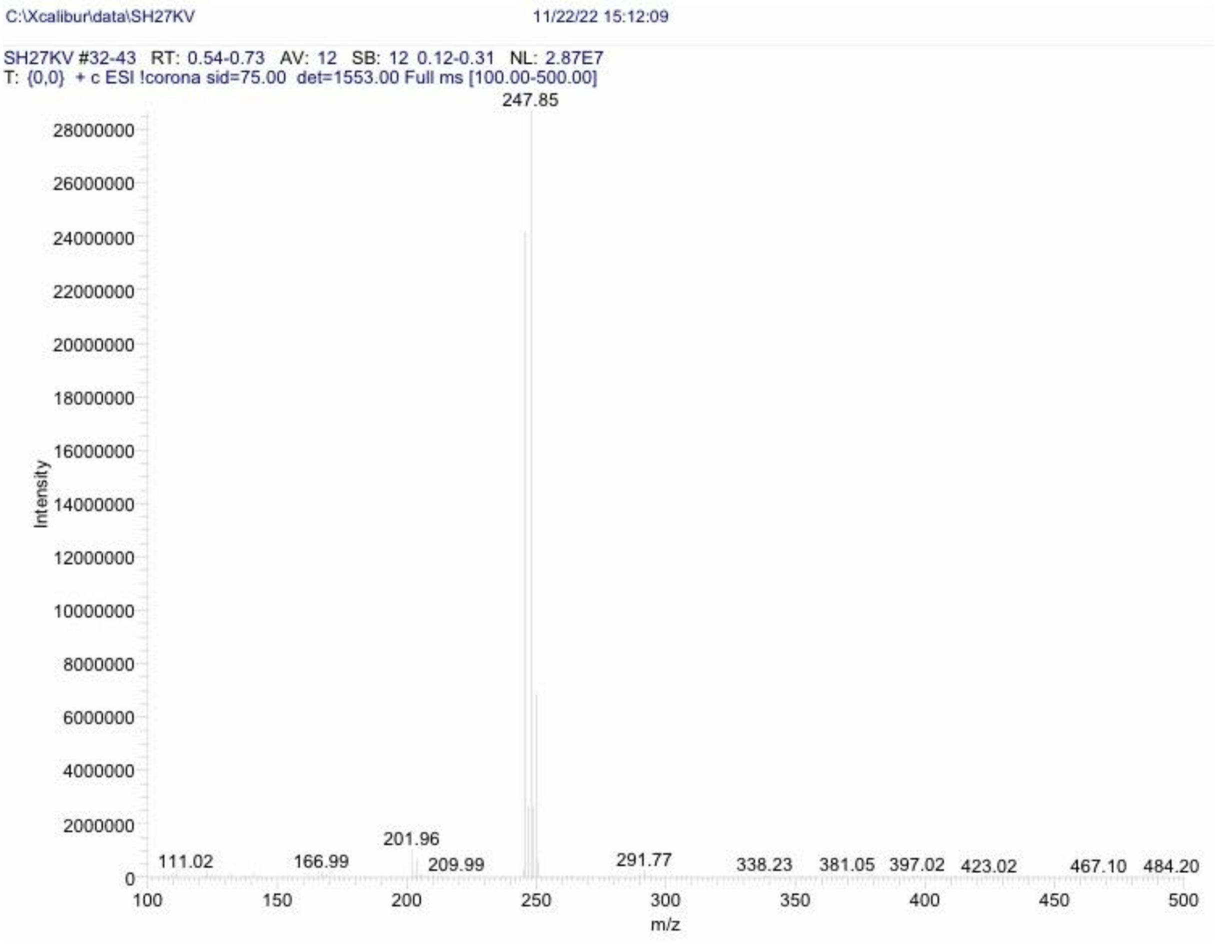
C:\Xcalibur\data\SH27KV
11/22/22 15:12:09
SH27KV #32-43 RT: 0.54-0.73 AV: 12 SB: 12 0.12-0.31 NL: 2.87E7
T: {0,0} + c ESI !corona sid=75.00 det=1553.00 Full ms [100.00-500.00]
247.85
Intensity
201.96
111.02
166.99
209.99
291.77
338.23
381.05
397.02
423.02
467.10
484.20
m/z

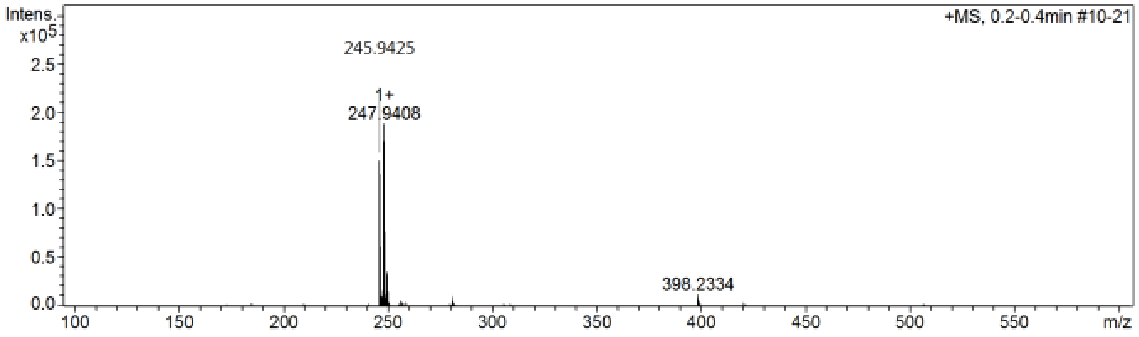
Intens. x10^5
+MS, 0.2-0.4min #10-21
245.9425
1+
247.9408
398.2334
m/z

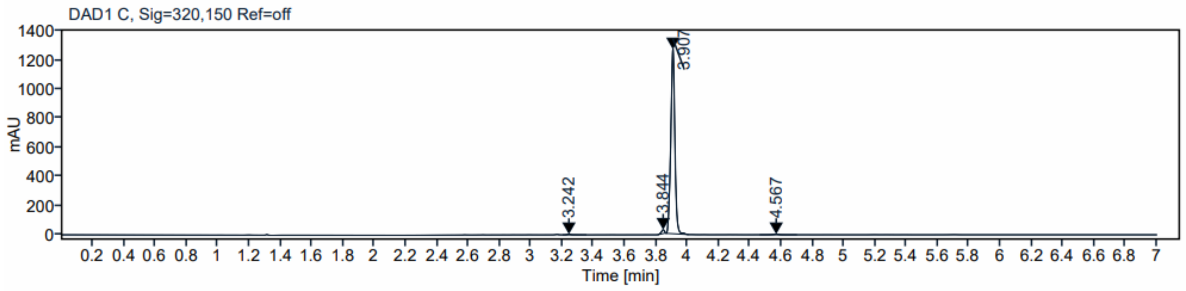
DAD1 C, Sig=320,150 Ref=off
mAU
3.907
3.242
3.844
4.567
Time [min]

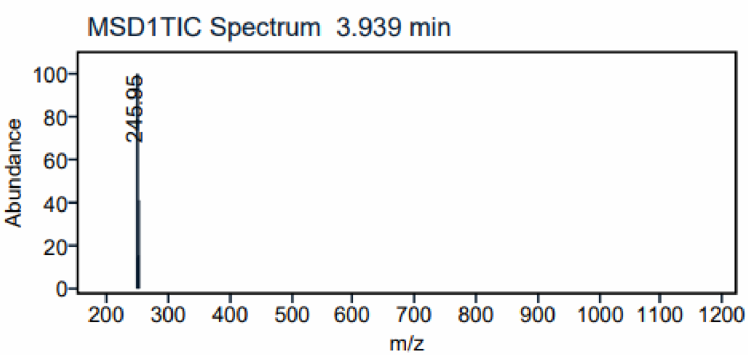

^{1}H-, ^{13}C-NMR-, ESI-, HRMS- and LC-MS-spectrum of 2-[(6-chloro-2-methyl-imidazo[1,2-b]pyridazin-8-yl)amino]ethanethiole **7**

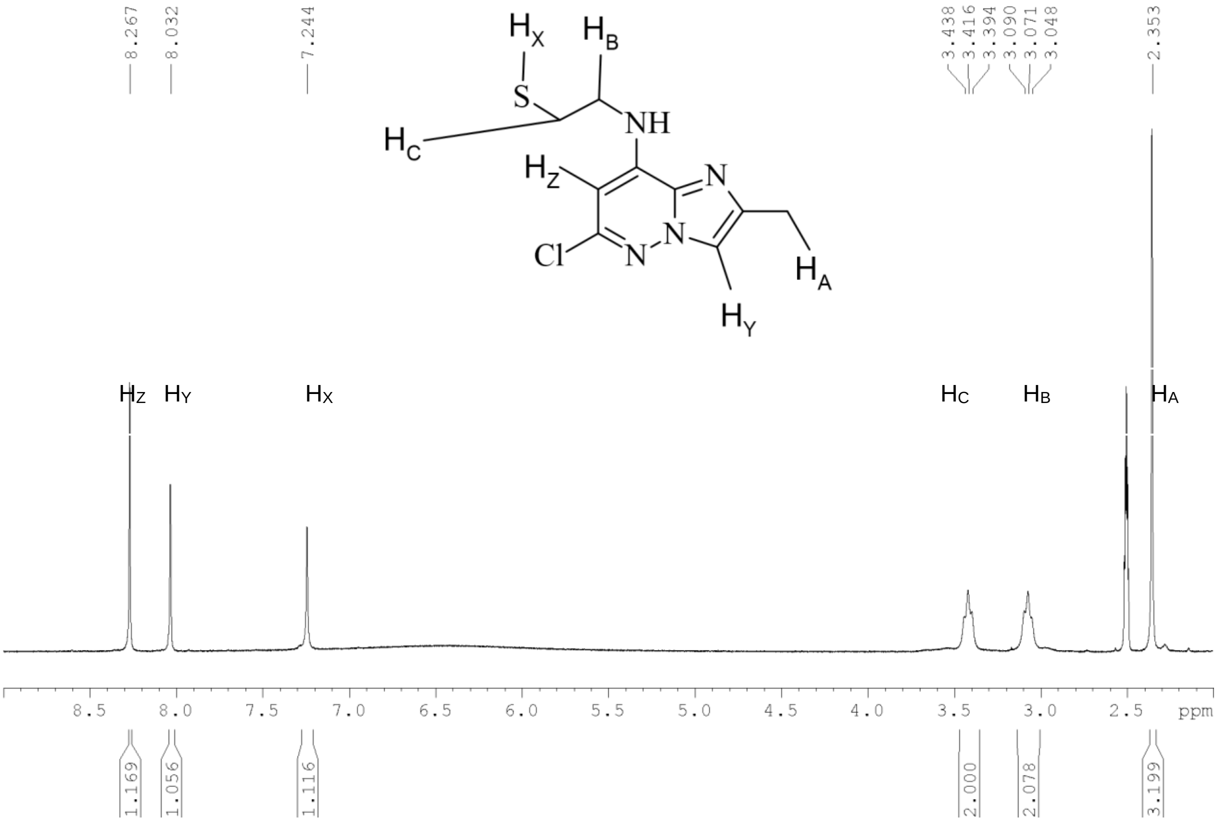

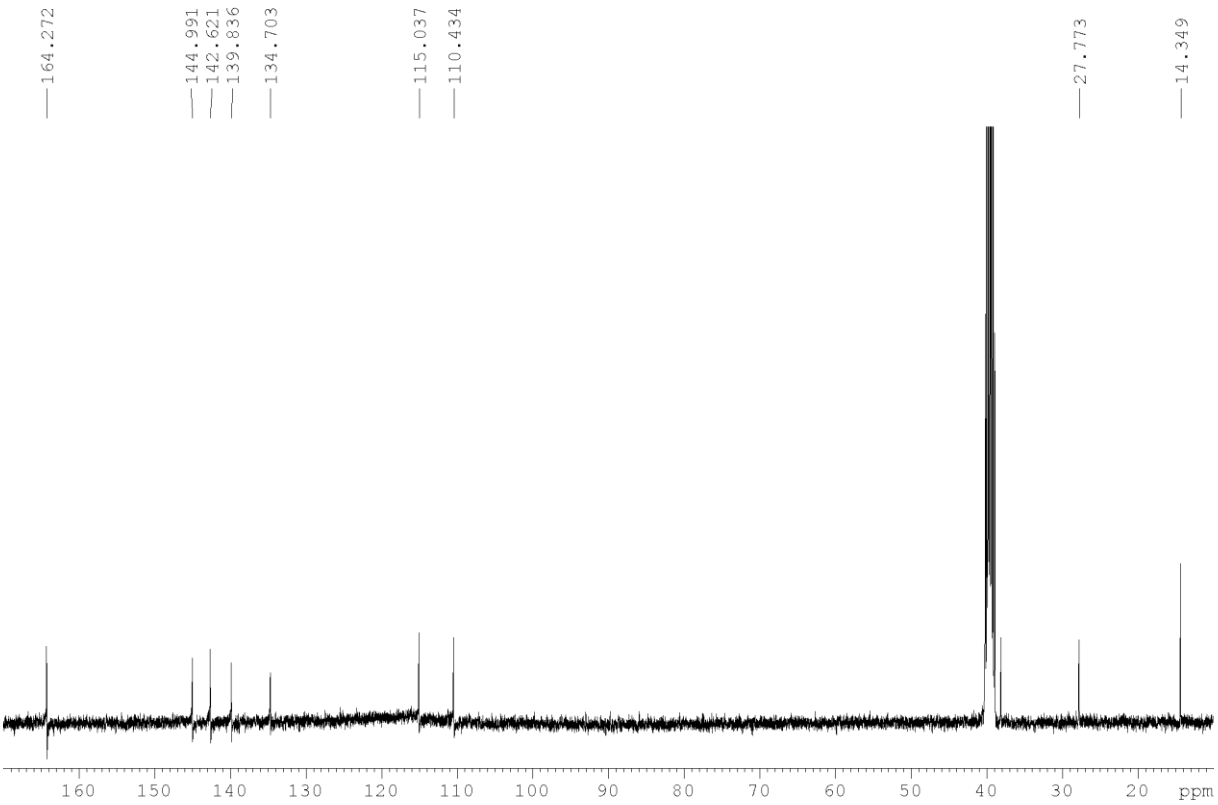
164.272
144.991
142.621
139.836
134.703
115.037
110.434
27.773
14.349
160
150
140
130
120
110
100
90
80
70
60
50
40
30
20
ppm

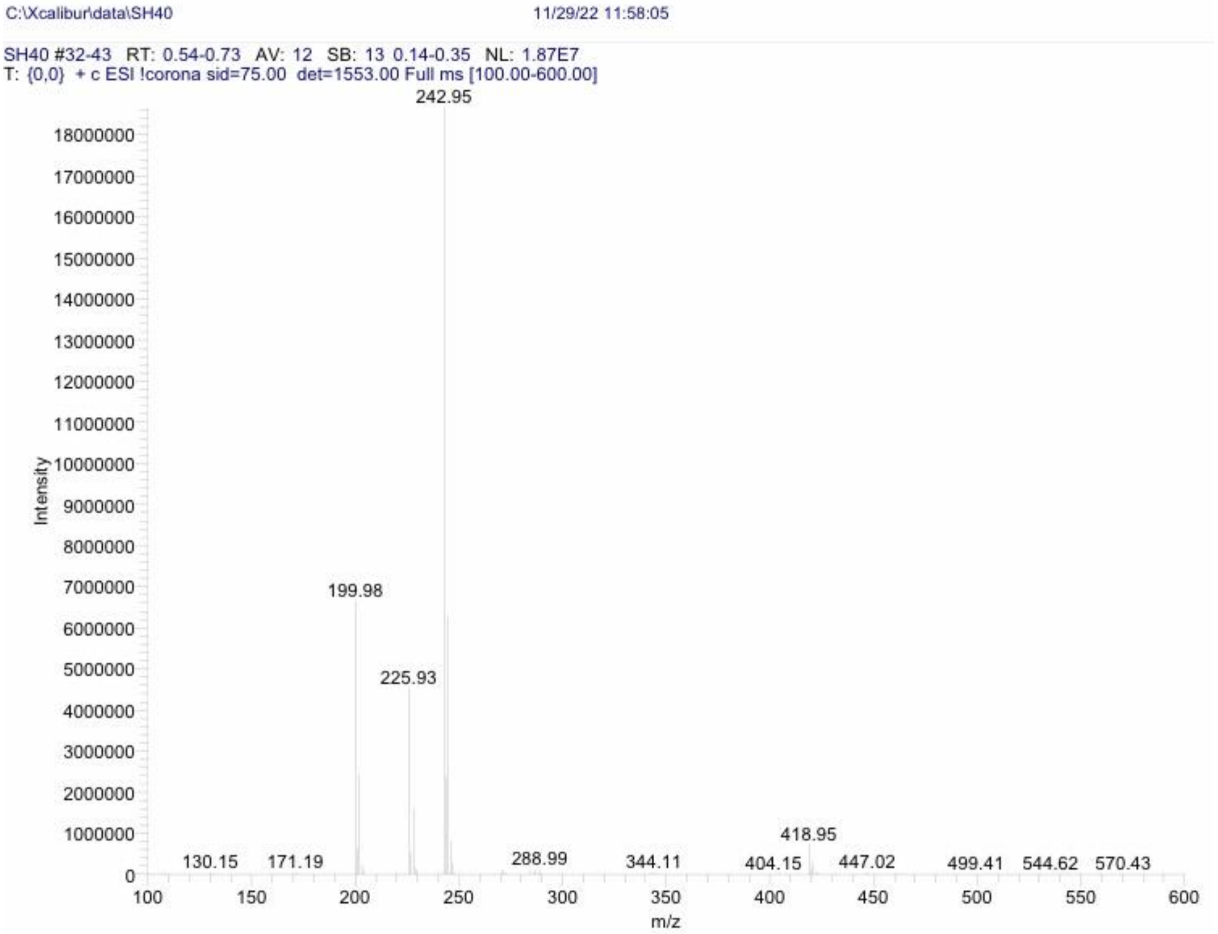
C:\Xcalibur\data\SH40
11/29/22 11:58:05
SH40 #32-43 RT: 0.54-0.73 AV: 12 SB: 13 0.14-0.35 NL: 1.87E7
T: {0,0} + c ESI !corona sid=75.00 det=1553.00 Full ms [100.00-600.00]
242.95
199.98
225.93
130.15
171.19
288.99
344.11
404.15
418.95
447.02
499.41
544.62
570.43
Intensity
m/z

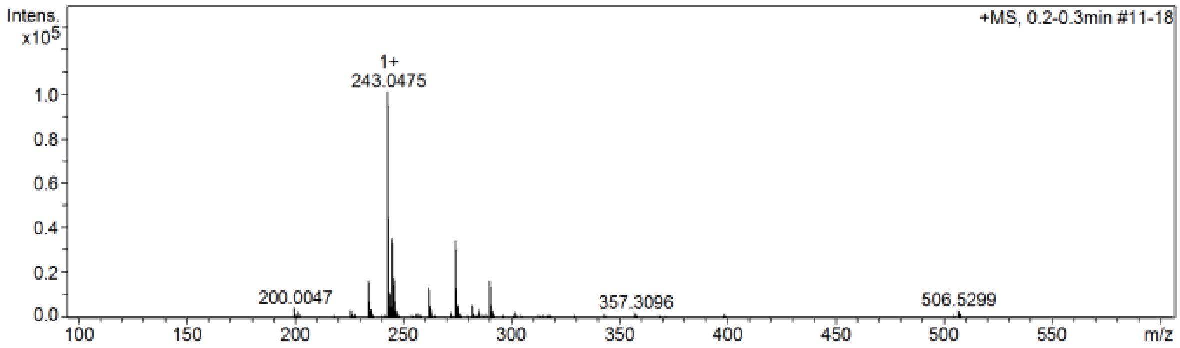
Intens.
x10^5
+MS, 0.2-0.3min #11-18
1+
243.0475
200.0047
357.3096
506.5299
m/z

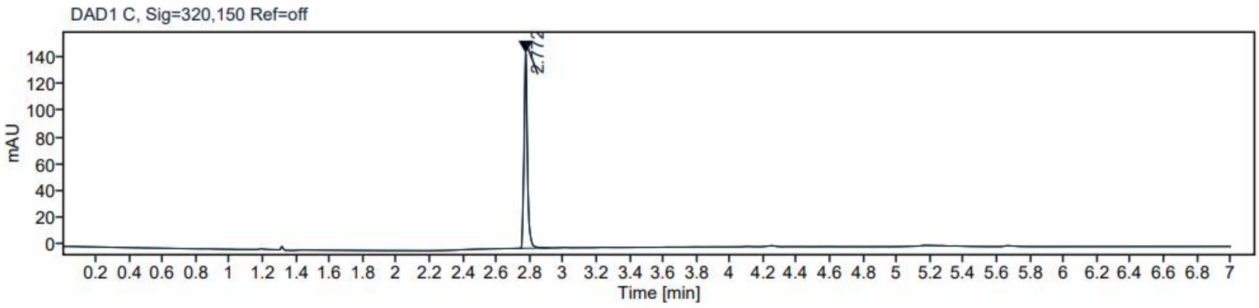
DAD1 C, Sig=320,150 Ref=off
mAU
2.772
Time [min]

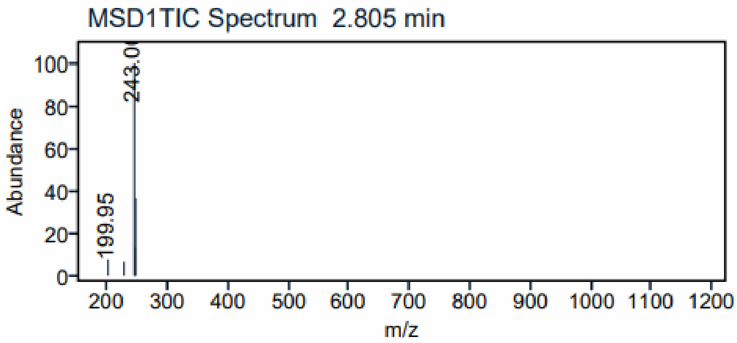
MSD1TIC Spectrum 2.805 min
Abundance
243.0
199.95
m/z

^{1}H-, ^{13}C-NMR-, ESI-, HRMS- and LC-MS-spectrum of 2-[(6-chloro-2-methyl-imidazo[1,2-b]pyridazin-8-yl)amino]ethanethiole **8**

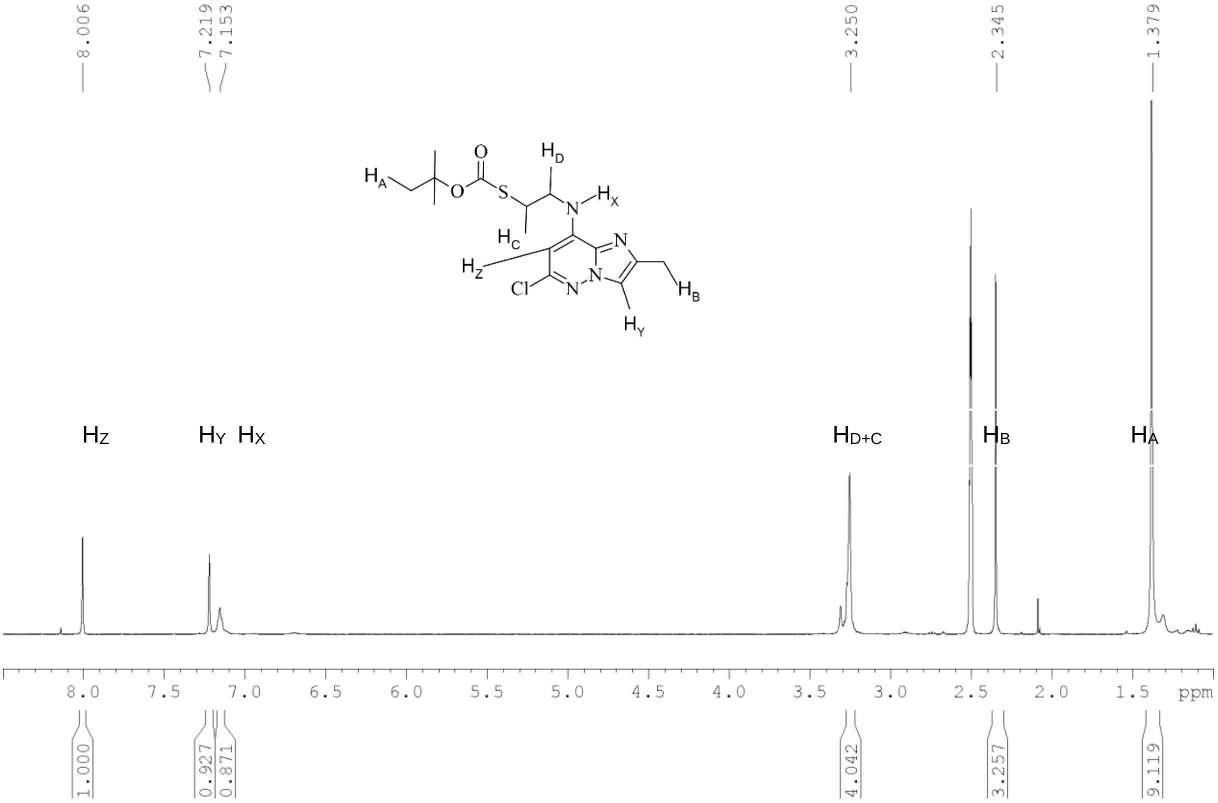

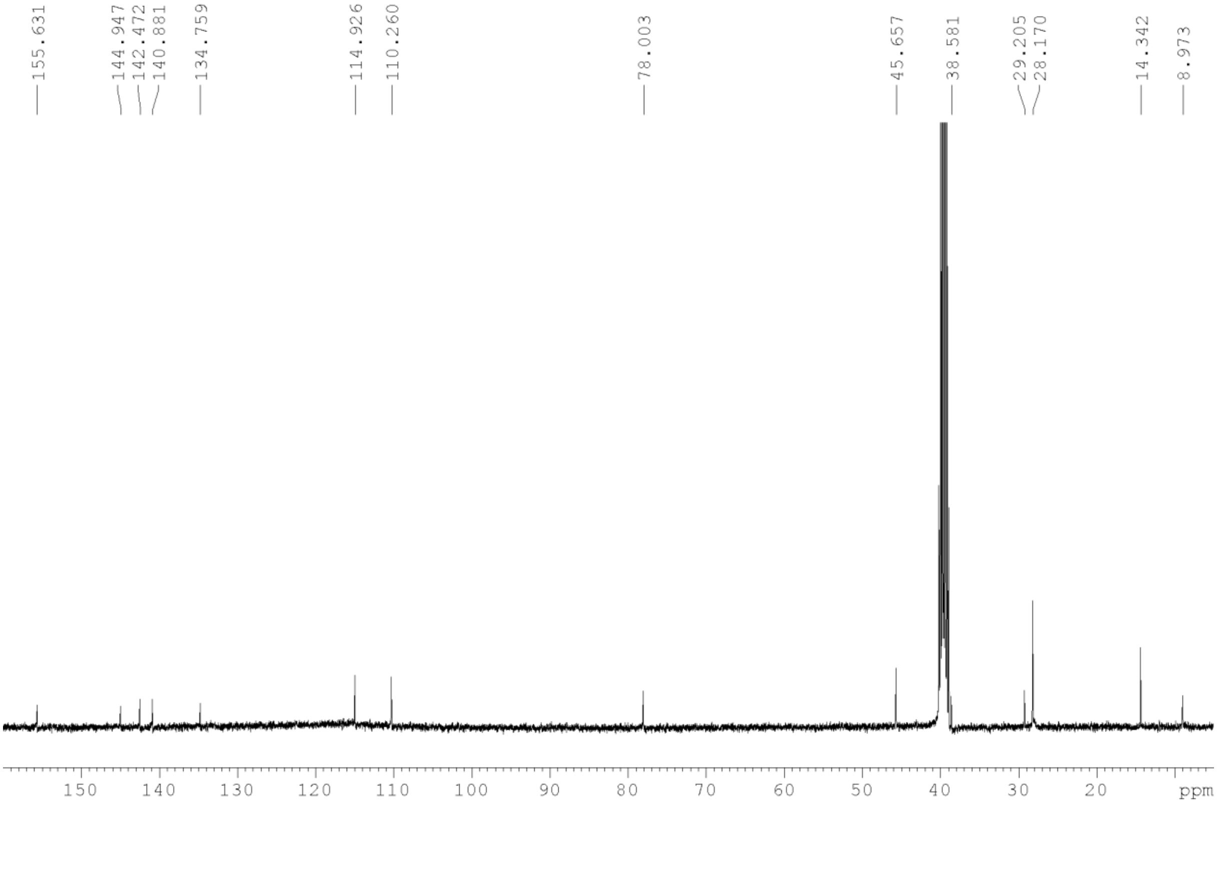

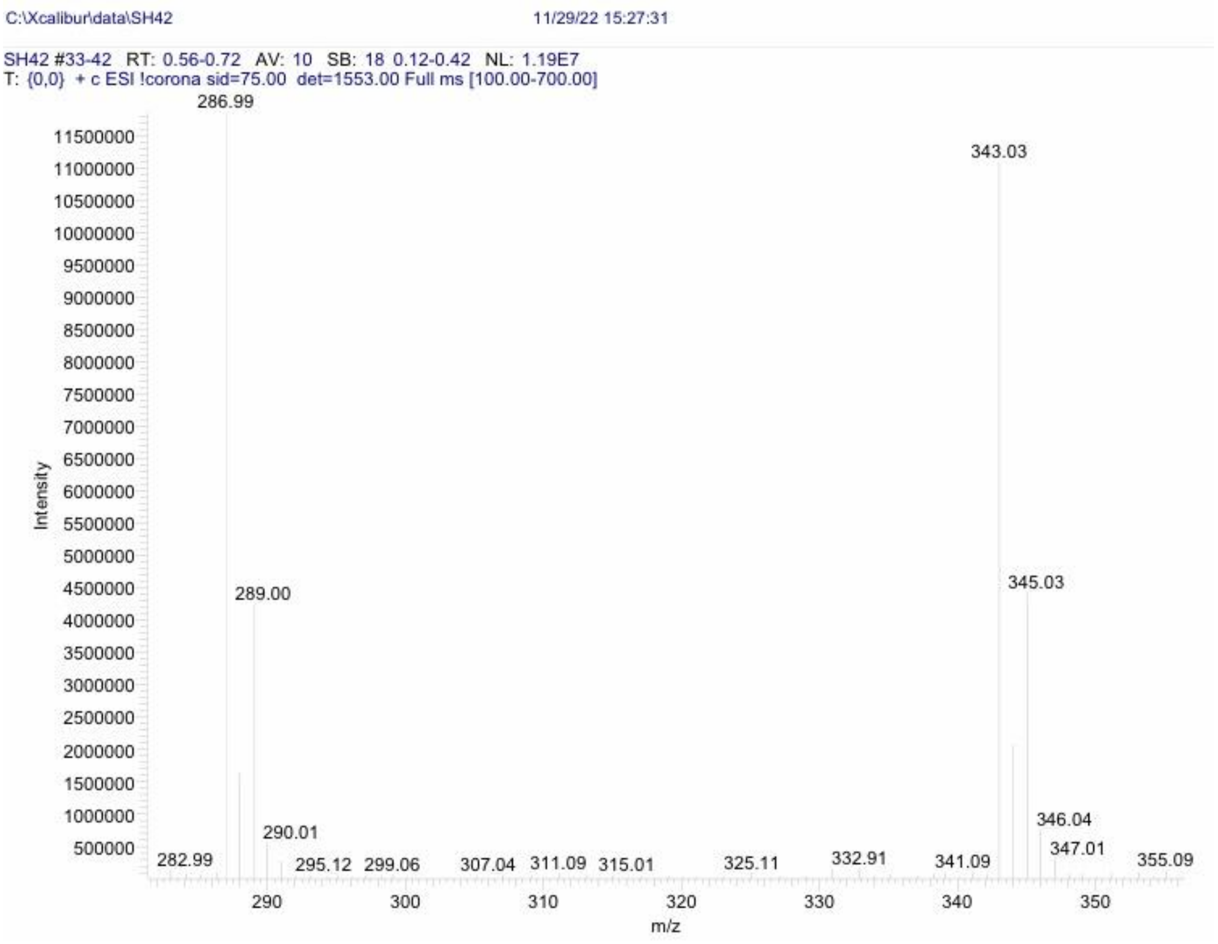
C:\Xcalibur\data\SH42
11/29/22 15:27:31
SH42 #33-42 RT: 0.56-0.72 AV: 10 SB: 18 0.12-0.42 NL: 1.19E7
T: {0,0} + c ESI !corona sid=75.00 det=1553.00 Full ms [100.00-700.00]
Intensity
m/z
286.99
343.03
345.03
289.00
290.01
346.04
347.01
282.99
295.12
299.06
307.04
311.09
315.01
325.11
332.91
341.09
355.09

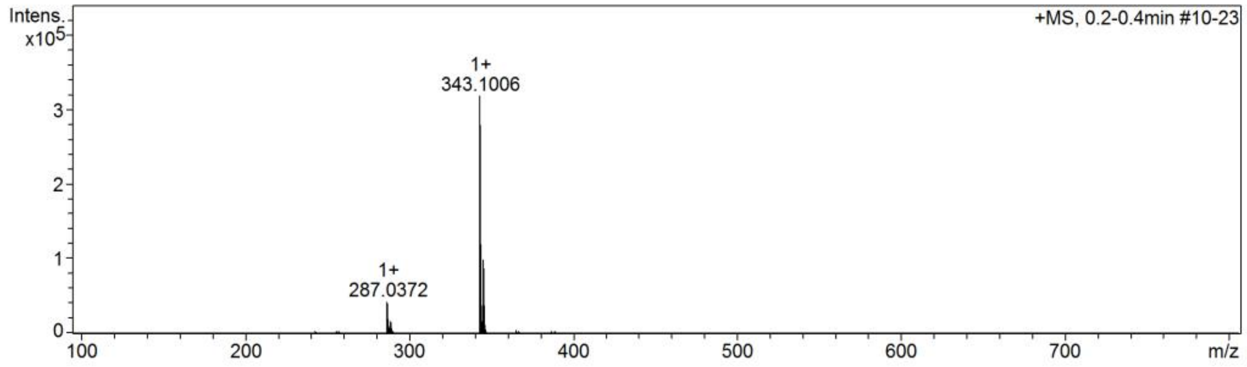
Intens.
x10^5
+MS, 0.2-0.4min #10-23
1+
343.1006
1+
287.0372
m/z

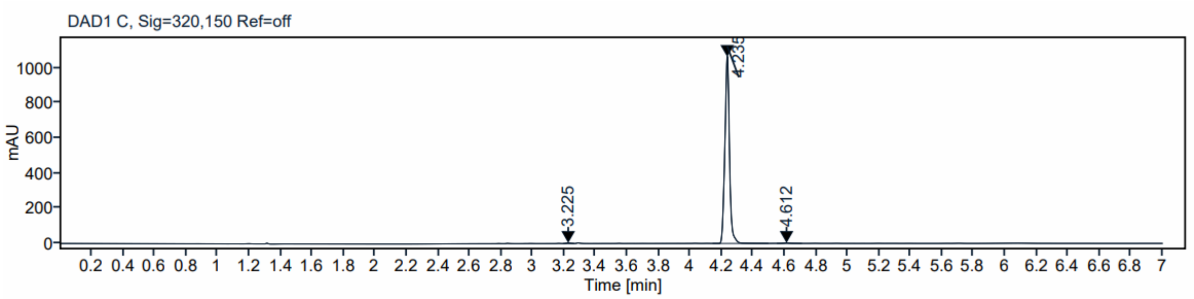

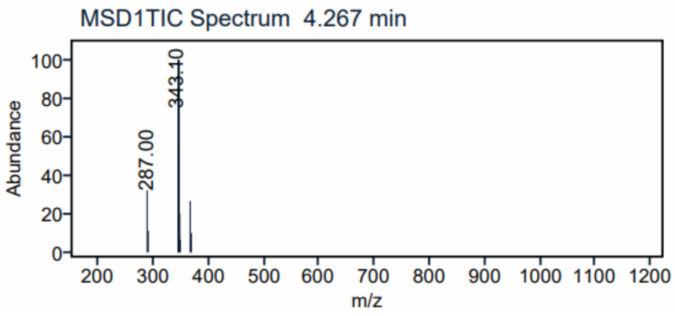

^{1}H-, ^{13}C-, ^{19}F-NMR-, ESI-, HRMS- and LC-MS-spectrum of 2-Chloro-7-fluoro-4*H*-pyrido[1,2-*a*]pyrimidin-4-one **9**

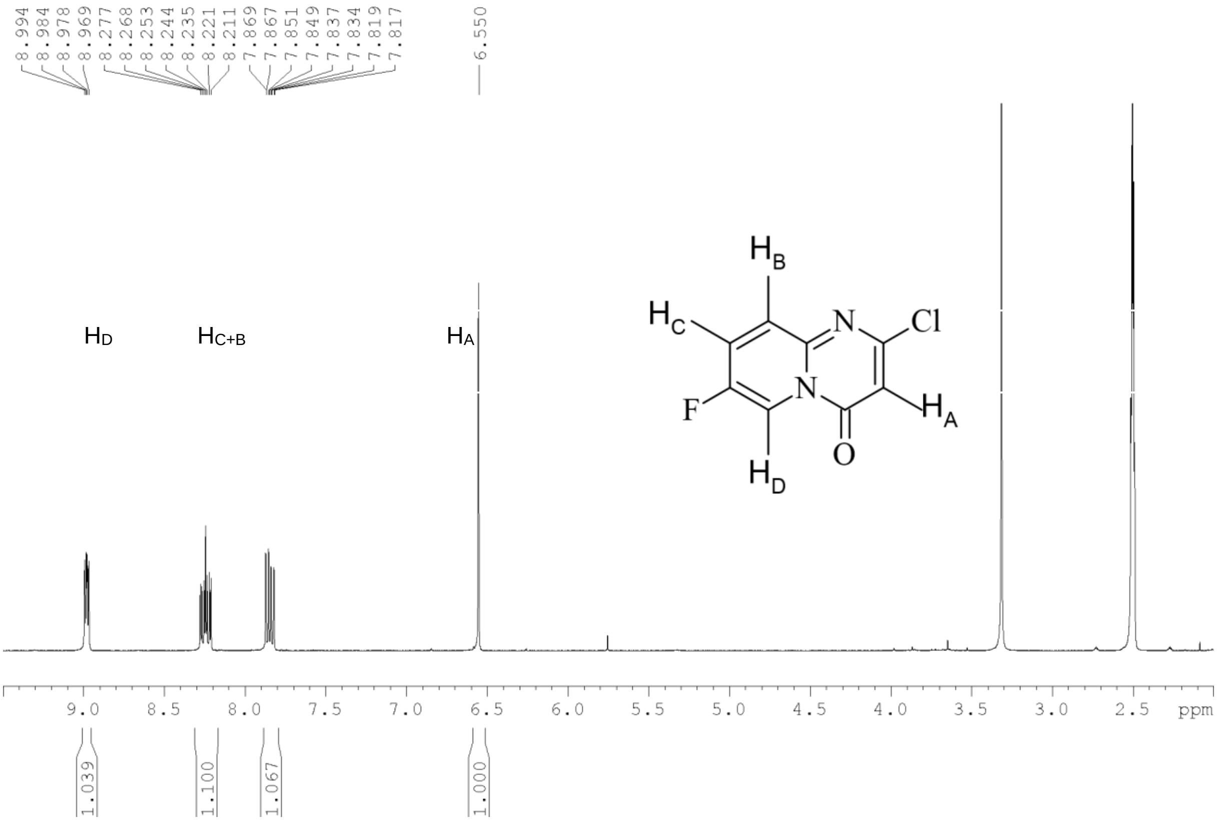

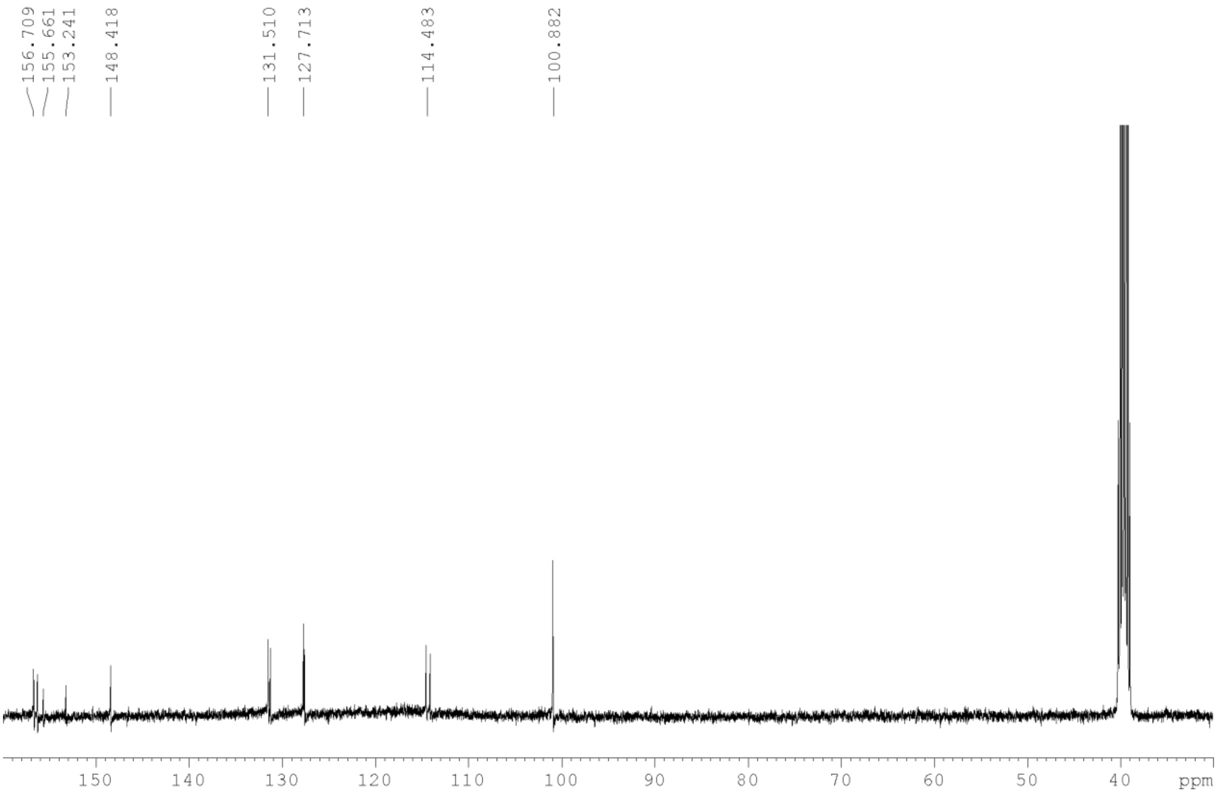
156.709
155.661
153.241
148.418
131.510
127.713
114.483
100.882
150
140
130
120
110
100
90
80
70
60
50
40
ppm

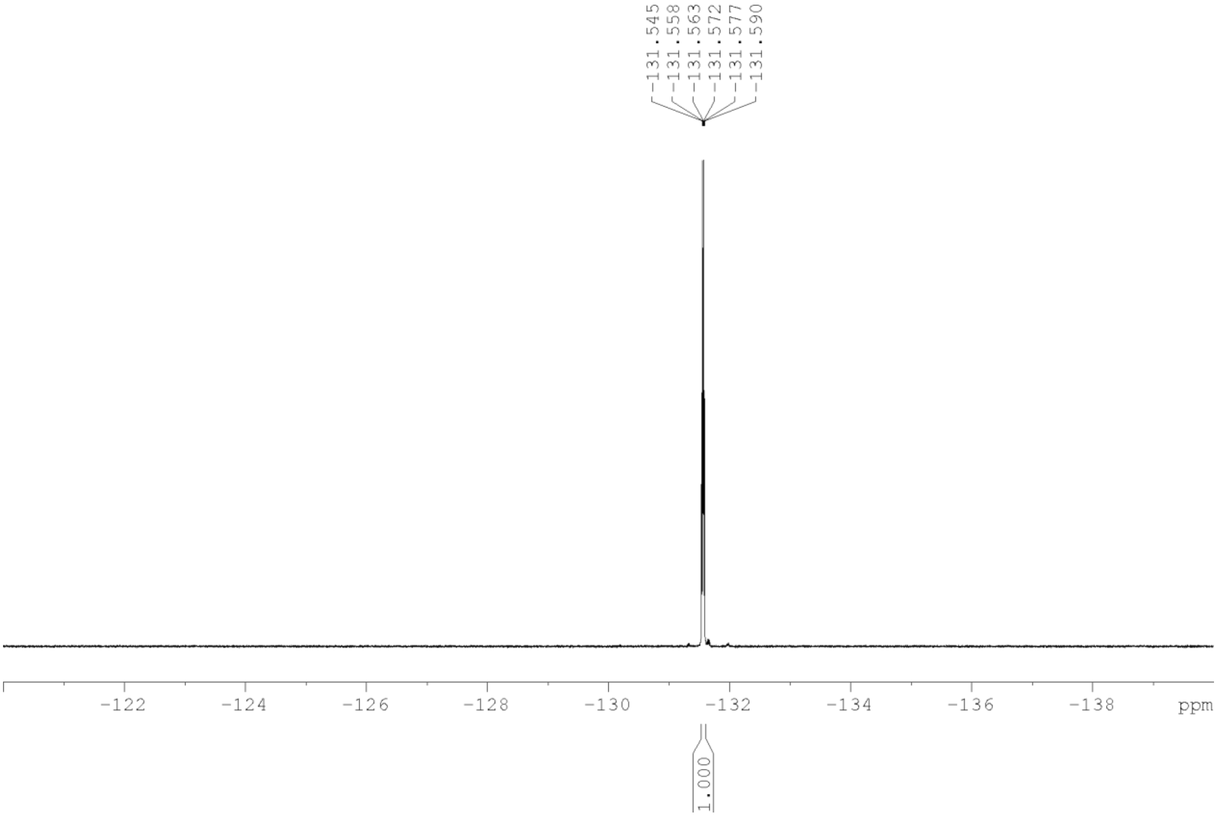
-131.545
-131.558
-131.563
-131.572
-131.577
-131.590
-122
-124
-126
-128
-130
-132
-134
-136
-138
ppm
1.000

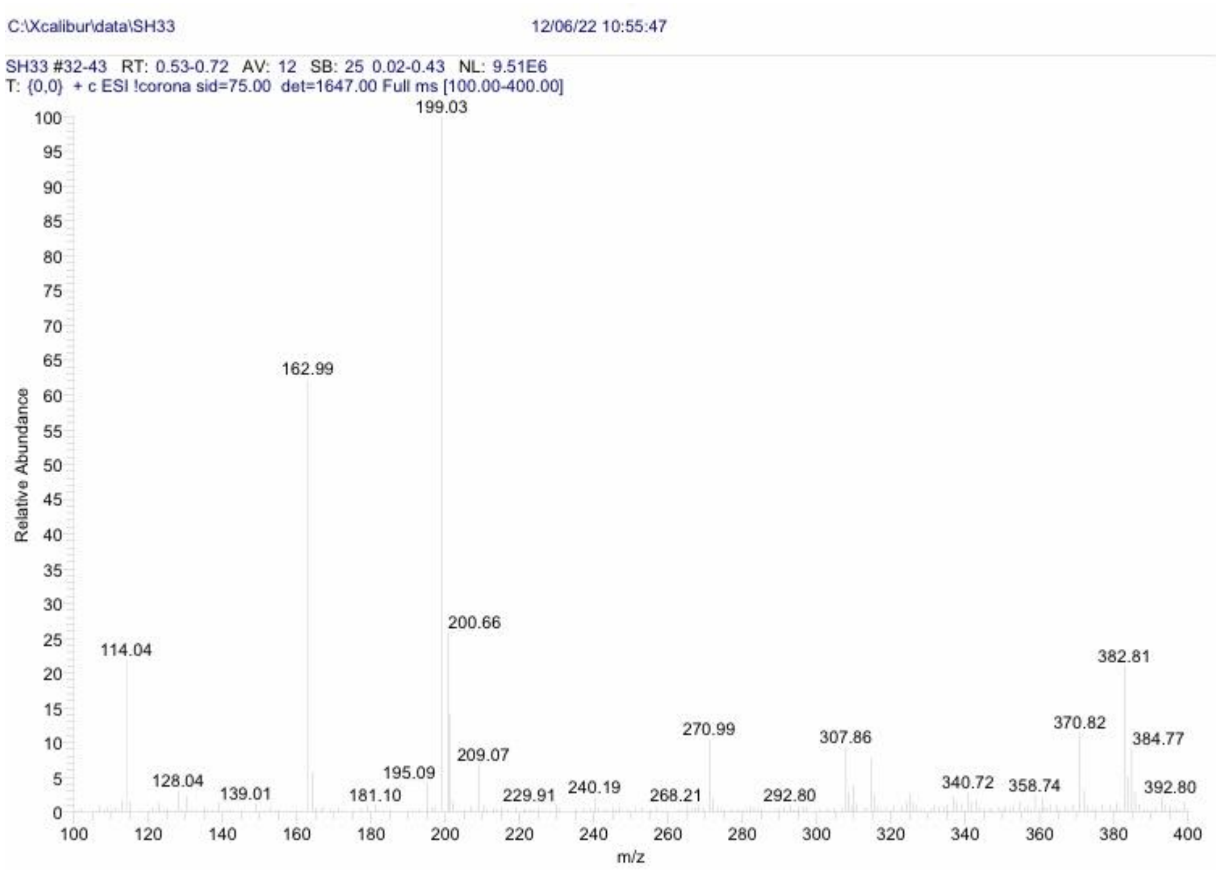
C:\Xcalibur\data\SH33
12/06/22 10:55:47
SH33 #32-43 RT: 0.53-0.72 AV: 12 SB: 25 0.02-0.43 NL: 9.51E6
T: {0,0} + c ESI !corona sid=75.00 det=1647.00 Full ms [100.00-400.00]
Relative Abundance
m/z
199.03
162.99
200.66
114.04
382.81
370.82
270.99
307.86
384.77
209.07
195.09
128.04
139.01
181.10
229.91
240.19
268.21
292.80
340.72
358.74
392.80

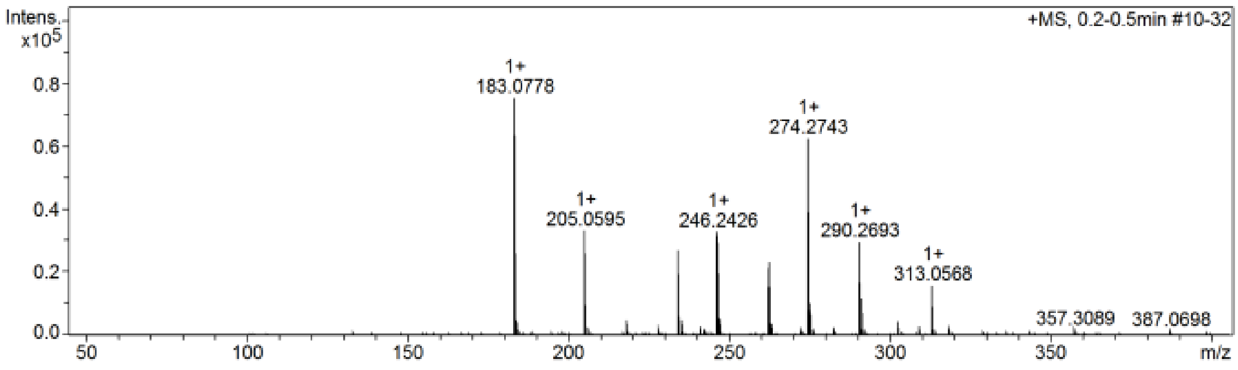
Intens.
x10^5
+MS, 0.2-0.5min #10-32
1+ 183.0778
1+ 205.0595
1+ 246.2426
1+ 274.2743
1+ 290.2693
1+ 313.0568
357.3089
387.0698
m/z

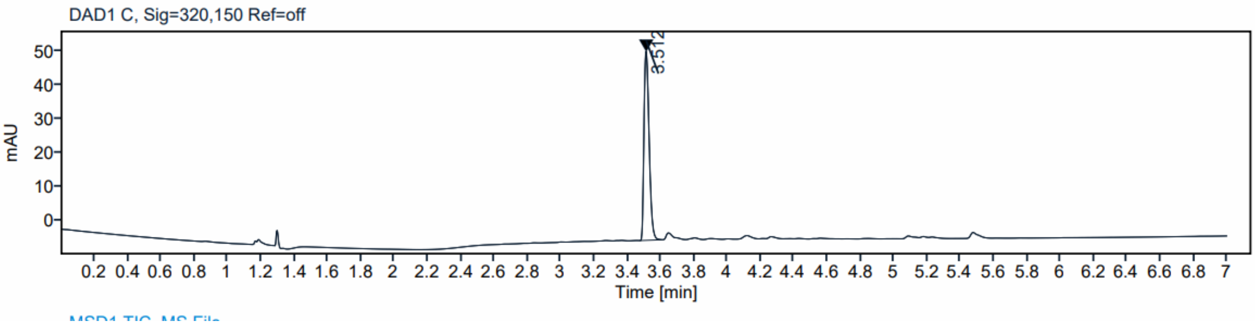
DAD1 C, Sig=320,150 Ref=off
mAU
3.512
Time [min]

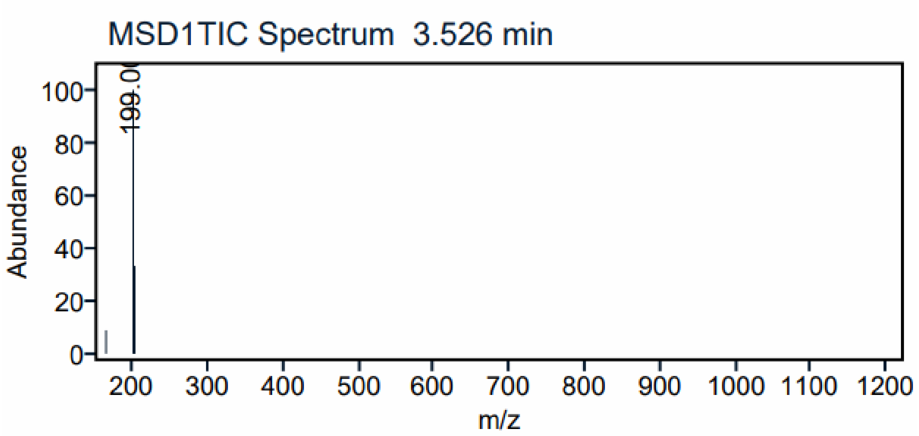

<u>^{1}H-NMR-, ESI-, HRMS- and LC-MS-spectrum of 7-Fluoro-2-hydroxy-pyrido[1,2-a]pyrimidin-4-one **12**</u>

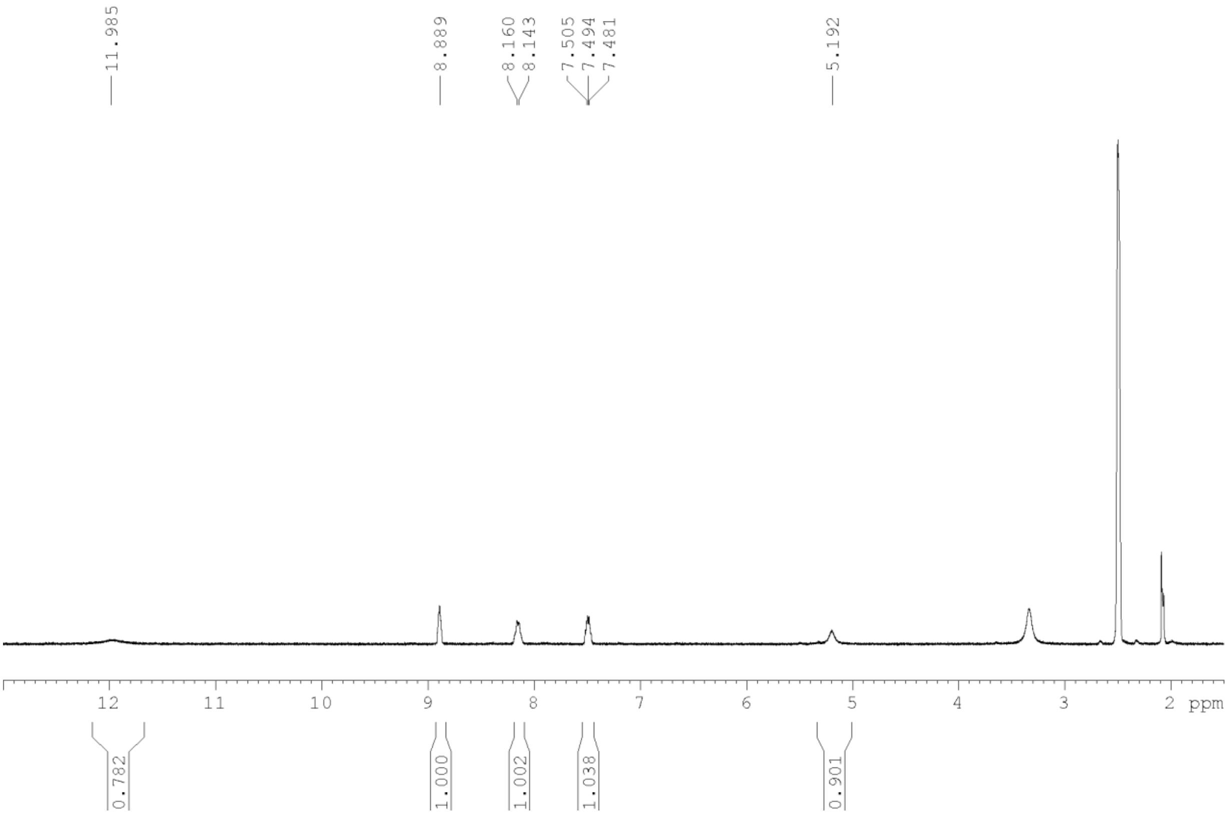

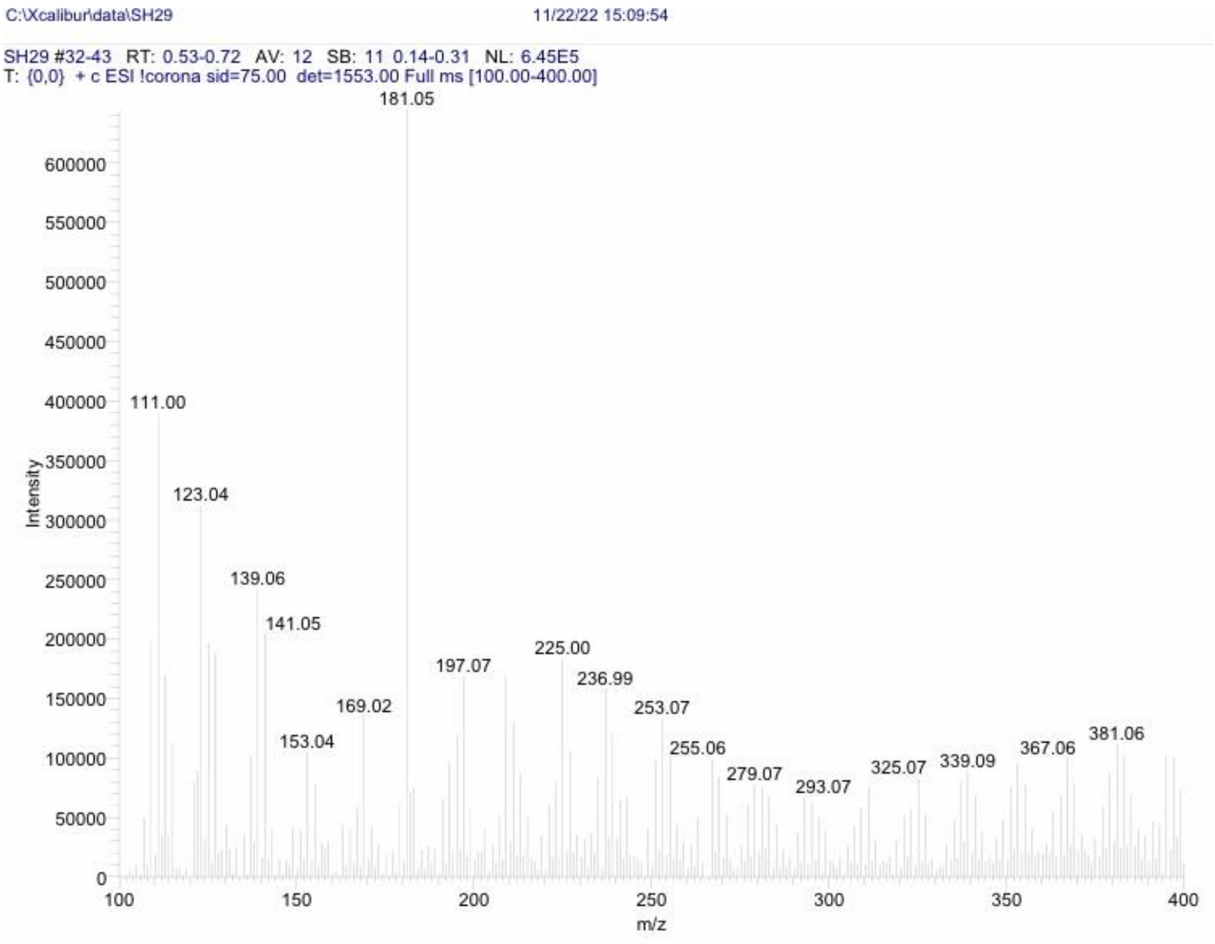
C:\Xcalibur\data\SH29
11/22/22 15:09:54
SH29 #32-43 RT: 0.53-0.72 AV: 12 SB: 11 0.14-0.31 NL: 6.45E5
T: {0,0} + c ESI !corona sid=75.00 det=1553.00 Full ms [100.00-400.00]
181.05
111.00
123.04
139.06
141.05
153.04
169.02
197.07
225.00
236.99
253.07
255.06
279.07
293.07
325.07
339.09
367.06
381.06
Intensity
m/z

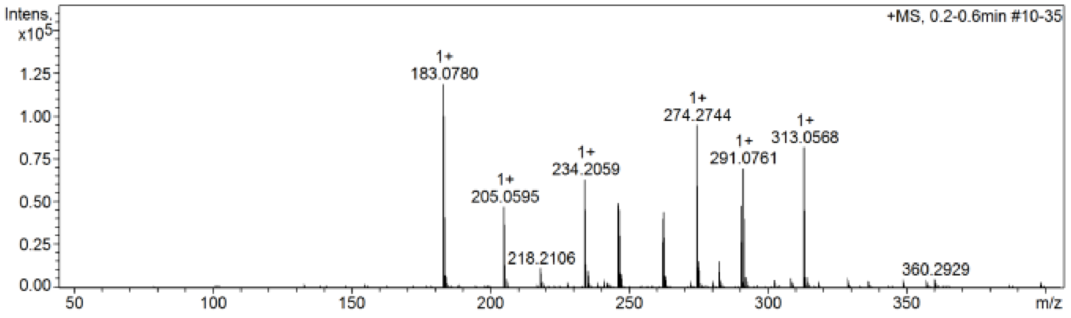
Intens.
x10^5
+MS, 0.2-0.6min #10-35
183.0780
205.0595
218.2106
234.2059
274.2744
291.0761
313.0568
360.2929
m/z

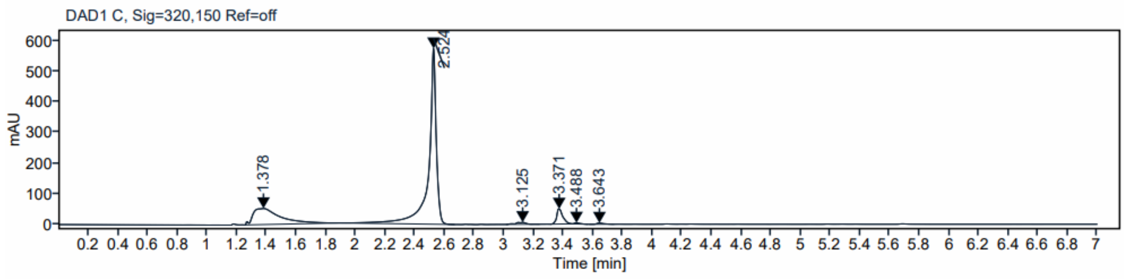
DAD1 C, Sig=320,150 Ref=off
mAU
1.378
2.524
3.125
3.371
3.488
3.643
Time [min]

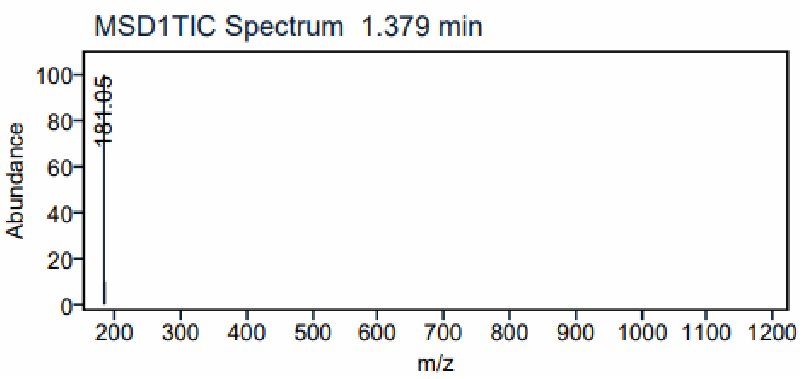

HRMS- and LC-MS-spectrum of 7-fluoro-2-(2-methylimidazo[1,2-b]pyridazin-6-yl)pyrido[1,2-a]pyrimidin-4-one **15**

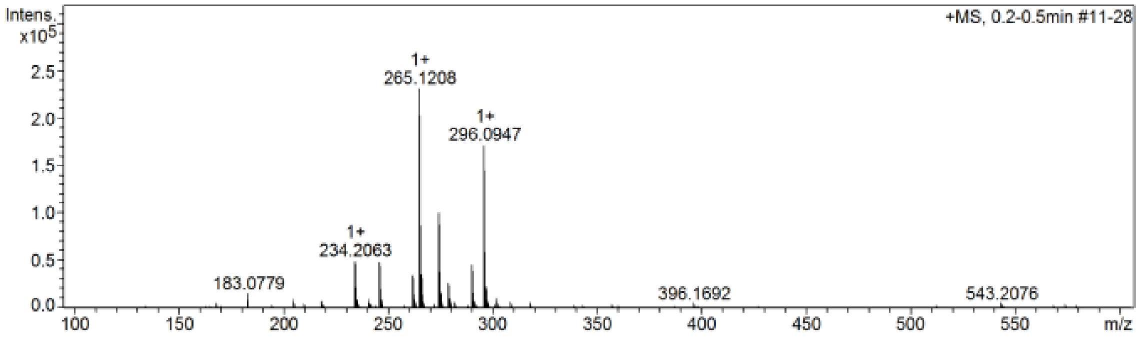

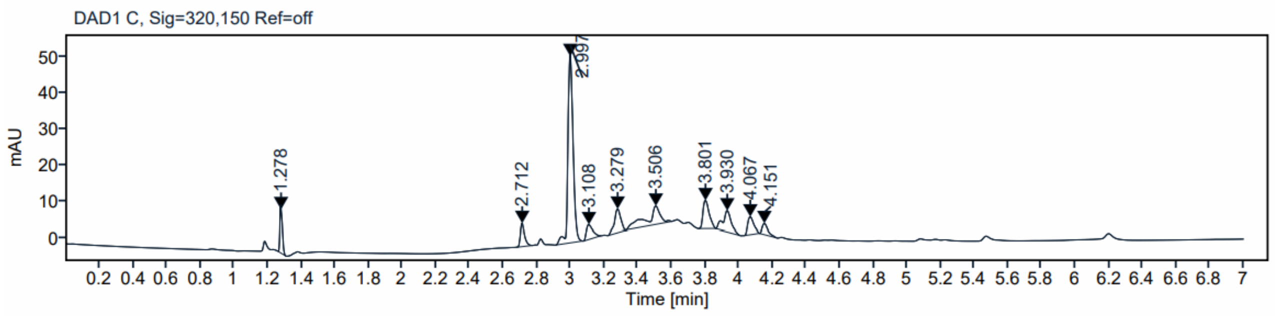

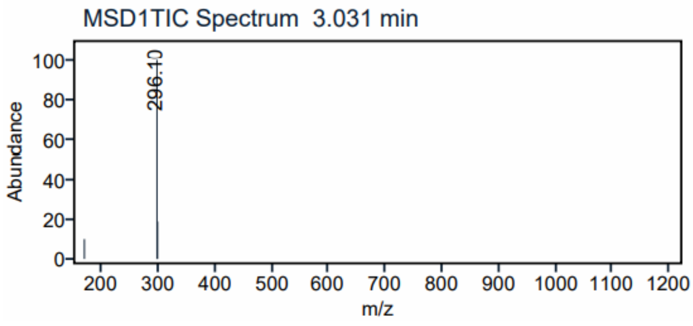

HRMS- and LC-ESI-MS-spectrum of *tert*-butyl 4-[2-(2-methylimidazo[1,2-b]pyridazin-6-yl)-4-oxo-pyrido-[1,2-a]pyrimidin-7-yl]piperazine-1-carboxylate and ^{1}H-, HRMS- and LC-ESI-MS-spectrum of 2-(2-Methylimidazo[1,2-b]pyridazin-6-yl)-7-piperazin-1-yl-pyrido[1,2-a]pyrimidin-4-one **17**

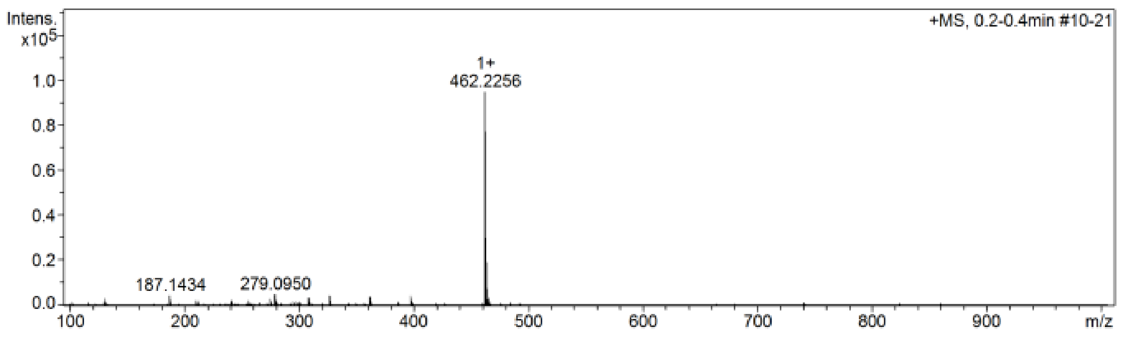

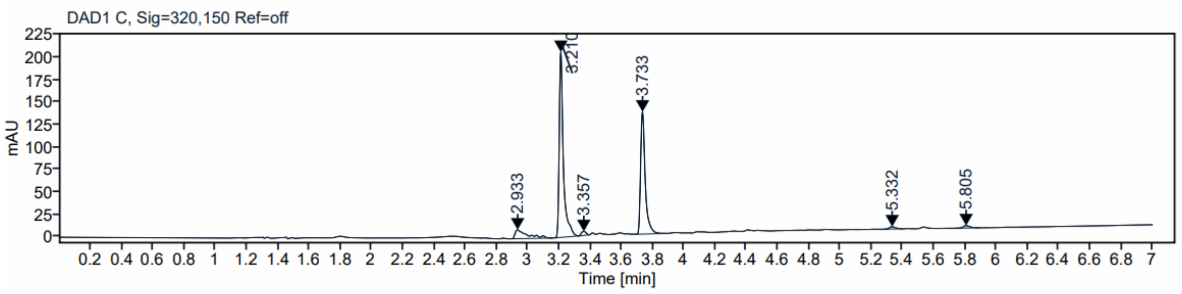

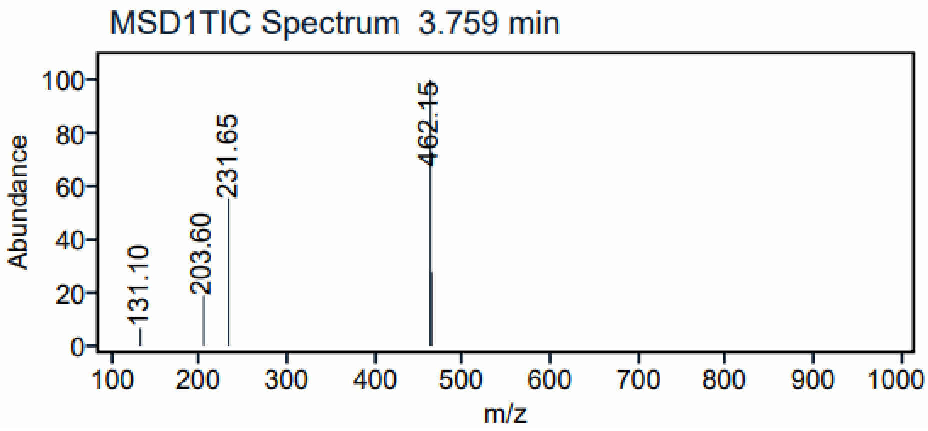

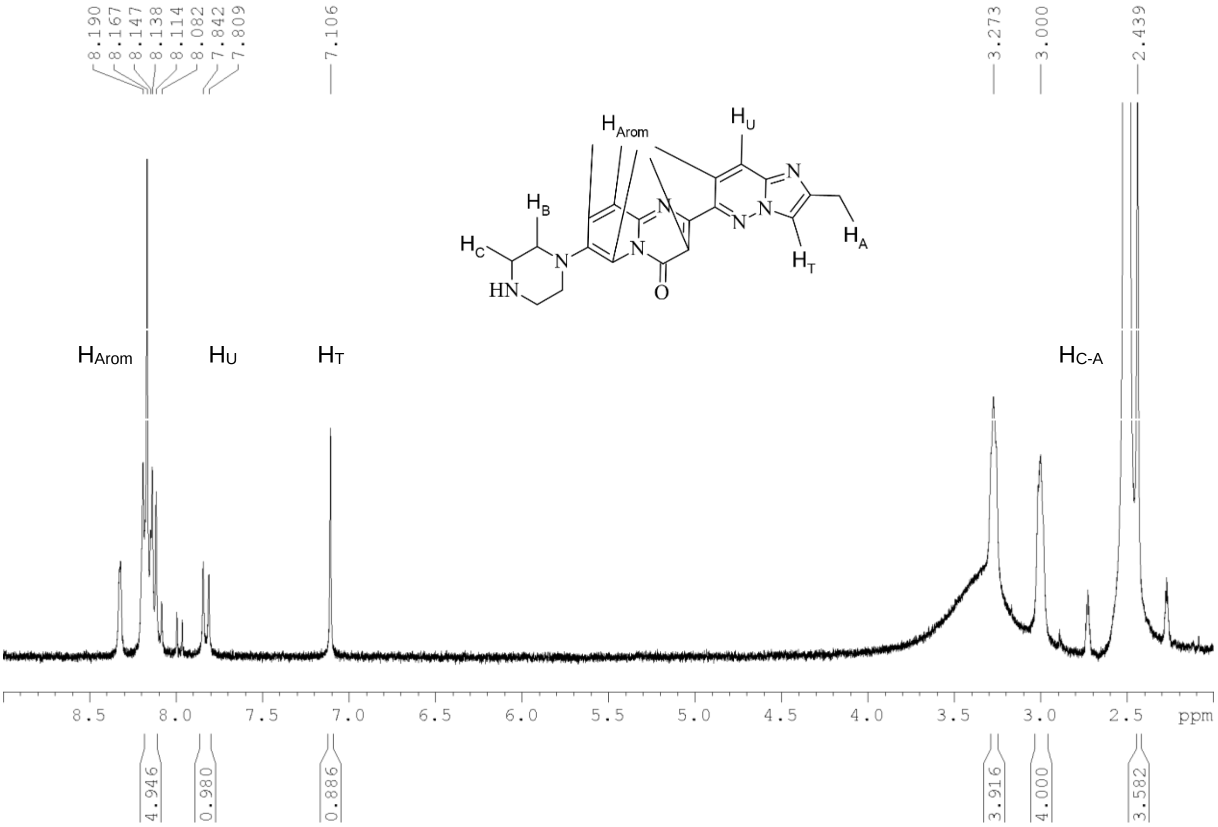
8.190
8.167
8.147
8.138
8.114
8.082
7.842
7.809
7.106
3.273
3.000
2.439
HArom
HU
HT
HC-A
HB
HC
HA
ppm
4.946
0.980
0.886
3.916
4.000
3.582

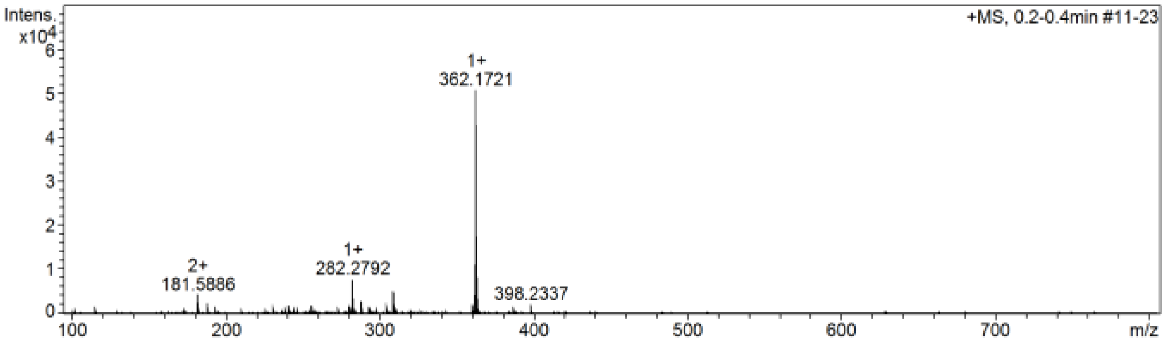
Intens.
x10^4
+MS, 0.2-0.4min #11-23
1+
362.1721
1+
282.2792
2+
181.5886
398.2337
m/z

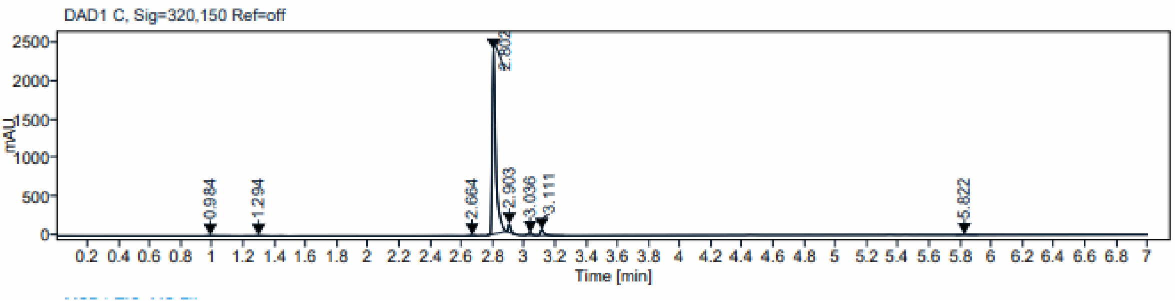
DAD1 C, Sig=320,150 Ref=off
mAU
0.984
1.294
2.664
2.802
2.903
3.036
3.111
5.822
Time [min]

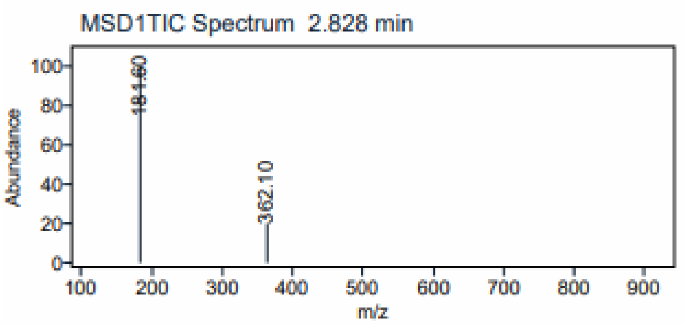

<u>^{1}H-, ^{19}F-, ESI-, MALDI- and LC-MS spectrum of 3-[2,3,5,6-tetrafluoro-4-(2,3,4,5,6-penta-fluorophenyl)phenyl]sulfanylpropanic acid **18**</u>

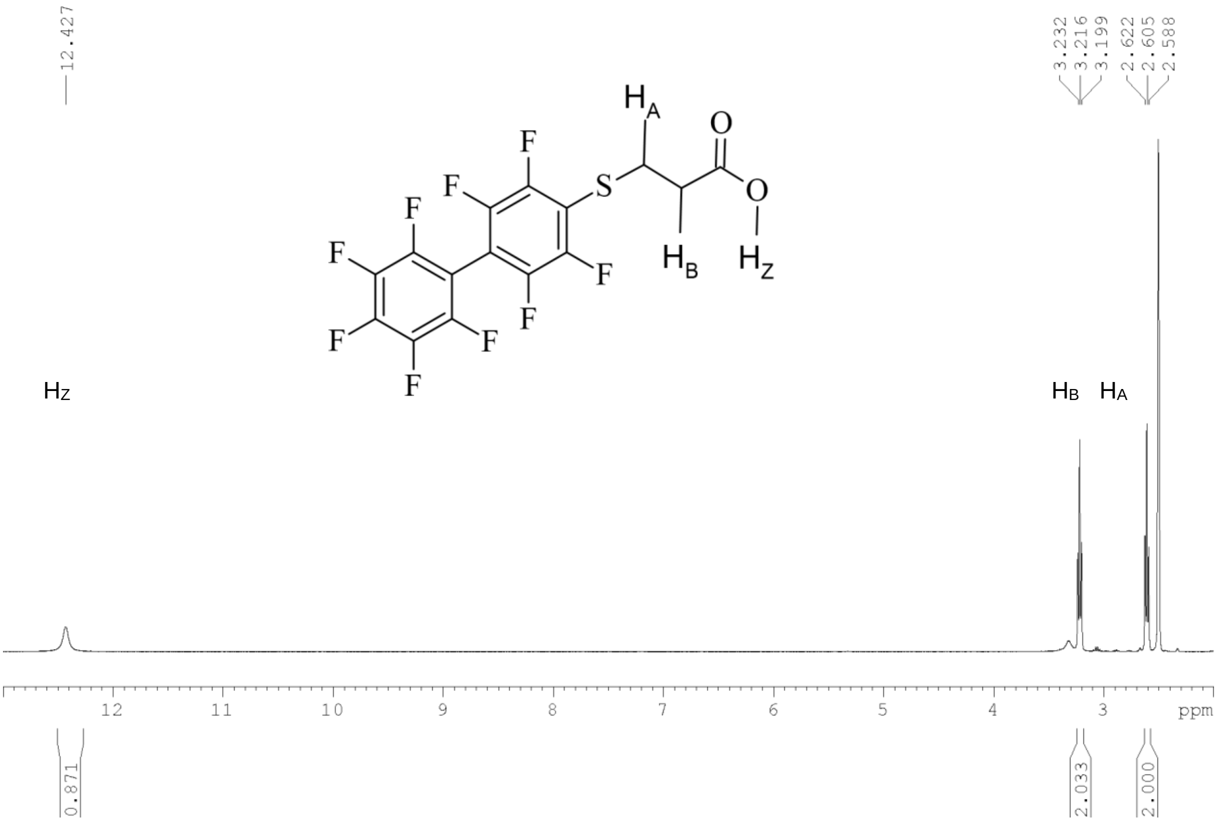

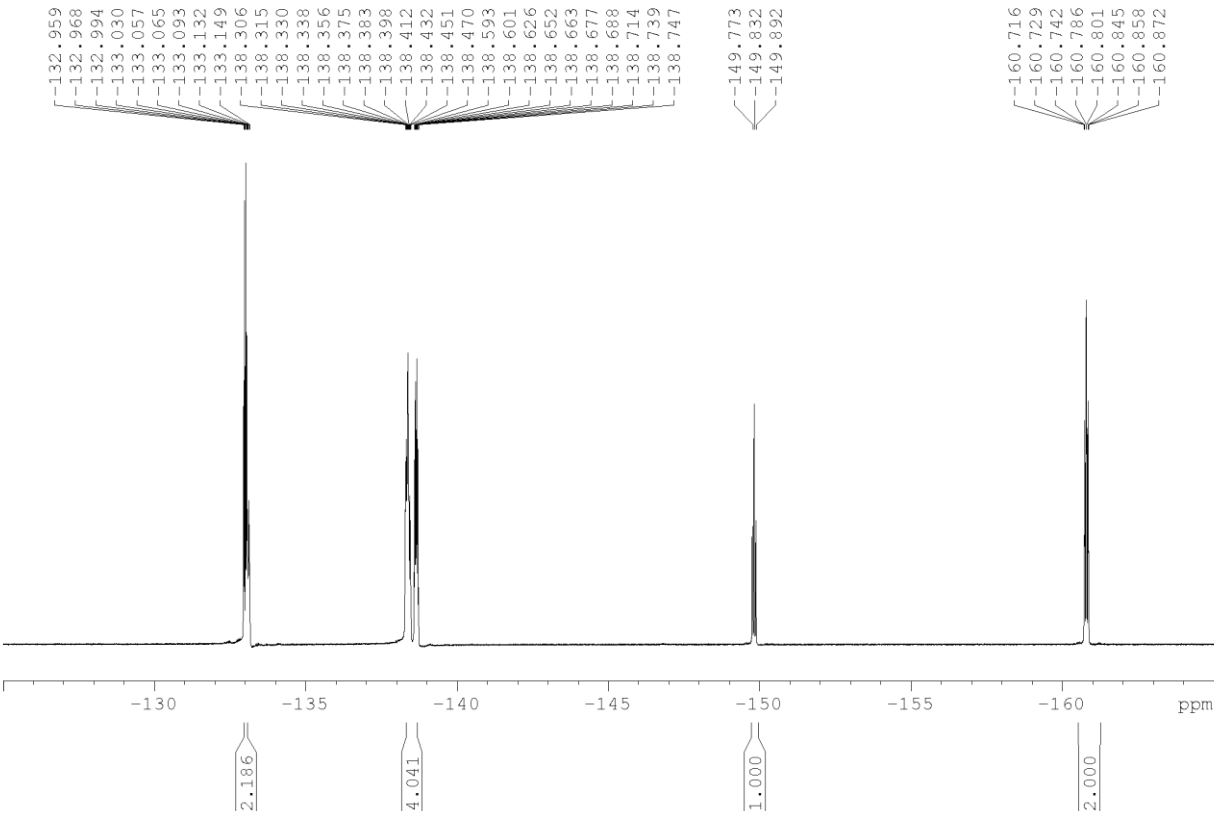

-132.959
-132.968
-132.994
-133.030
-133.057
-133.065
-133.093
-133.132
-133.149
-138.306
-138.315
-138.330
-138.338
-138.356
-138.375
-138.383
-138.398
-138.412
-138.432
-138.451
-138.470
-138.593
-138.601
-138.626
-138.652
-138.663
-138.677
-138.688
-138.714
-138.739
-138.747
-149.773
-149.832
-149.892
-160.716
-160.729
-160.742
-160.786
-160.801
-160.845
-160.858
-160.872
-130
-135
-140
-145
-150
-155
-160
ppm
2.186
4.041
1.000
2.000

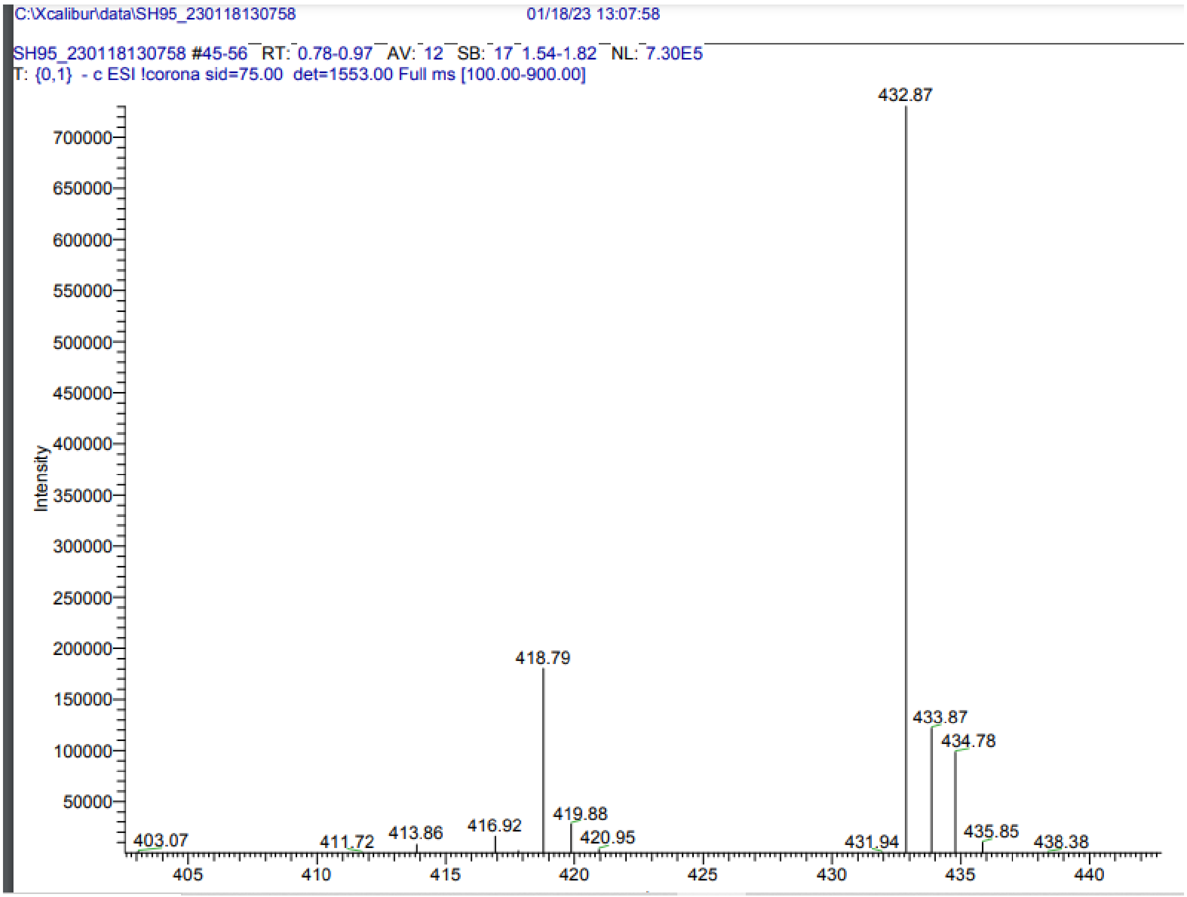

C:\Xcalibur\data\SH95_230118130758
01/18/23 13:07:58
SH95_230118130758 #45-56 RT: 0.78-0.97 AV: 12 SB: 17 1.54-1.82 NL: 7.30E5
T: {0,1} - c ESI !corona sid=75.00 det=1553.00 Full ms [100.00-900.00]
Intensity
432.87
418.79
433.87
434.78
419.88
420.95
416.92
413.86
411.72
403.07
431.94
435.85
438.38
405
410
415
420
425
430
435
440

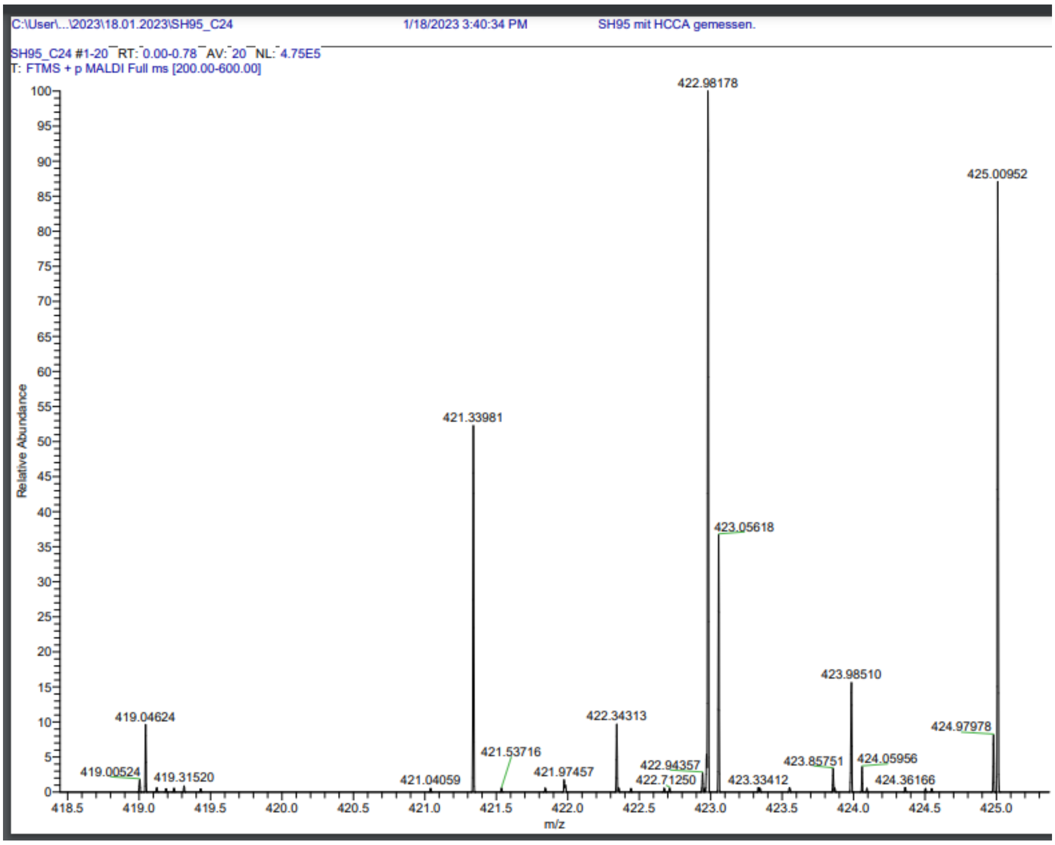
C:\User\...\2023\18.01.2023\SH95_C24
1/18/2023 3:40:34 PM
SH95 mit HCCA gemessen.
SH95_C24 #1-20 RT: 0.00-0.78 AV: 20 NL: 4.75E5
T: FTMS + p MALDI Full ms [200.00-600.00]
Relative Abundance
m/z
422.98178
425.00952
421.33981
423.05618
423.98510
419.04624
422.34313
424.97978
421.53716
421.97457
422.94357
423.85751
424.05956
419.00524
419.31520
421.04059
422.71250
423.33412
424.36166

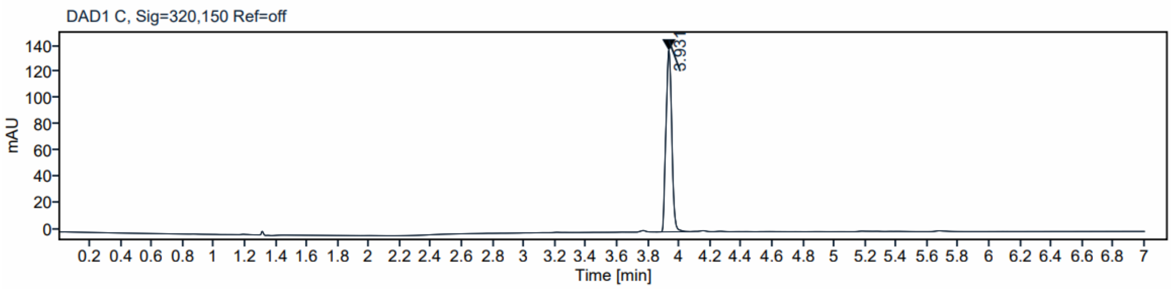
DAD1 C, Sig=320,150 Ref=off
mAU
Time [min]
3.931

^{1}H-, ^{13}C-, ^{19}F-NMR-, HRMS- and LC-ESI-MS-Spectrum of 2-(2-methylimidazo[1,2-b]pyridazin-6-yl)-7-[4-[3-[2,3,5,6-tetrafluoro-4-(2,3,4,5,6-pentafluorophenyl)phenyl]sulfanylpropanoyl]piperazin-1-yl]pyrido[1,2-a]pyrimidin-4-one **20**

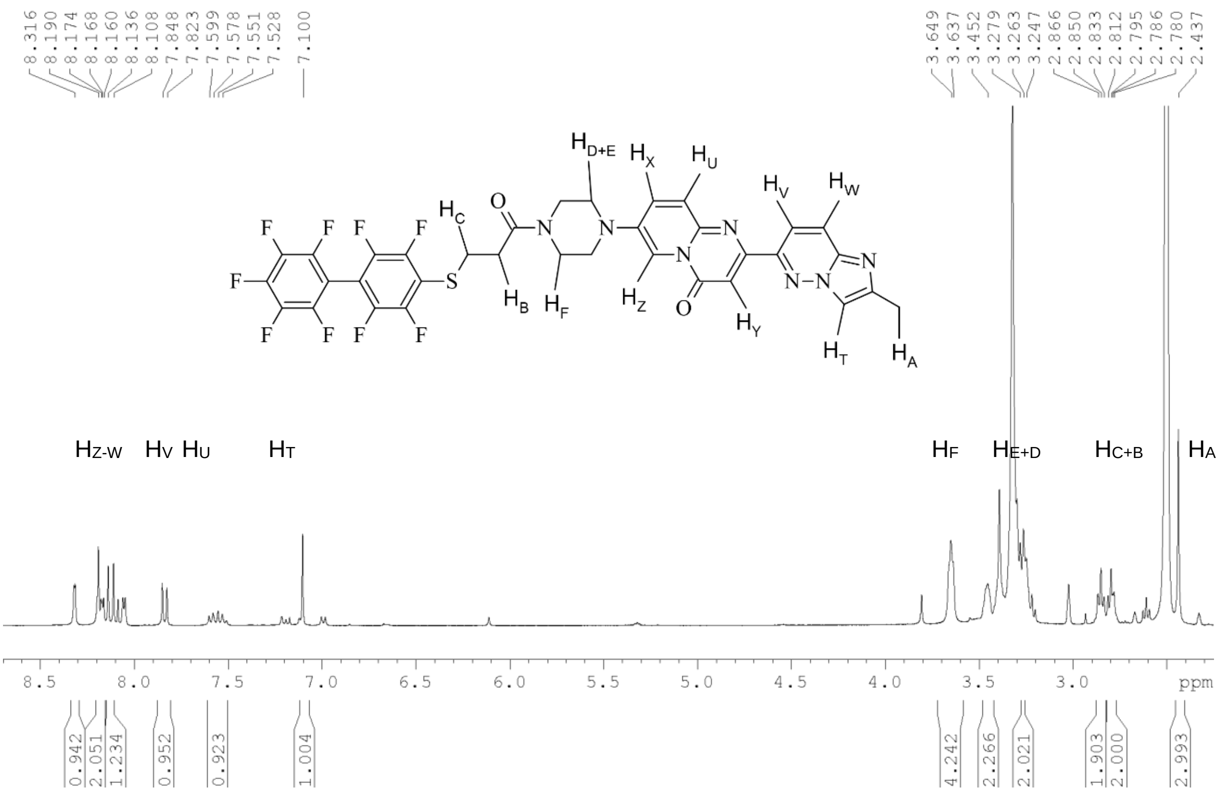

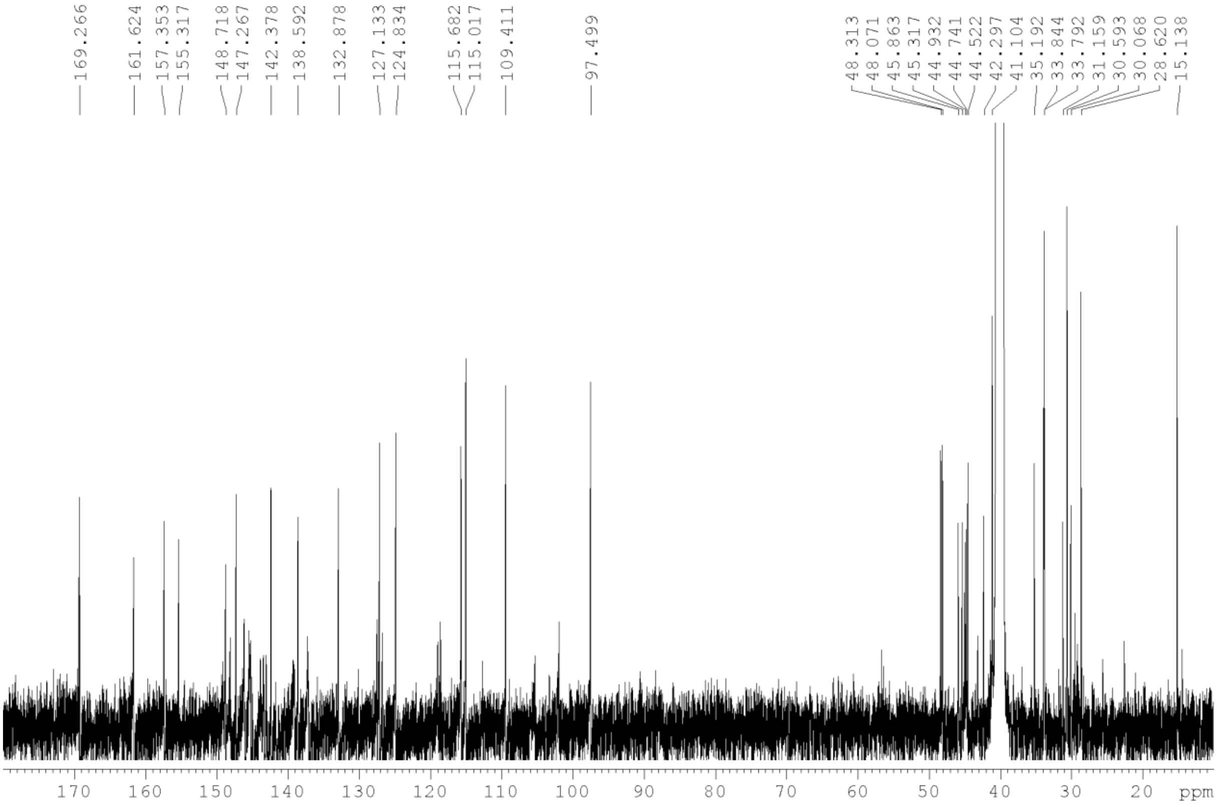

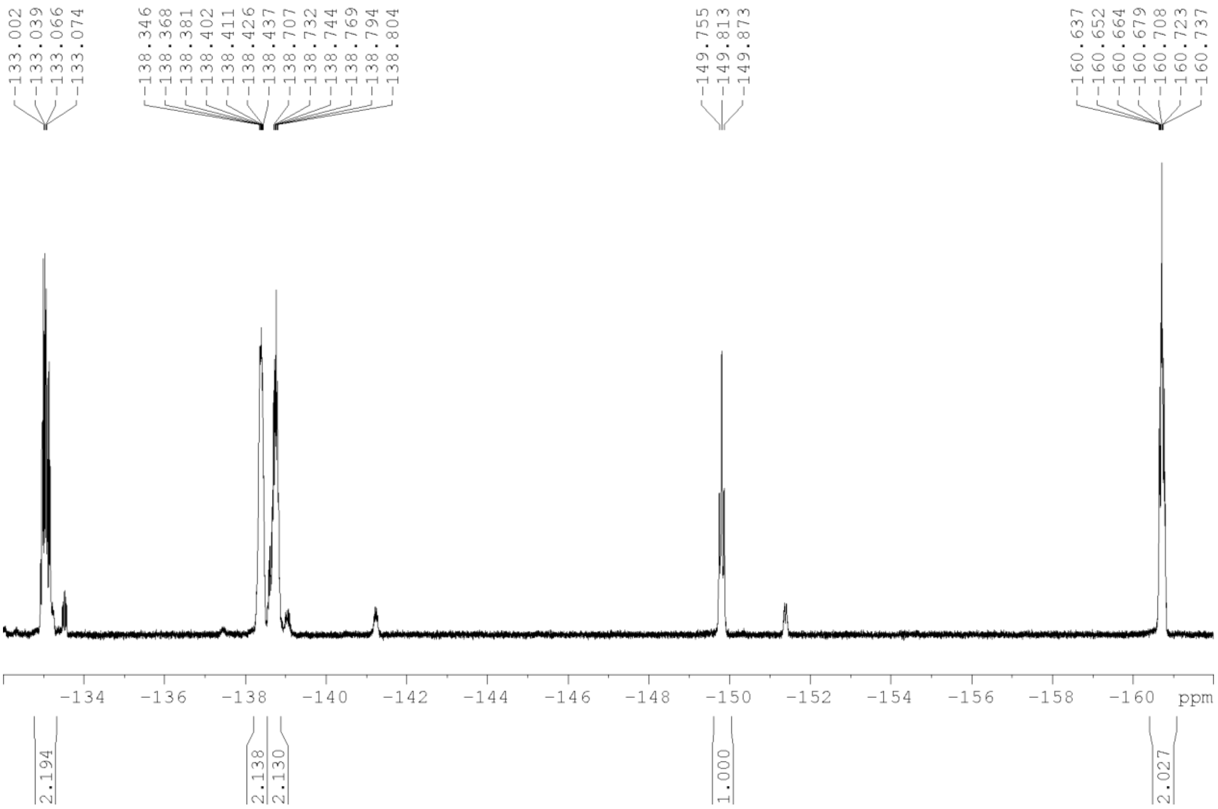
-133.002
-133.039
-133.066
-133.074
-138.346
-138.368
-138.381
-138.402
-138.411
-138.426
-138.437
-138.707
-138.732
-138.744
-138.769
-138.794
-138.804
-149.755
-149.813
-149.873
-160.637
-160.652
-160.664
-160.679
-160.708
-160.723
-160.737
-134 -136 -138 -140 -142 -144 -146 -148 -150 -152 -154 -156 -158 -160 ppm
2.194
2.138
2.130
1.000
2.027

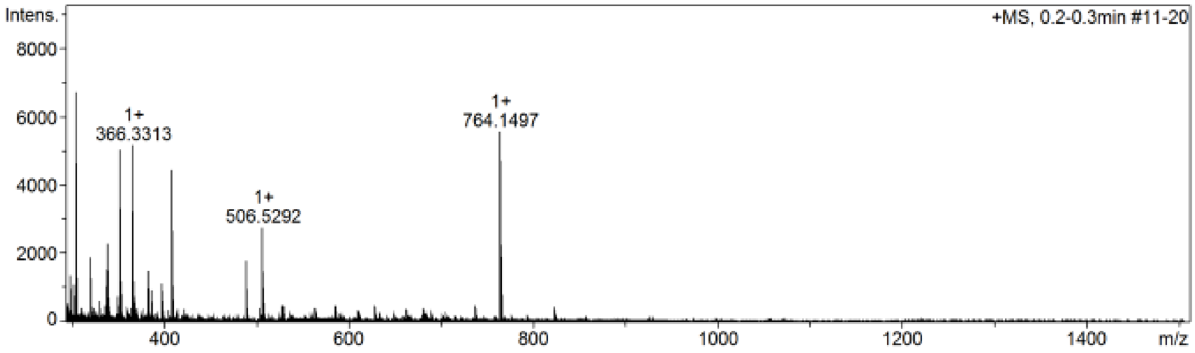
Intens.
+MS, 0.2-0.3min #11-20
1+
366.3313
1+
506.5292
1+
764.1497
0 2000 4000 6000 8000
400 600 800 1000 1200 1400 m/z

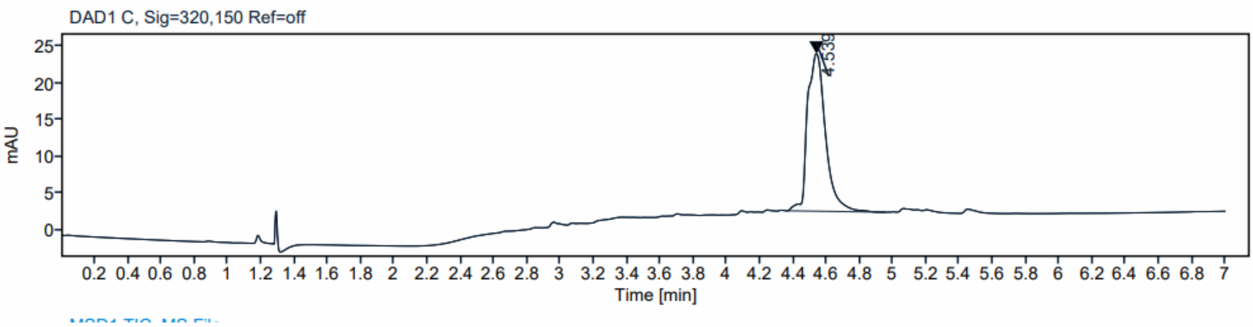
DAD1 C, Sig=320,150 Ref=off
mAU
4.539
Time [min]

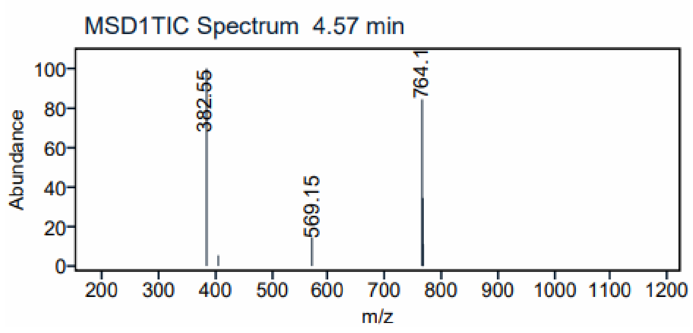
MSD1TIC Spectrum 4.57 min
Abundance
100
80
60
40
20
0
382.55
569.15
764.1
200
300
400
500
600
700
800
900
1000
1100
1200
m/z

^{1}H-, ^{19}F-, ESI-, MALDI-, and LC-MS-spectrum of 2-[2,3,5,6-Tetrafluoro-4-(2,3,4,5,6-pentafluoro-phenyl)phenyl]sulfanylethanamine **21**

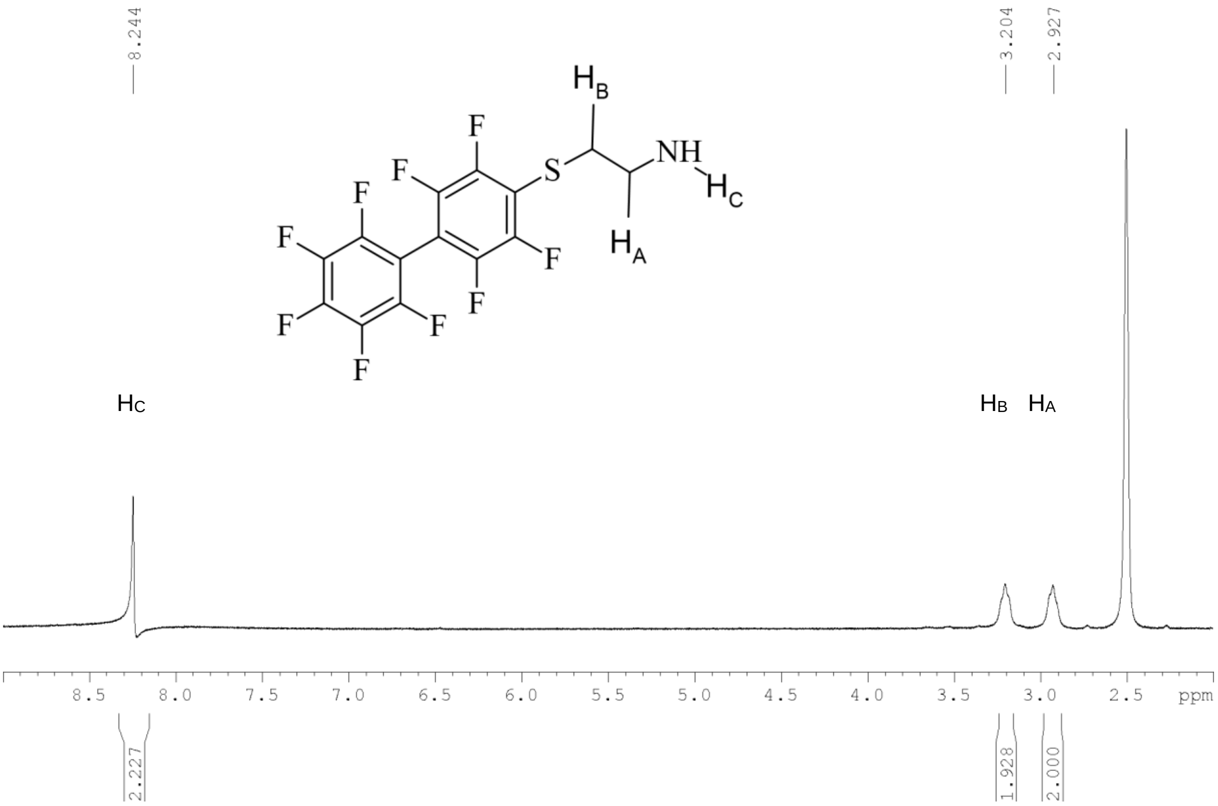

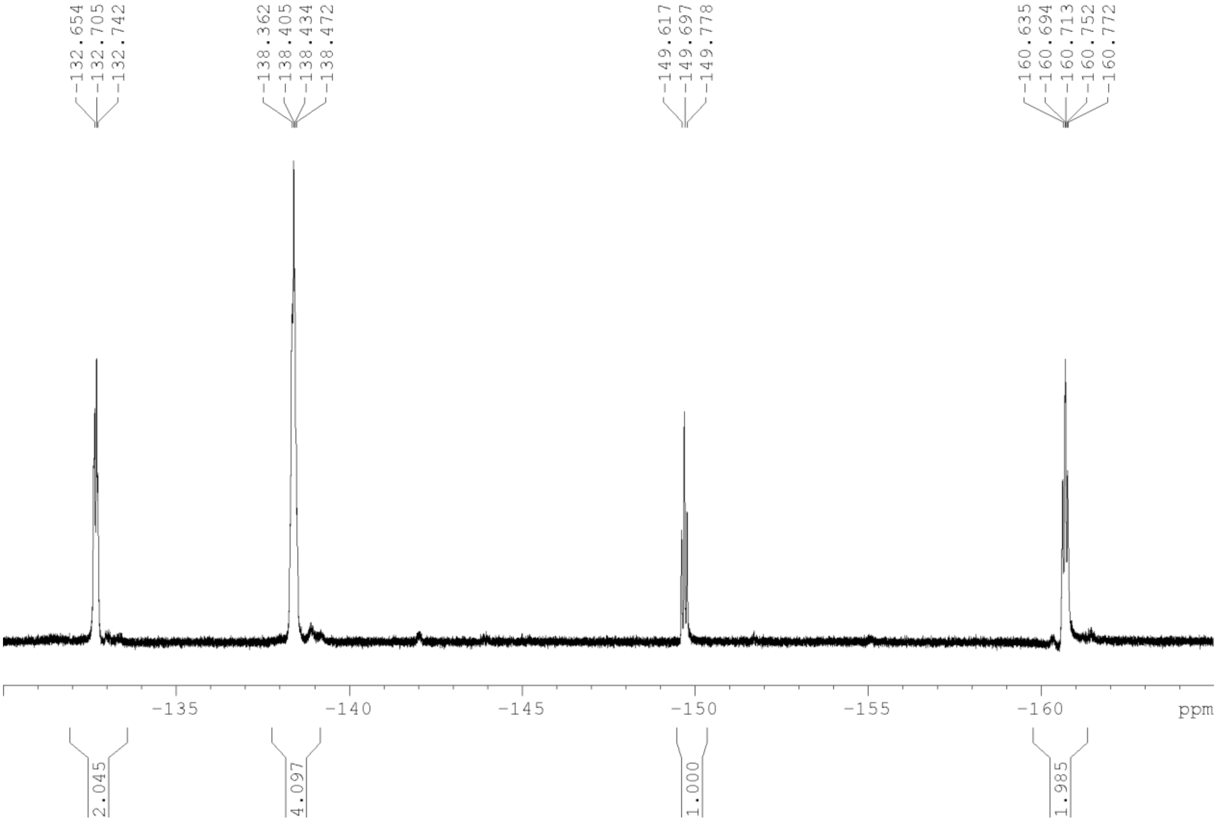

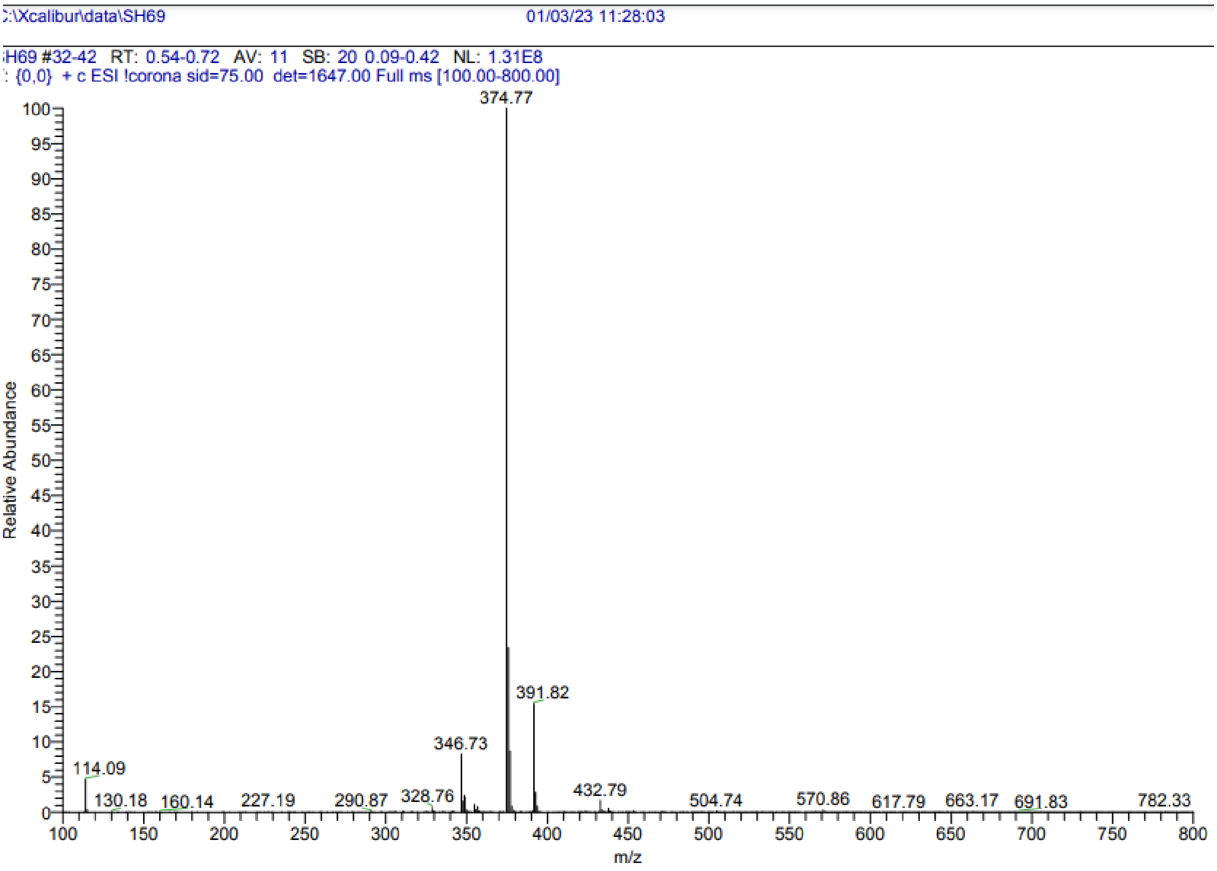
:\Xcalibur\data\SH69
01/03/23 11:28:03
H69 #32-42 RT: 0.54-0.72 AV: 11 SB: 20 0.09-0.42 NL: 1.31E8
: {0,0} + c ESI !corona sid=75.00 det=1647.00 Full ms [100.00-800.00]
374.77
391.82
346.73
114.09
130.18
160.14
227.19
290.87
328.76
432.79
504.74
570.86
617.79
663.17
691.83
782.33
Relative Abundance
m/z

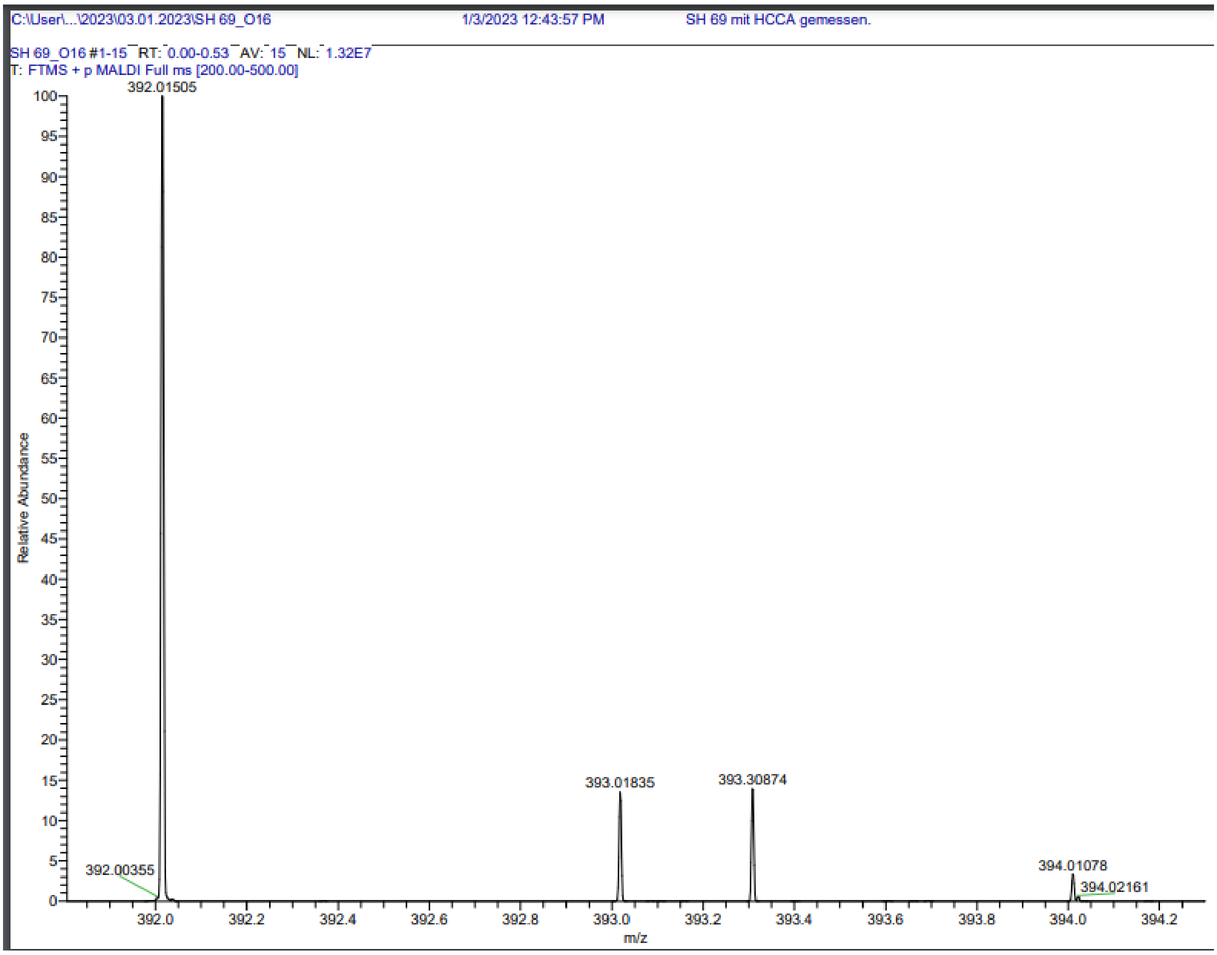
C:\User\...\2023\03.01.2023\SH 69_O16
1/3/2023 12:43:57 PM
SH 69 mit HCCA gemessen.
SH 69_O16 #1-15 RT: 0.00-0.53 AV: 15 NL: 1.32E7
T: FTMS + p MALDI Full ms [200.00-500.00]
392.01505
392.00355
393.01835
393.30874
394.01078
394.02161
Relative Abundance
m/z

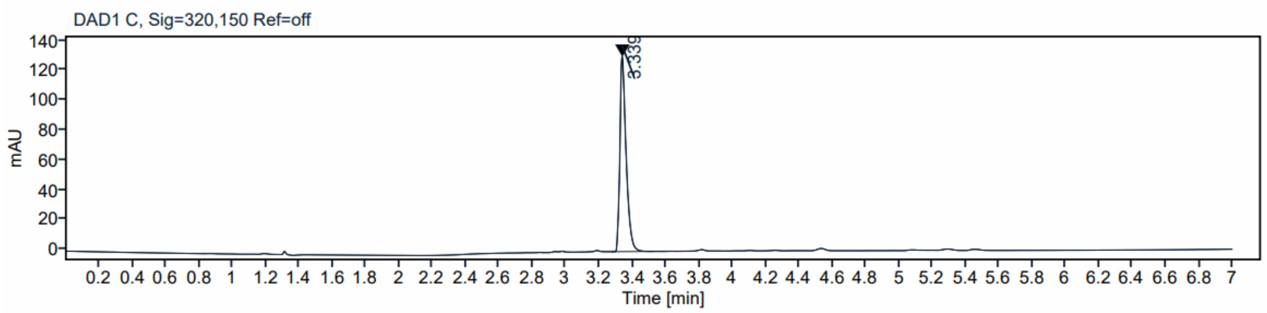

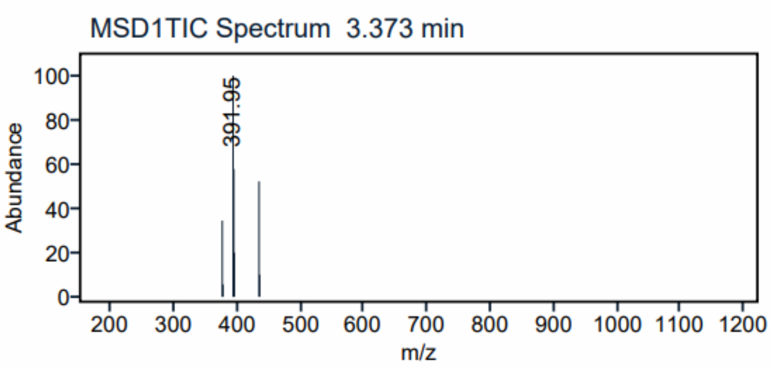

<u>^{1}H-, ^{19}F-NMR-, HRMS- and HPLC-spectrum of *tert*-butyl 4-oxo-4-[2-[2,3,5,6-tetrafluoro-4-(2,3,4,5,6-pentafluorophenyl)phenyl]sulfanylethylamino]butanoat and ^{1}H-, ^{13}C-, ^{19}F-NMR-, HRMS- and LC-MS-ESI-spectrum of 4-oxo-4-[2-[2,3,5,6-tetrafluoro-4-(2,3,4,5,6-pentafluoro-phenyl)phenyl]sulfanylethylamino]butanic acid **23 a**</u>

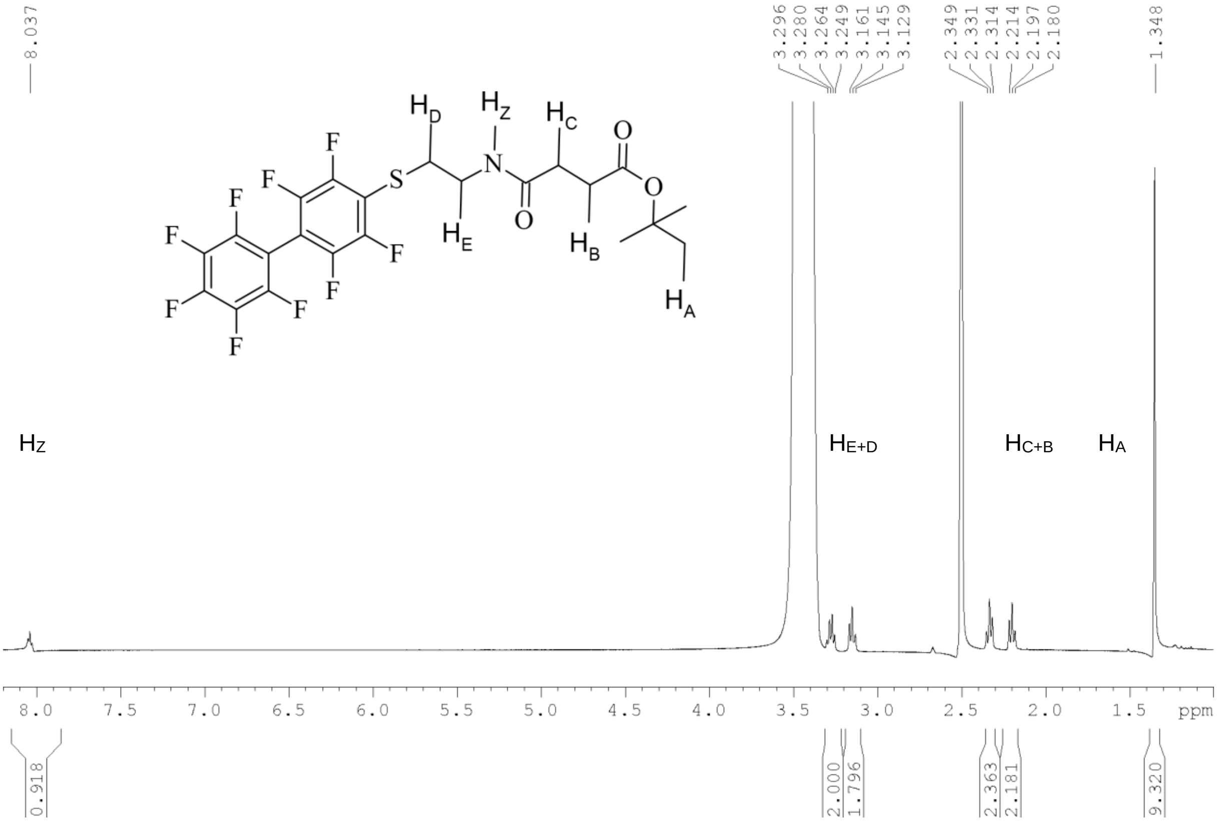

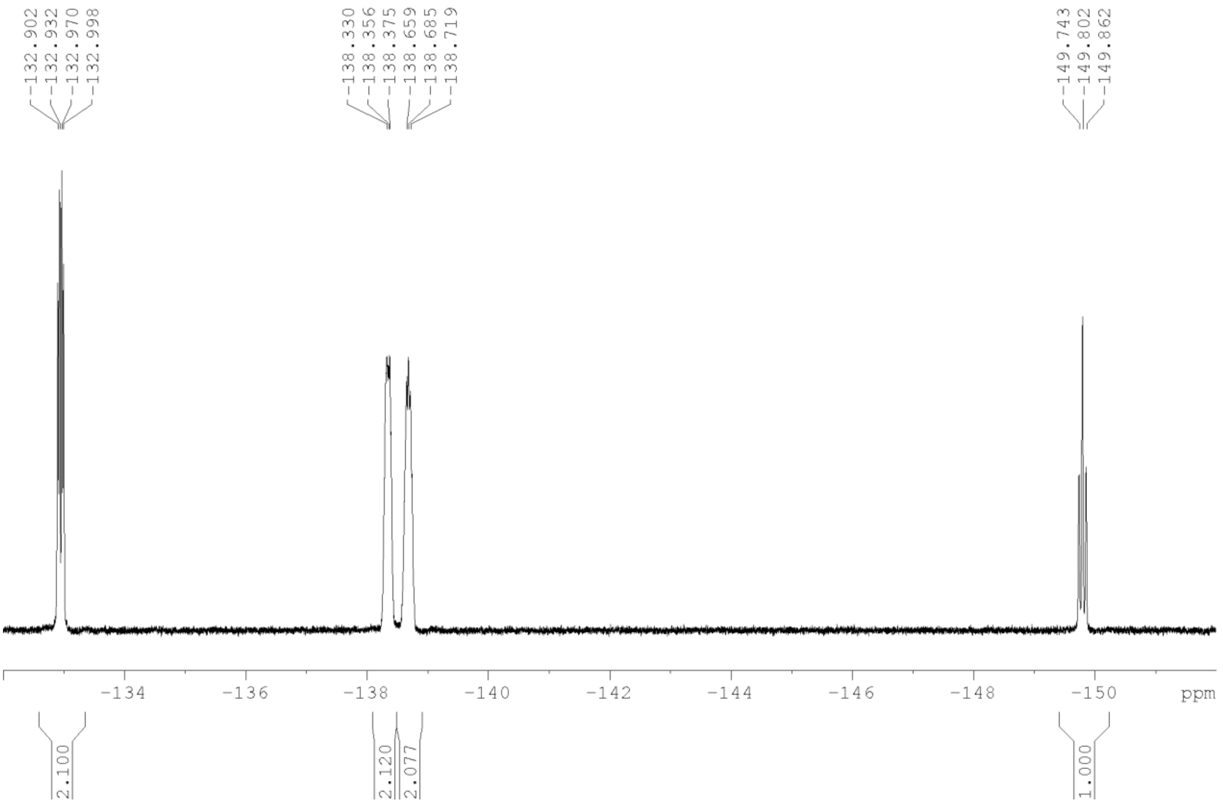
-132.902
-132.932
-132.970
-132.998
-138.330
-138.356
-138.375
-138.659
-138.685
-138.719
-149.743
-149.802
-149.862
-134 -136 -138 -140 -142 -144 -146 -148 -150 ppm
2.100
2.120
2.077
1.000

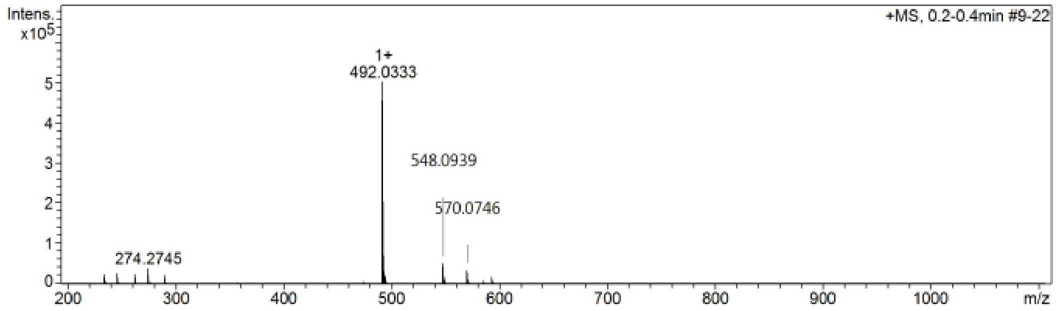
Intens.
x10^5
+MS, 0.2-0.4min #9-22
1+
492.0333
548.0939
570.0746
274.2745
m/z

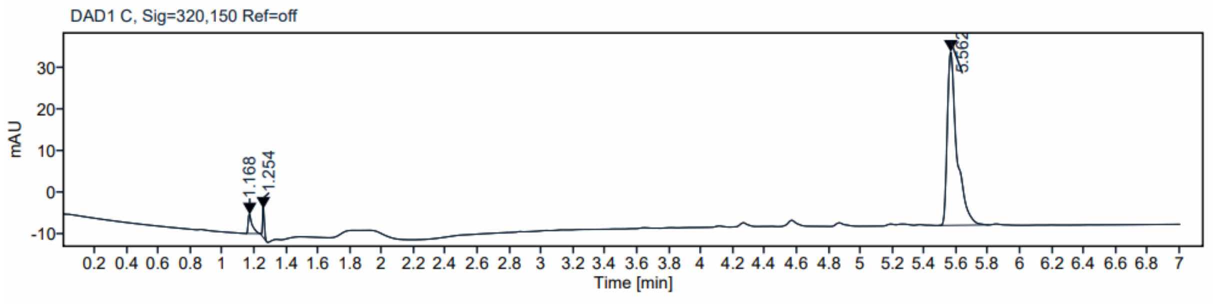
DAD1 C, Sig=320,150 Ref=off
mAU
1.168
1.254
5.562
Time [min]

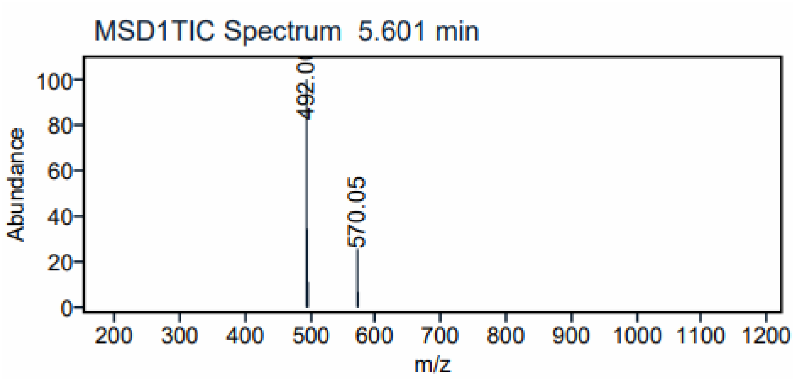
MSD1TIC Spectrum 5.601 min
Abundance
m/z
492.0
570.05

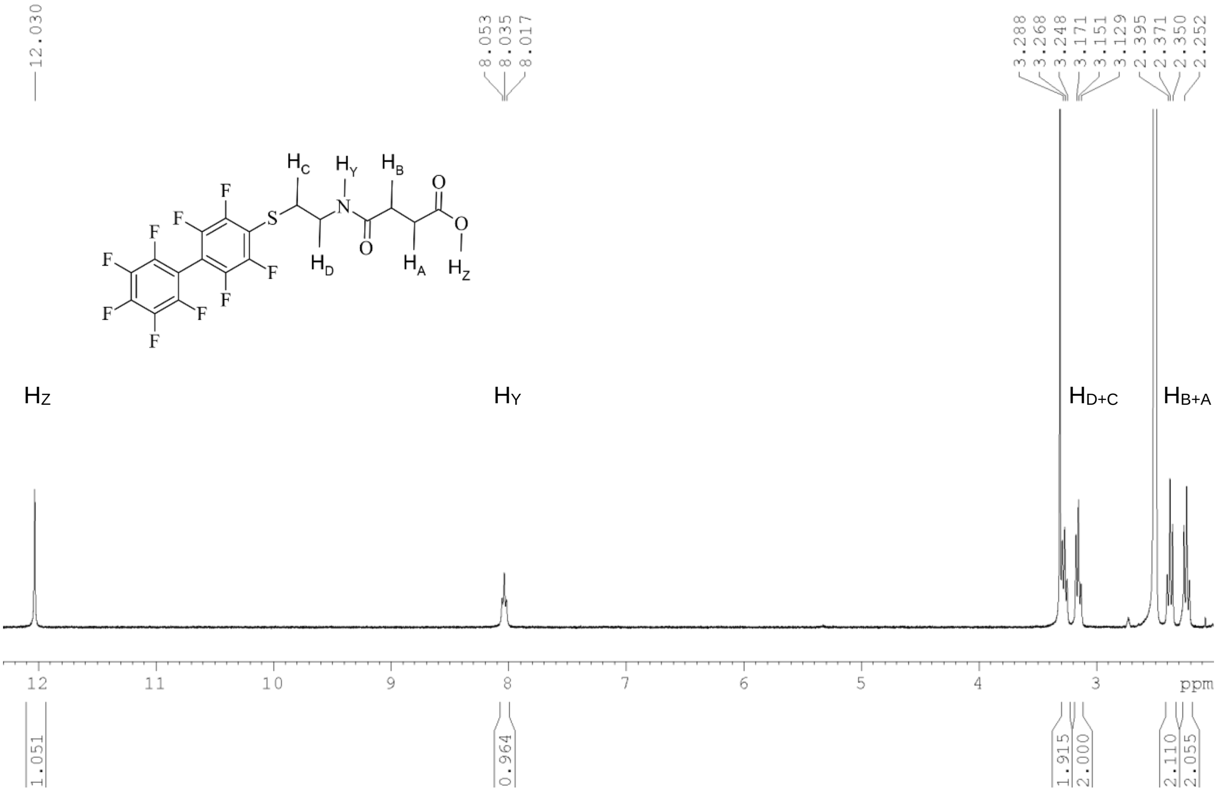
HZ
HY
HD+C
HB+A
ppm

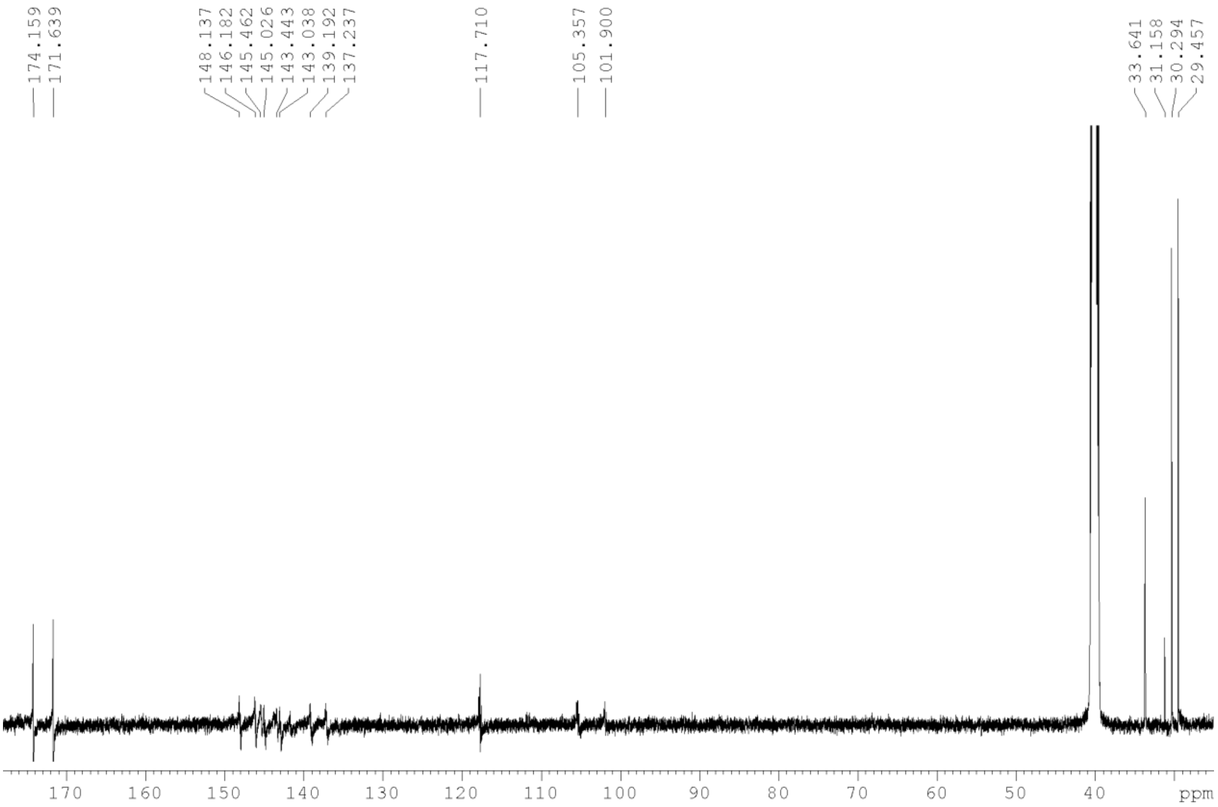
174.159
171.639
148.137
146.182
145.462
145.026
143.443
143.038
139.192
137.237
117.710
105.357
101.900
33.641
31.158
30.294
29.457
170 160 150 140 130 120 110 100 90 80 70 60 50 40 ppm

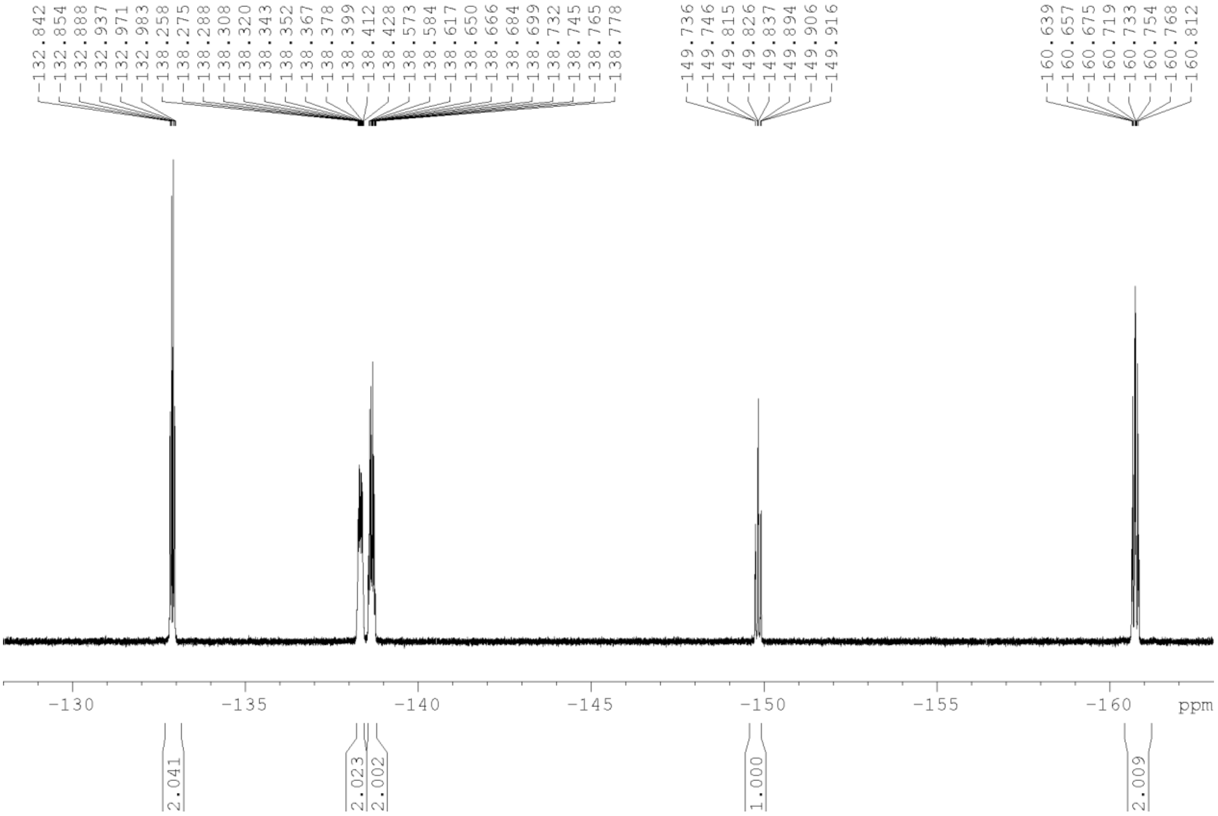
-132.842
-132.854
-132.888
-132.937
-132.971
-132.983
-138.258
-138.275
-138.288
-138.308
-138.320
-138.343
-138.352
-138.367
-138.378
-138.399
-138.412
-138.428
-138.573
-138.584
-138.617
-138.650
-138.666
-138.684
-138.699
-138.732
-138.745
-138.765
-138.778
-149.736
-149.746
-149.815
-149.826
-149.837
-149.894
-149.906
-149.916
-160.639
-160.657
-160.675
-160.719
-160.733
-160.754
-160.768
-160.812
-130 -135 -140 -145 -150 -155 -160 ppm
2.041
2.023
2.002
1.000
2.009

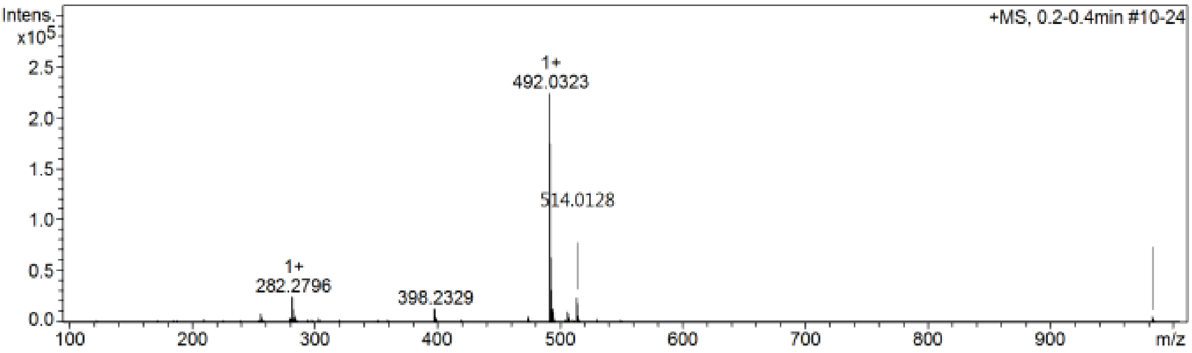

Intens.
x10^5
+MS, 0.2-0.4min #10-24
1+
492.0323
514.0128
1+
282.2796
398.2329
m/z

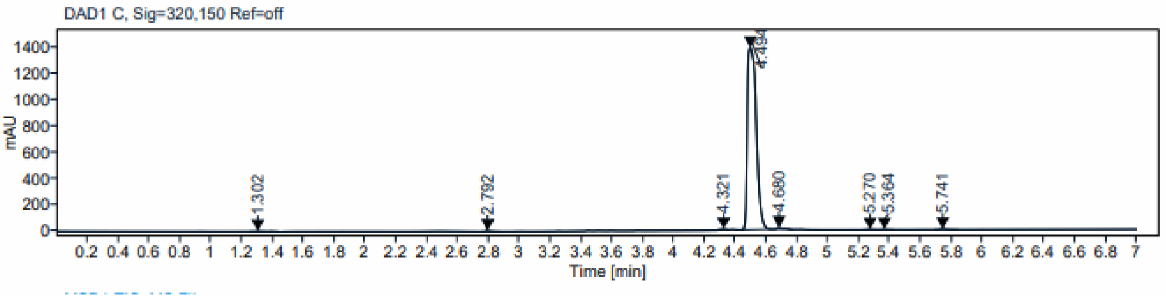

DAD1 C, Sig=320,150 Ref=off
mAU
1.302
2.792
4.321
4.494
4.680
5.270
5.364
5.741
Time [min]

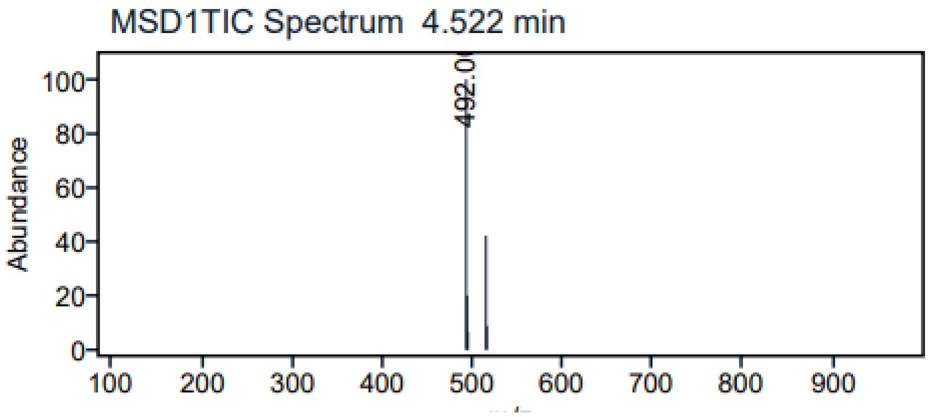

MSD1TIC Spectrum 4.522 min
Abundance
492.0

^{1}H-, ^{13}C-, ^{19}F-NMR-, HRMS- and LC-ESI-MS-spectrum of 4-[4-[2-(2-Methylimidazo[1,2-b]pyridazin-6-yl)-4-oxo-pyrido[1,2-a]pyrimidin-7-yl]piperazin-1-yl]-4-oxo-*N*-[2-[2,3,5,6-tetrafluoro-4-(2,3,4,5,6-pentafluorophenyl)phenyl]sulfanylethyl]butanamide **24 a**

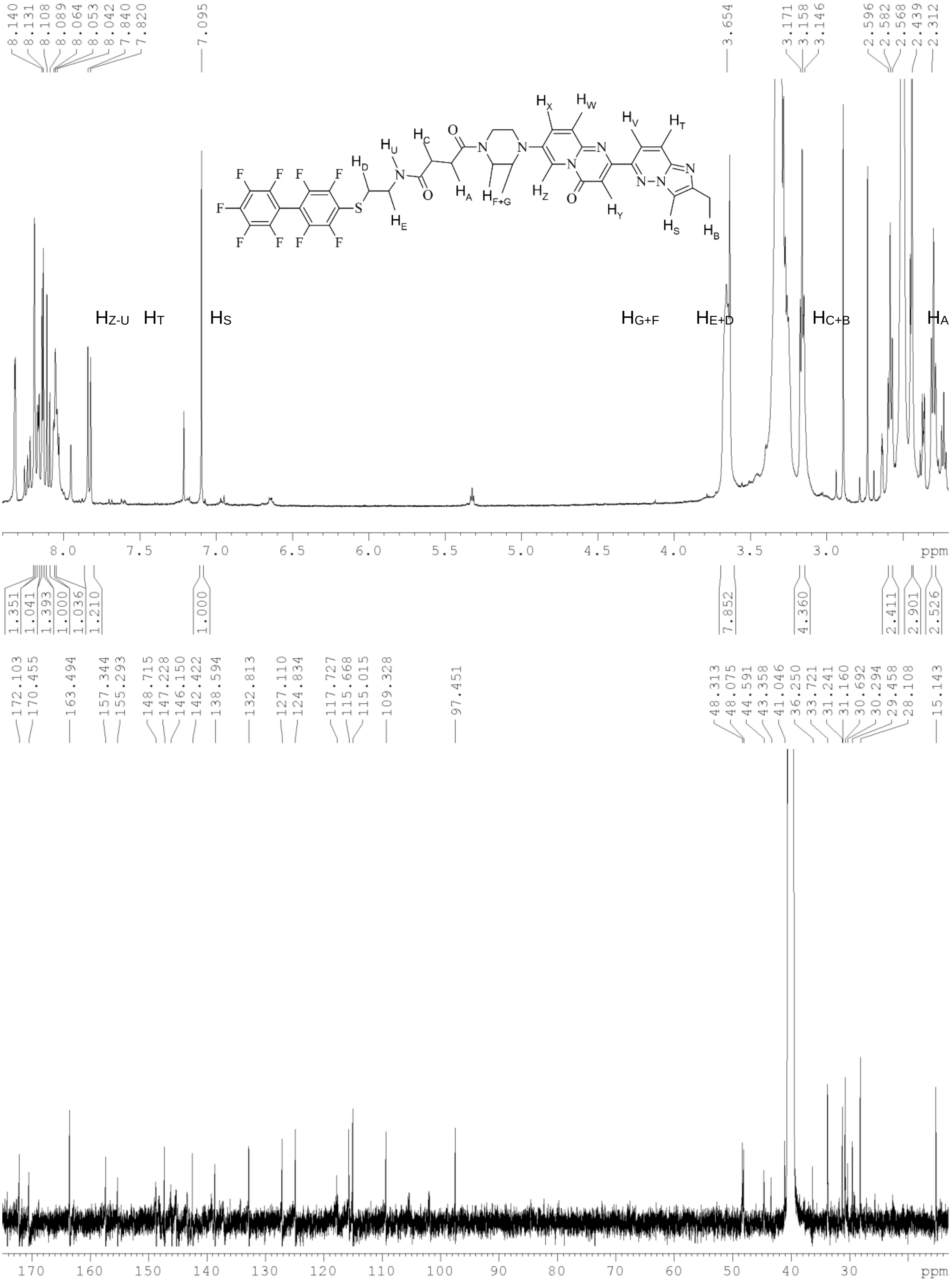

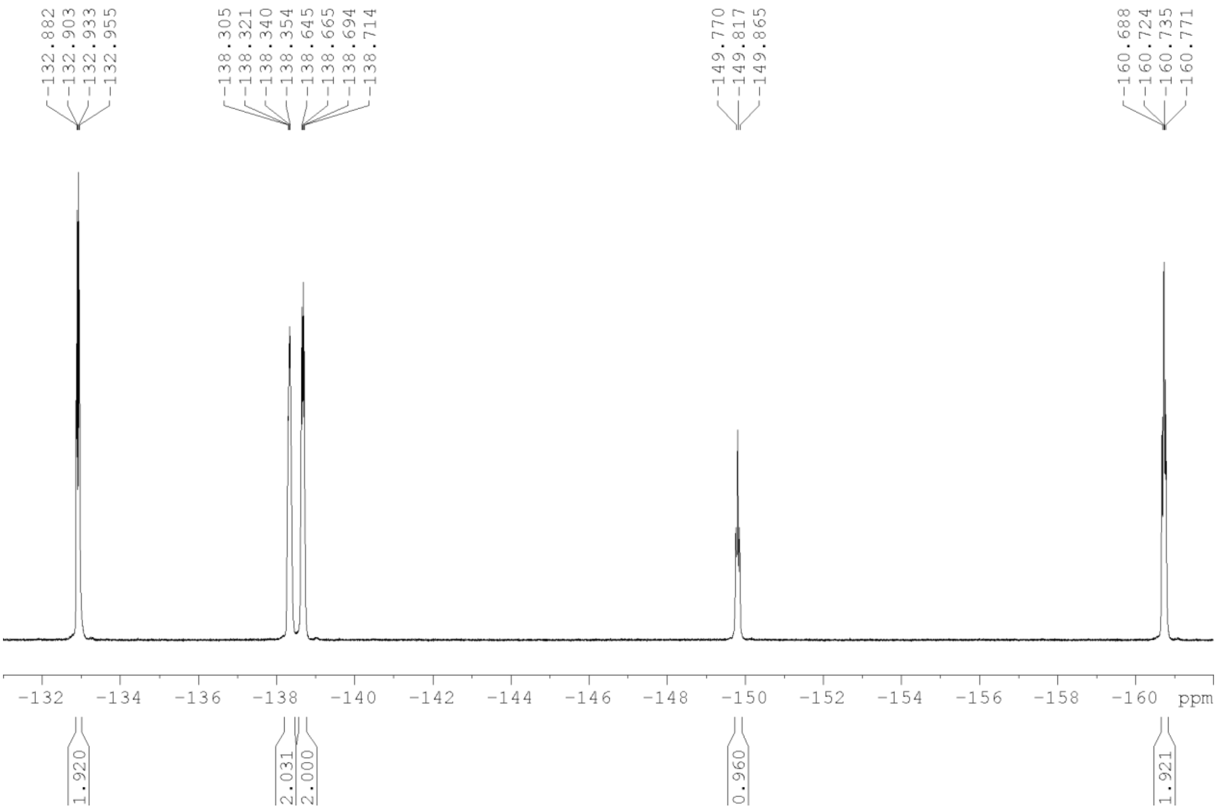

-132.882
-132.903
-132.933
-132.955
-138.305
-138.321
-138.340
-138.354
-138.645
-138.665
-138.694
-138.714
-149.770
-149.817
-149.865
-160.688
-160.724
-160.735
-160.771
-132 -134 -136 -138 -140 -142 -144 -146 -148 -150 -152 -154 -156 -158 -160 ppm
1.920
2.031
2.000
0.960
1.921

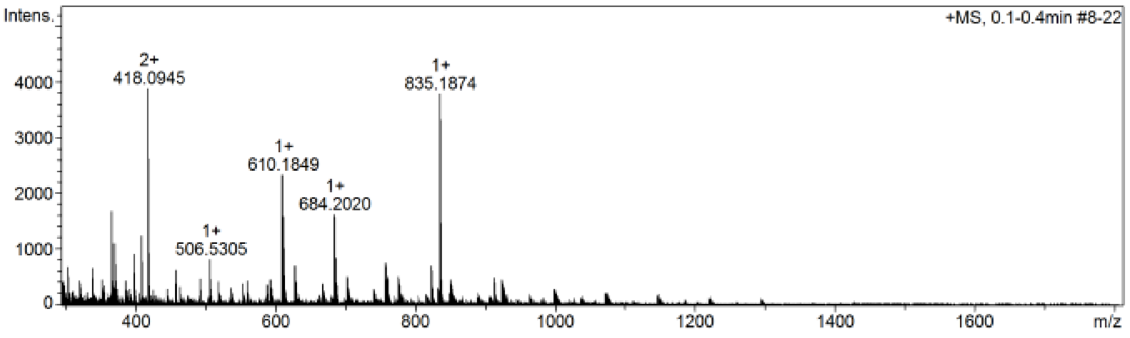

Intens.
+MS, 0.1-0.4min #8-22
2+
418.0945
1+
835.1874
1+
610.1849
1+
684.2020
1+
506.5305
4000
3000
2000
1000
0
400
600
800
1000
1200
1400
1600
m/z

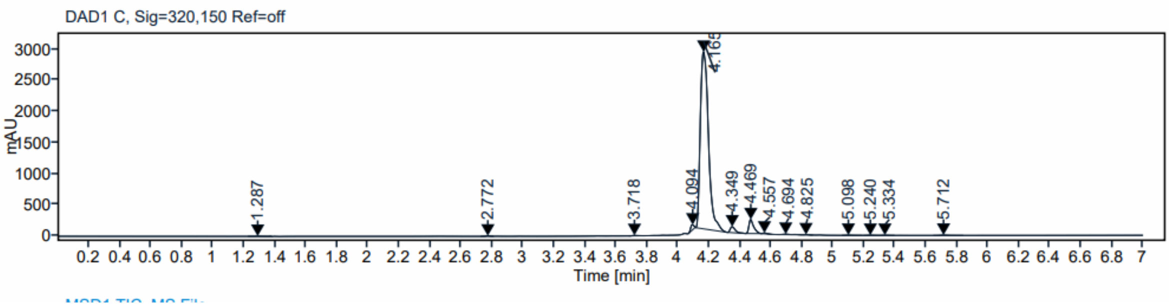

DAD1 C, Sig=320,150 Ref=off
mAU
1.287
2.772
3.718
4.094
4.349
4.469
4.557
4.694
4.825
5.098
5.240
5.334
5.712
Time [min]

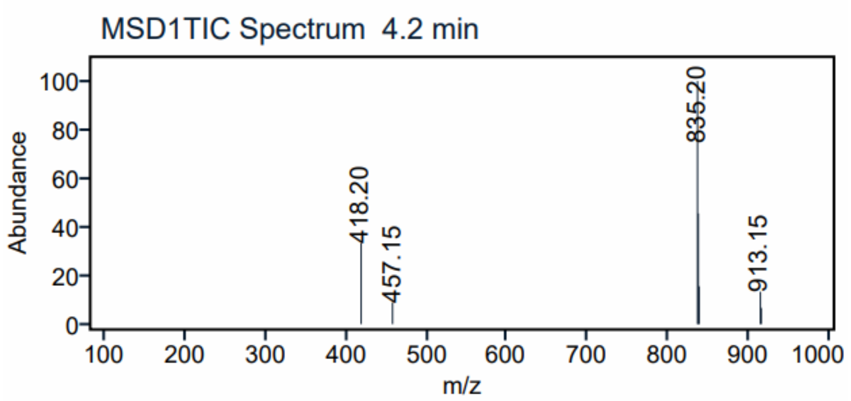

<u>^{1}H-, ^{19}F-NMR-, HRMS- and LC-MS-Spectrum of *tert*-butyl 6-oxo-6-[2-[2,3,5,6-tetrafluoro-4-(2,3,4,5,6-pentafluorophenyl)phenyl]sulfanylethylamino]hexanoate and ^{1}H-, ^{13}C-, ^{19}F-NMR-, HRMS- and LC-MS-spectrum of 6-oxo-6-[2-[2,3,5,6-tetrafluoro-4-(2,3,4,5,6-pentafluoro-phenyl)phenyl]sulfanylethylamino]hexanic acid **23 b**</u>

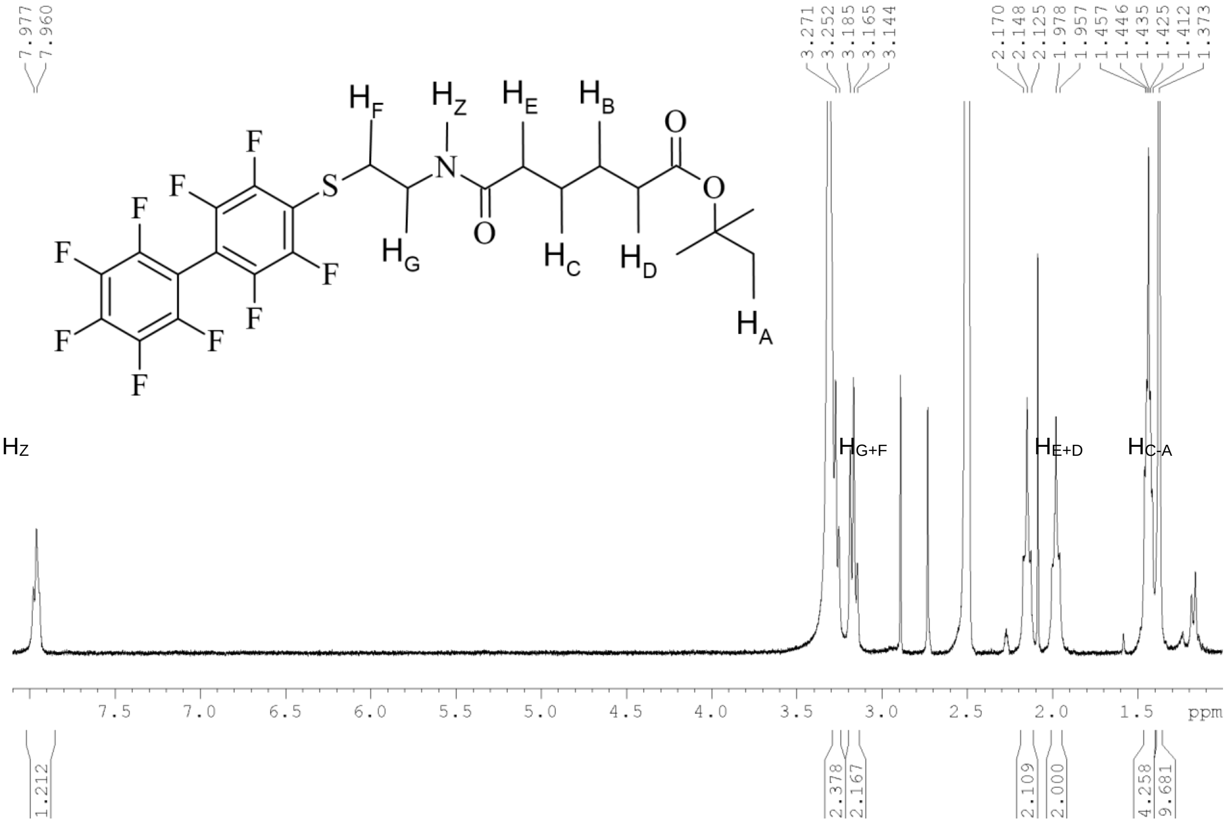

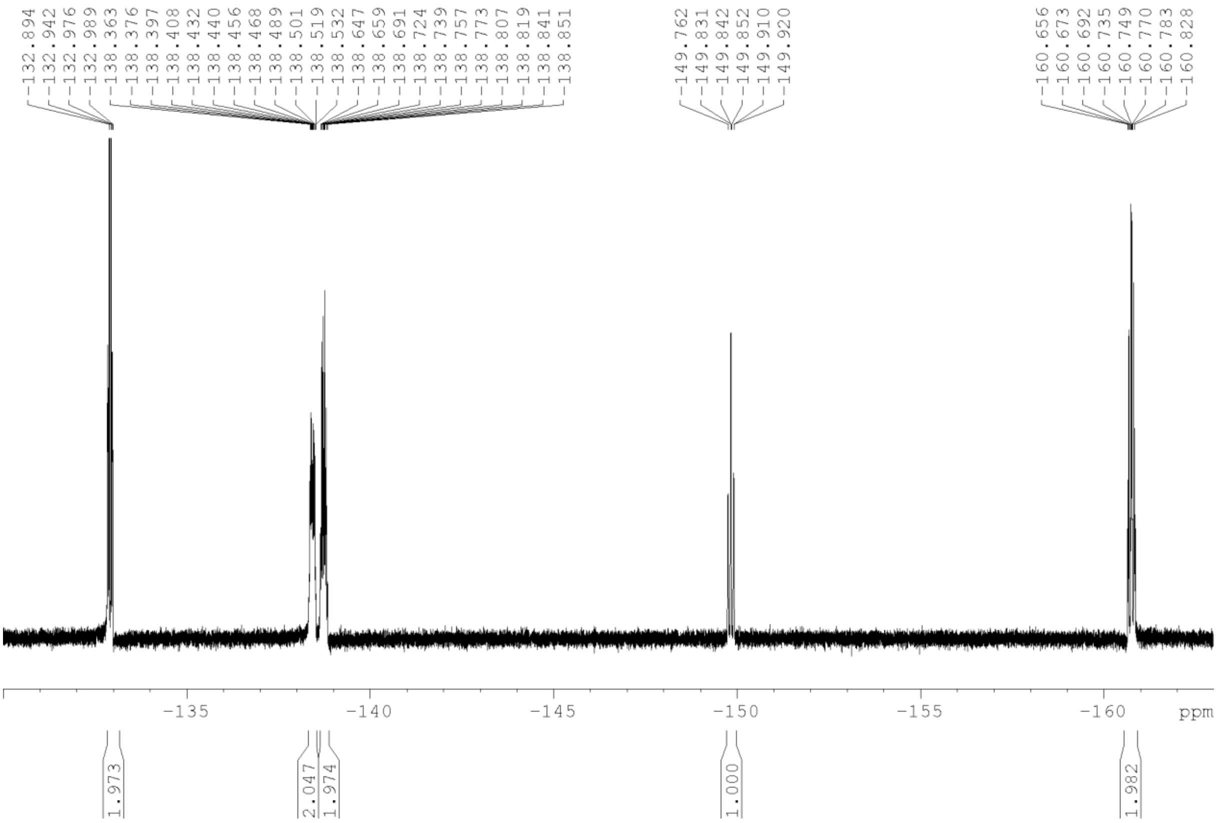
-132.894
-132.942
-132.976
-132.989
-138.363
-138.376
-138.397
-138.408
-138.432
-138.440
-138.456
-138.468
-138.489
-138.501
-138.519
-138.532
-138.647
-138.659
-138.691
-138.724
-138.739
-138.757
-138.773
-138.807
-138.819
-138.841
-138.851
-149.762
-149.831
-149.842
-149.852
-149.910
-149.920
-160.656
-160.673
-160.692
-160.735
-160.749
-160.770
-160.783
-160.828
-135
-140
-145
-150
-155
-160
ppm
1.973
2.047
1.974
1.000
1.982

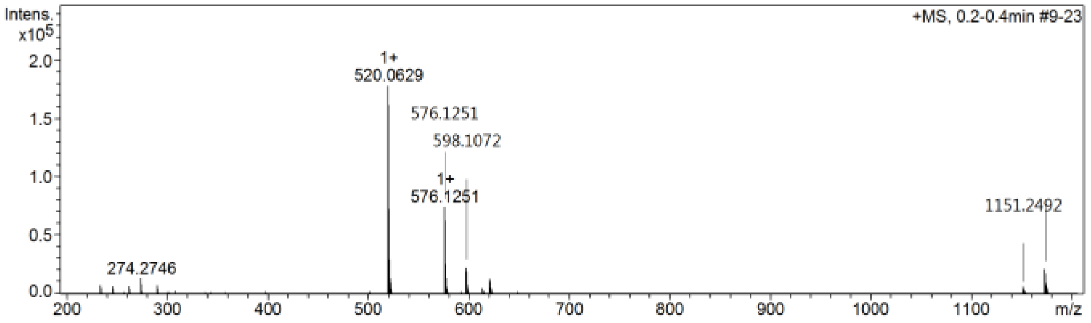
Intens.
x10^5
+MS, 0.2-0.4min #9-23
1+
520.0629
576.1251
598.1072
1+
576.1251
1151.2492
274.2746
m/z

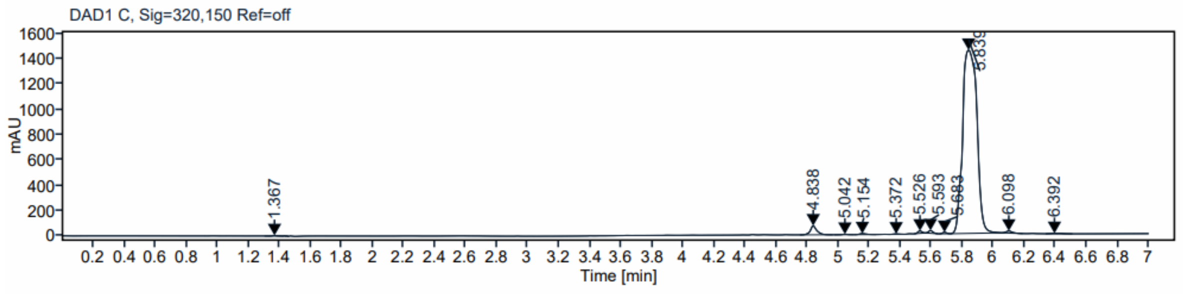
DAD1 C, Sig=320,150 Ref=off
mAU
1.367
4.838
5.042
5.154
5.372
5.526
5.593
5.683
5.839
6.098
6.392
Time [min]

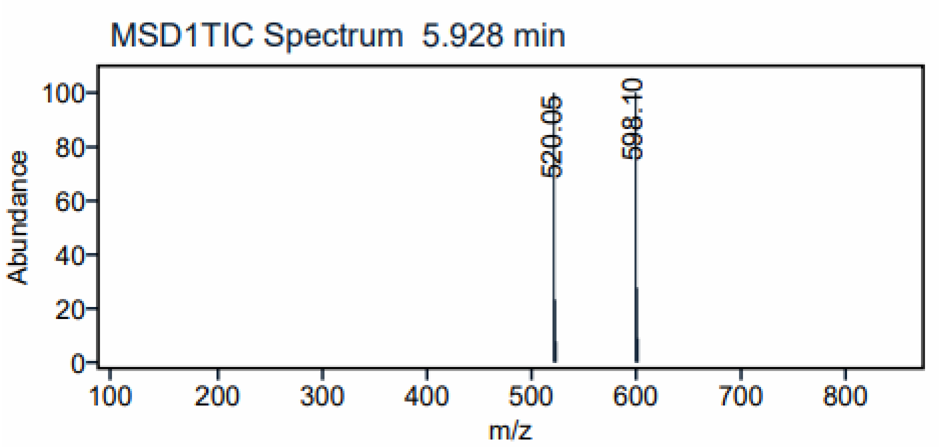
MSD1TIC Spectrum 5.928 min
Abundance
m/z
520.05
598.10

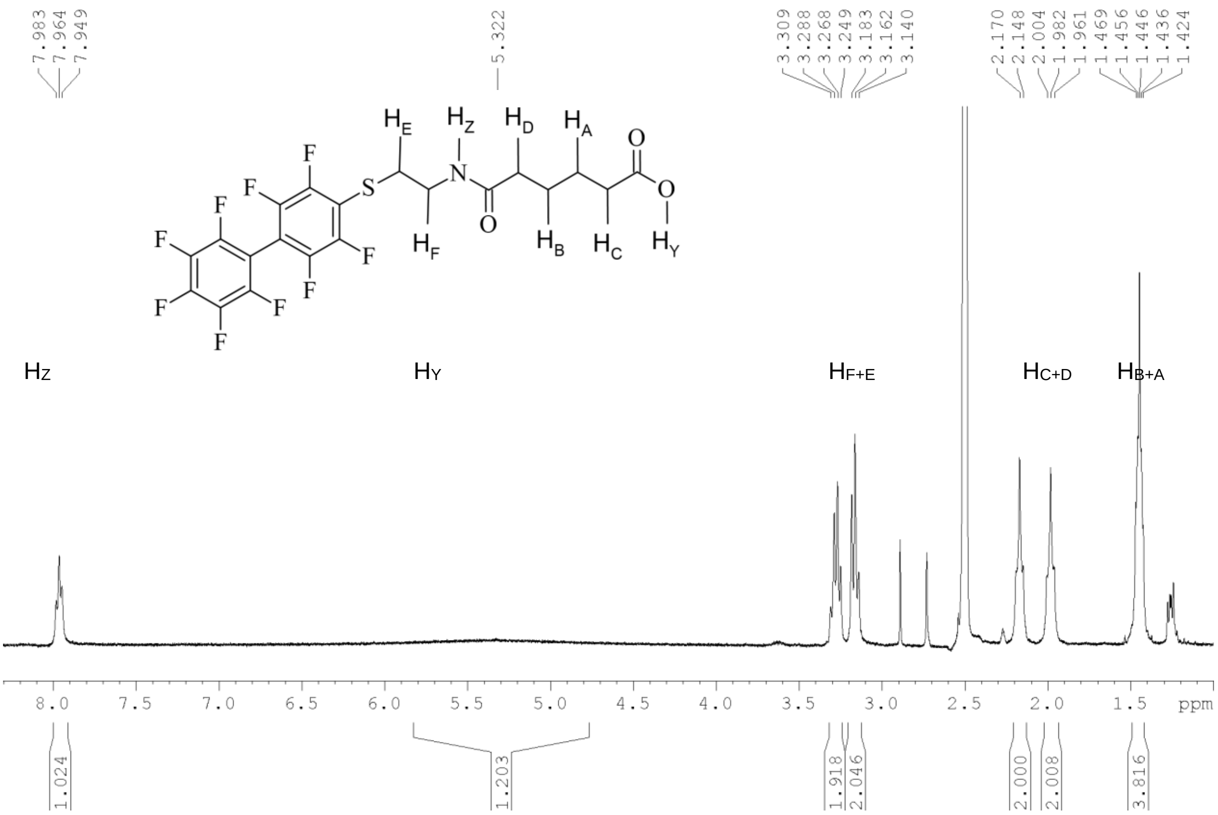
HZ
HY
HF+E
HC+D
HB+A
ppm

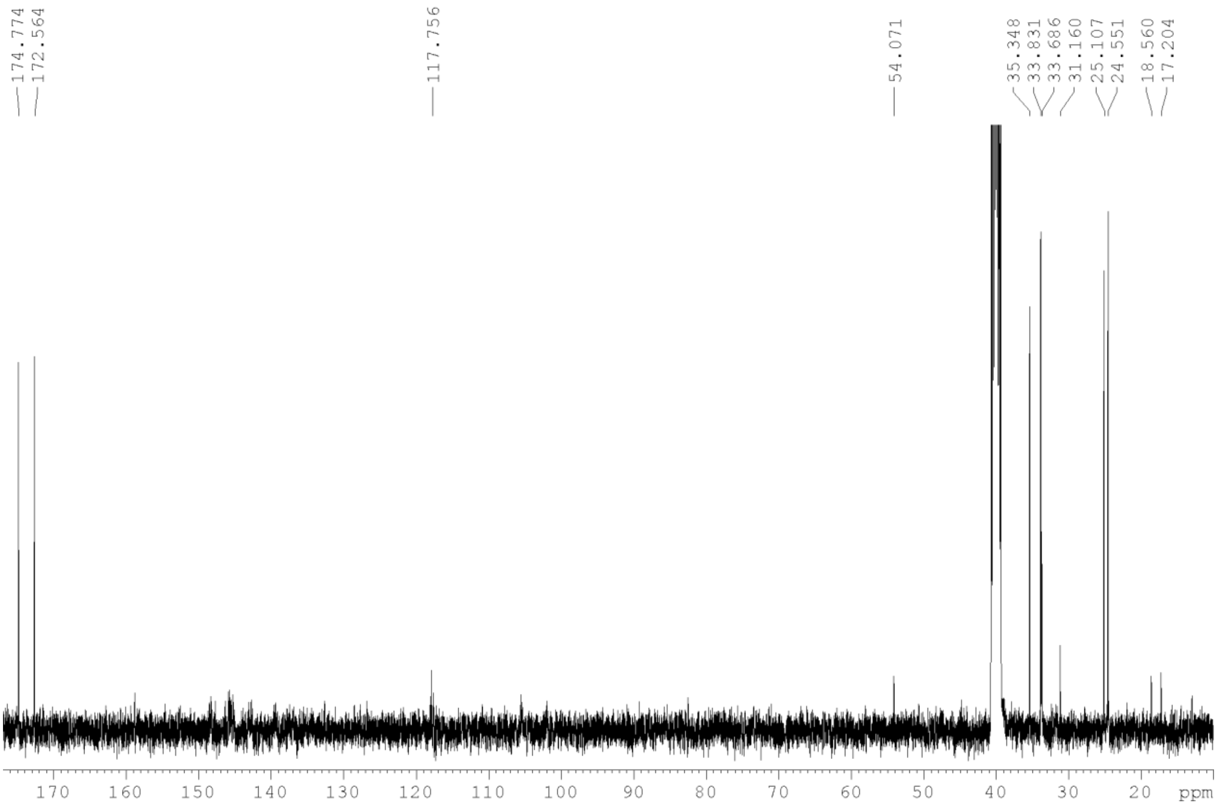
174.774
172.564
117.756
54.071
35.348
33.831
33.686
31.160
25.107
24.551
18.560
17.204
170 160 150 140 130 120 110 100 90 80 70 60 50 40 30 20 ppm

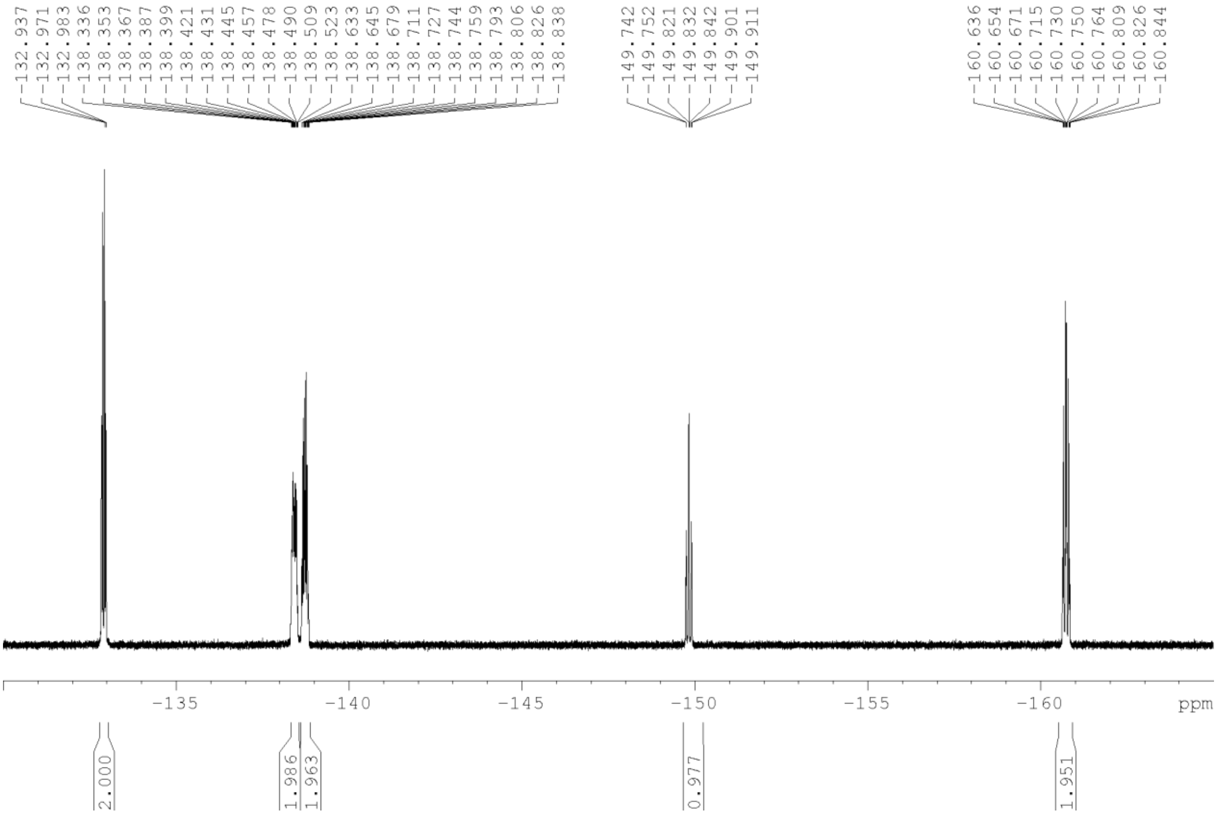
-132.937
-132.971
-132.983
-138.336
-138.353
-138.367
-138.387
-138.399
-138.421
-138.431
-138.445
-138.457
-138.478
-138.490
-138.509
-138.523
-138.633
-138.645
-138.679
-138.711
-138.727
-138.744
-138.759
-138.793
-138.806
-138.826
-138.838
-149.742
-149.752
-149.821
-149.832
-149.842
-149.901
-149.911
-160.636
-160.654
-160.671
-160.715
-160.730
-160.750
-160.764
-160.809
-160.826
-160.844
-135 -140 -145 -150 -155 -160 ppm
2.000
1.986
1.963
0.977
1.951

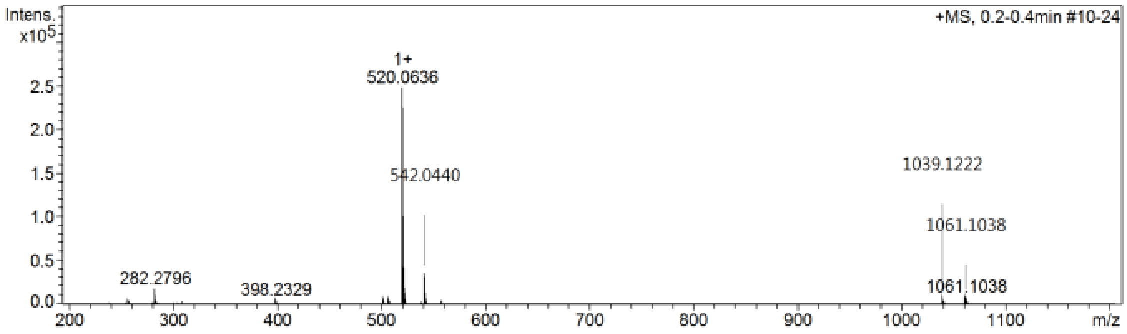
Intens.
x10^5
+MS, 0.2-0.4min #10-24
1+
520.0636
542.0440
1039.1222
1061.1038
282.2796
398.2329
1061.1038
m/z

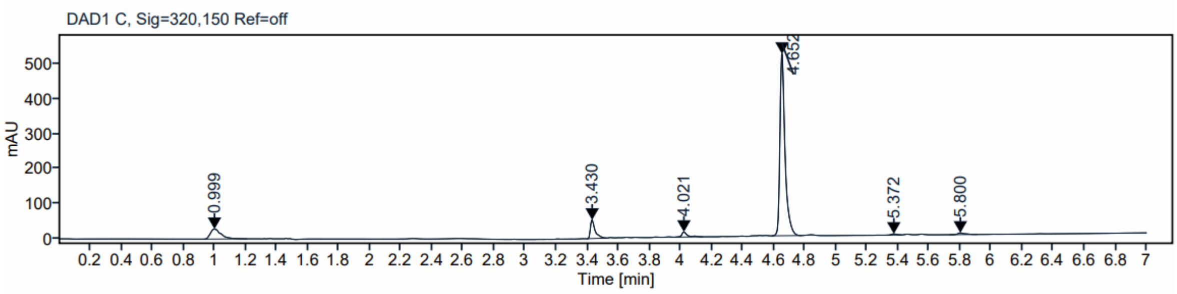
DAD1 C, Sig=320,150 Ref=off
mAU
0.999
3.430
4.021
4.652
5.372
5.800
Time [min]

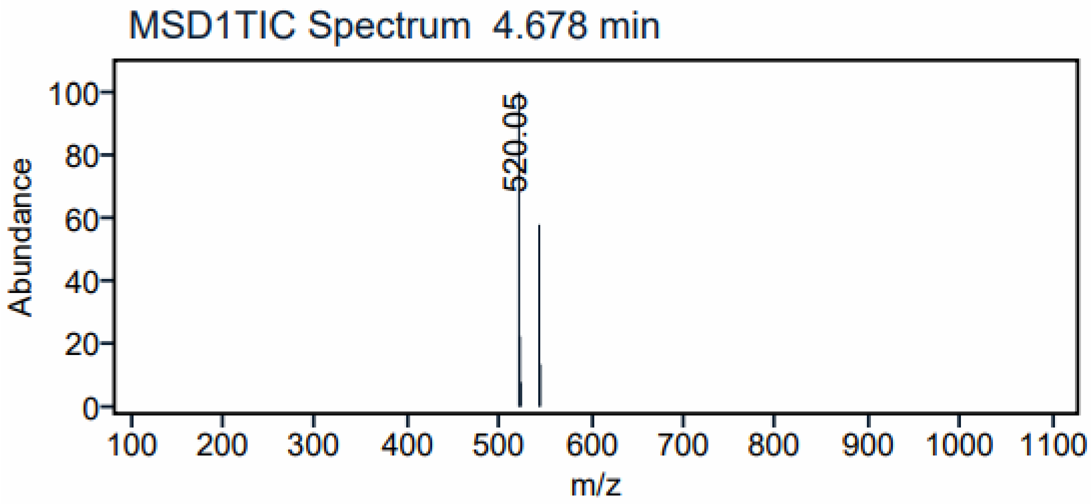
MSD1TIC Spectrum 4.678 min
Abundance
520.05
m/z

^{1}H-, ^{13}C-, ^{19}F-NMR-, HRMS- and LC-ESI-MS-spectrum of 6-[4-[2-(2-methylimidazo[1,2-b]pyridazin-6-yl)-4-oxo-pyrido[1,2-a]pyrimidin-7-yl]piperazin-1-yl]-6-oxo-*N*-[2-[2,3,5,6-tetrafluoro-4-(2,3,4,5,6-pentafluorophenyl)phenyl]sulfanylethyl]hexanamide **24 b**

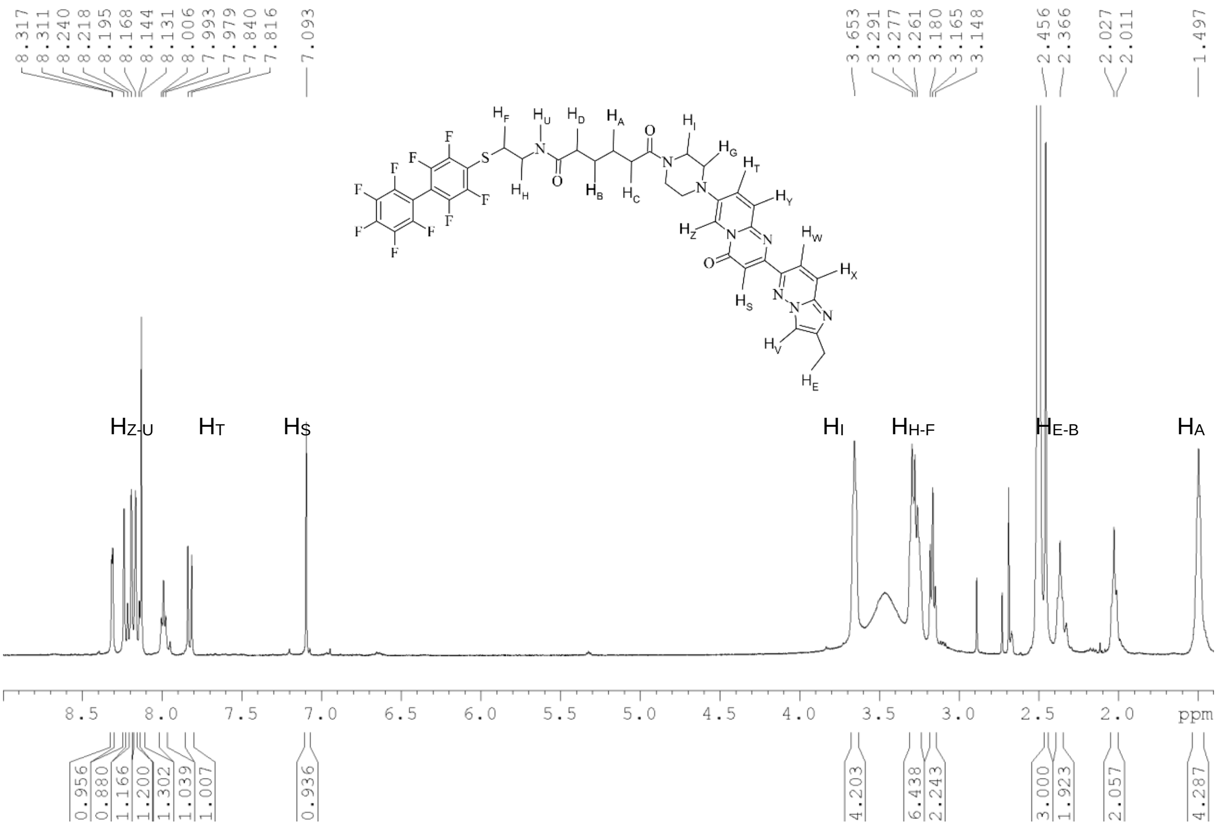

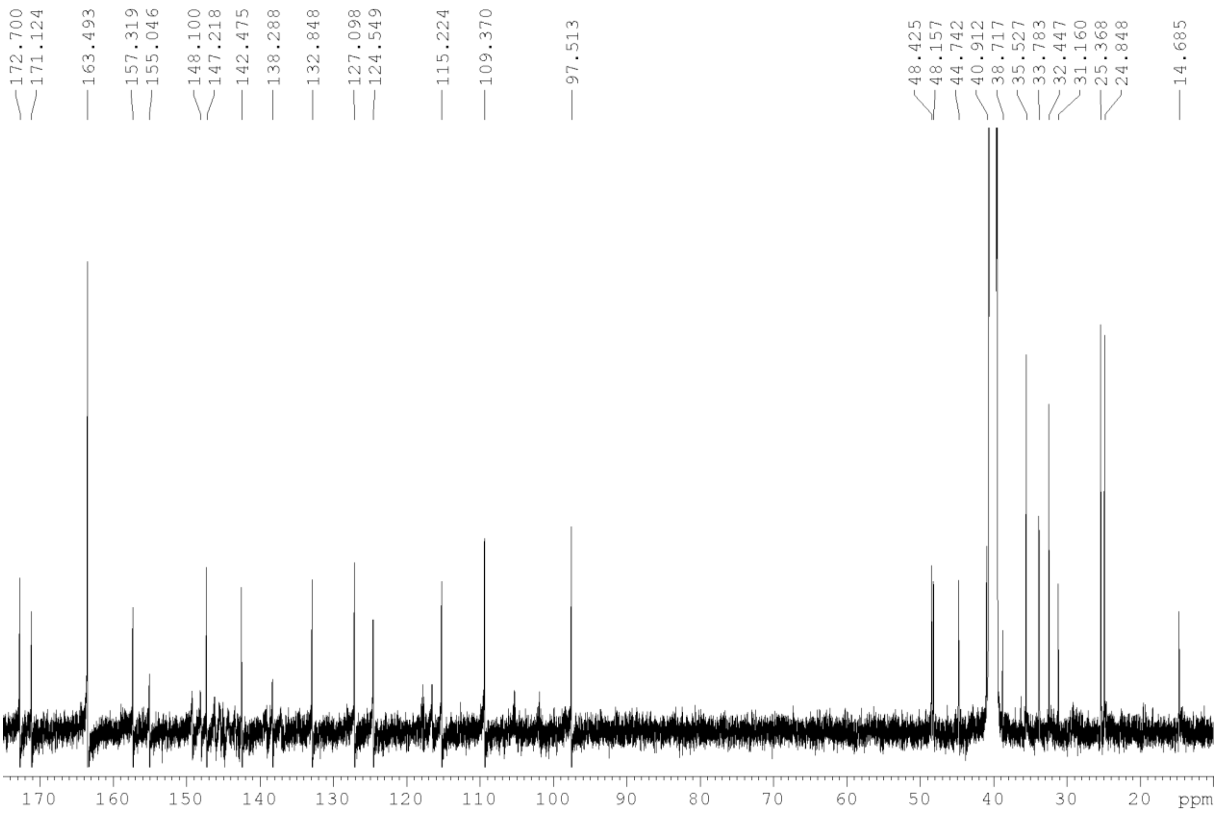

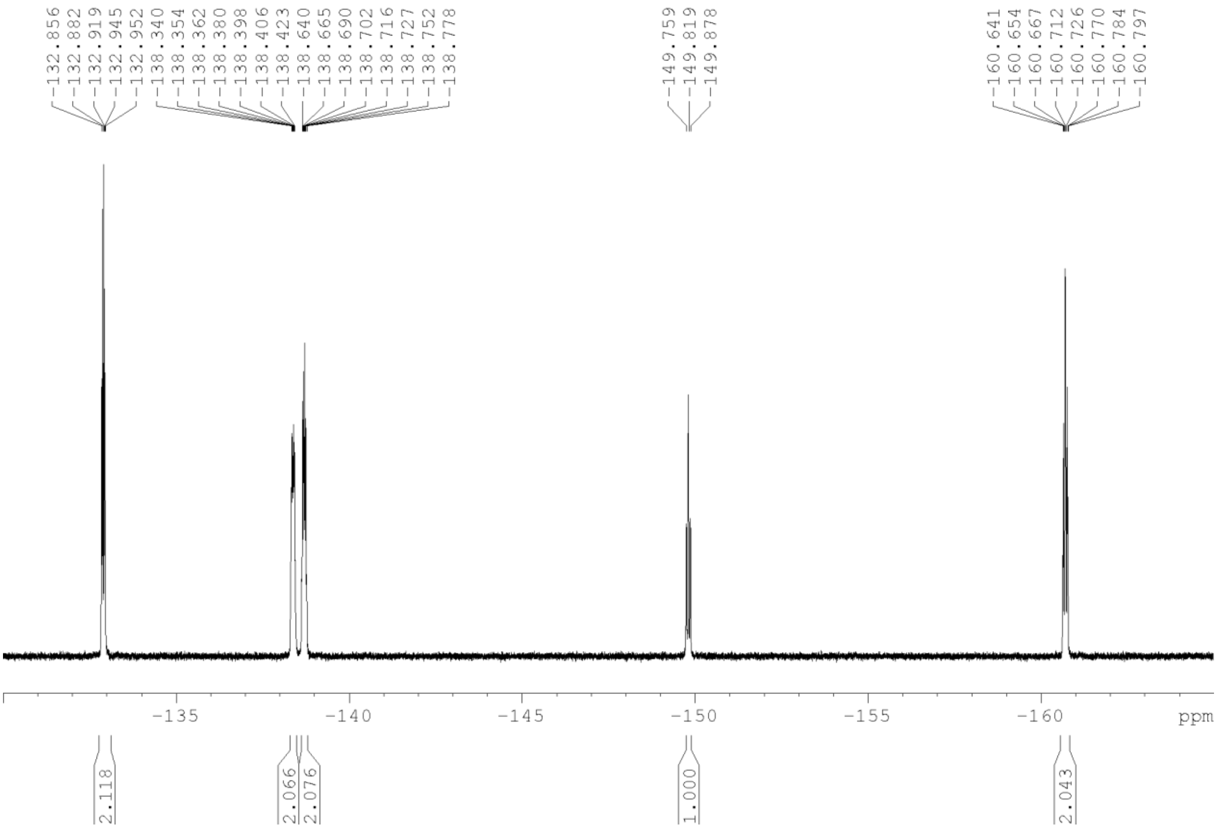

-132.856
-132.882
-132.919
-132.945
-132.952
-138.340
-138.354
-138.362
-138.380
-138.398
-138.406
-138.423
-138.640
-138.665
-138.690
-138.702
-138.716
-138.727
-138.752
-138.778
-149.759
-149.819
-149.878
-160.641
-160.654
-160.667
-160.712
-160.726
-160.770
-160.784
-160.797
-135
-140
-145
-150
-155
-160
ppm
2.118
2.066
2.076
1.000
2.043

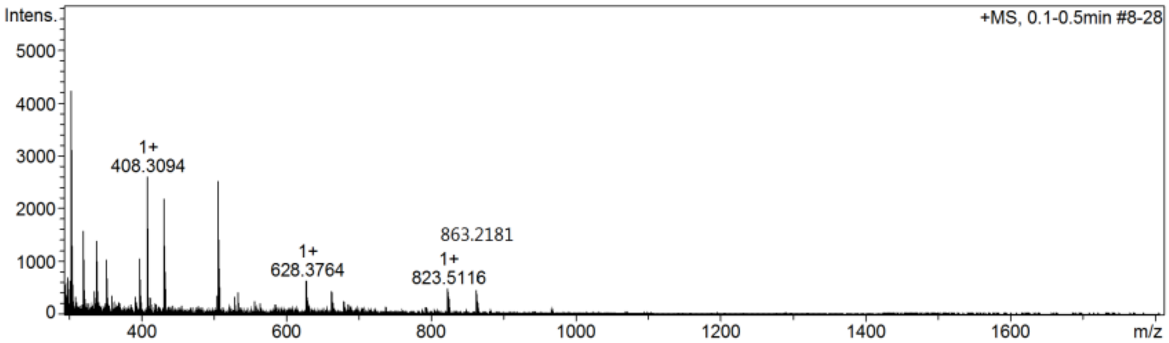

Intens.
+MS, 0.1-0.5min #8-28
1+
408.3094
1+
628.3764
863.2181
1+
823.5116
m/z

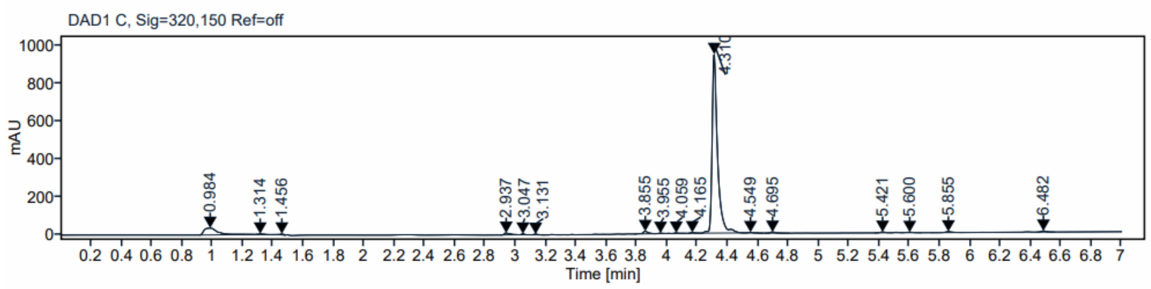

DAD1 C, Sig=320,150 Ref=off
mAU
Time [min]
0.984
1.314
1.456
2.937
3.047
3.131
3.855
3.955
4.059
4.165
4.310
4.549
4.695
5.421
5.600
5.855
6.482

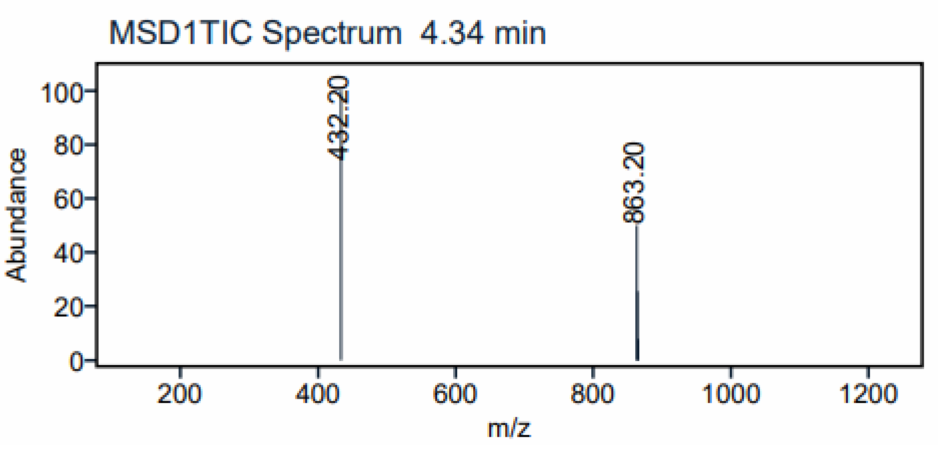

^{19}F-NMR-, HPLC- and HRMS-spectrum of *tert*-butyl 12-oxo-12-[2-[2,3,5,6-tetrafluoro-4-(2,3,4,5,6-pentafluorophenyl)phenyl]sulfanylethylamino]dodecanoate and ^{1}H-, ^{19}F-NMR-, HRMS- and LC-MS-spectrum of 12-Oxo-12-[2-[2,3,5,6-tetrafluoro-4-(2,3,4,5,6-pentafluoro-phenyl)phenyl]sulfanylethylamino]dodecanic acid **23 c**

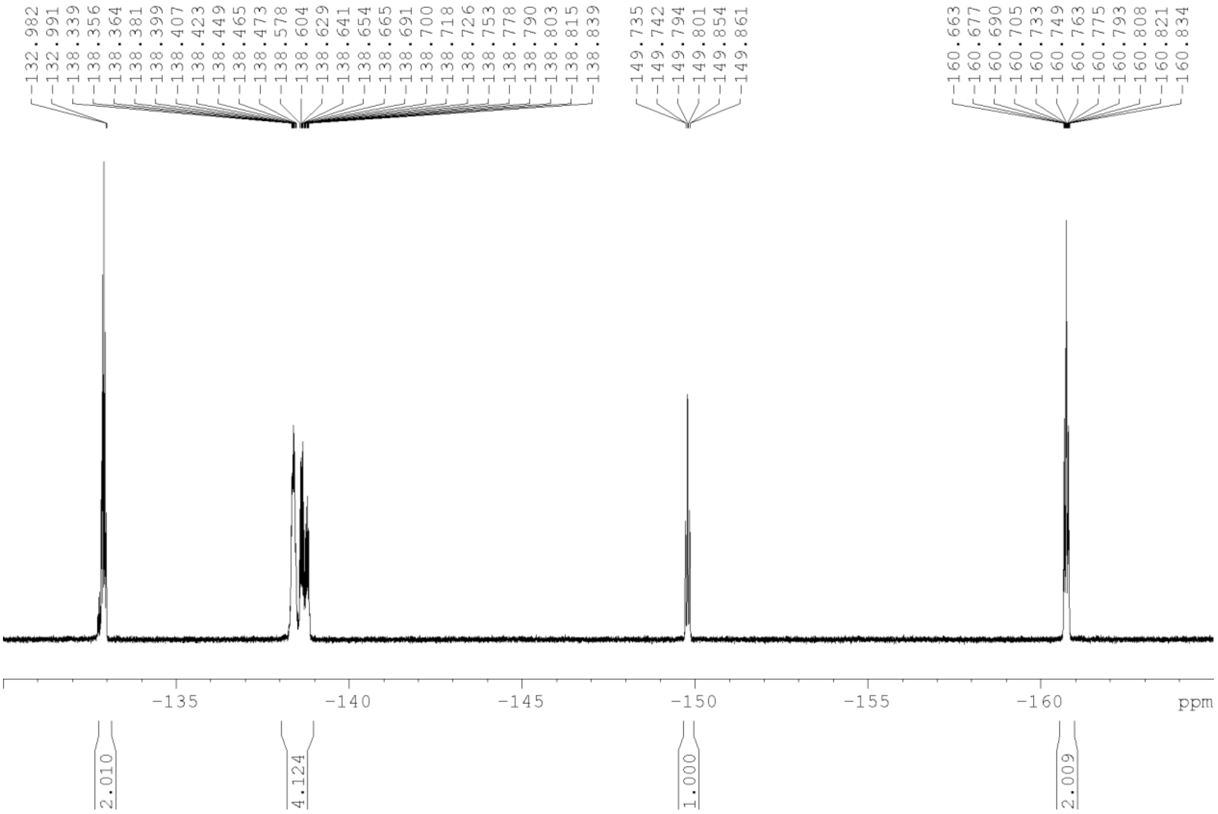

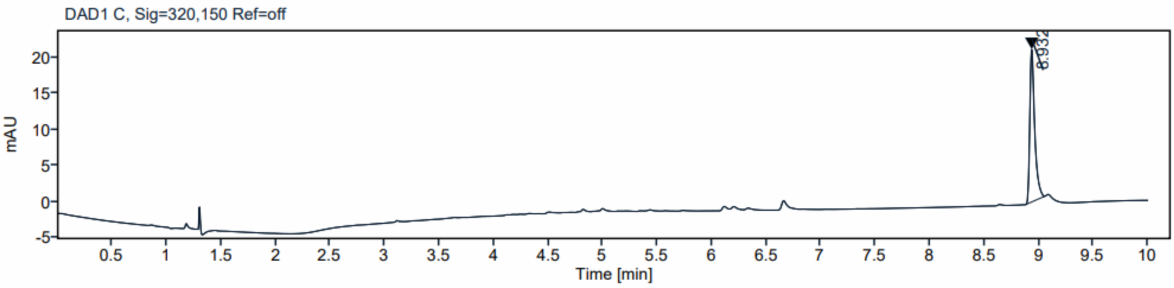

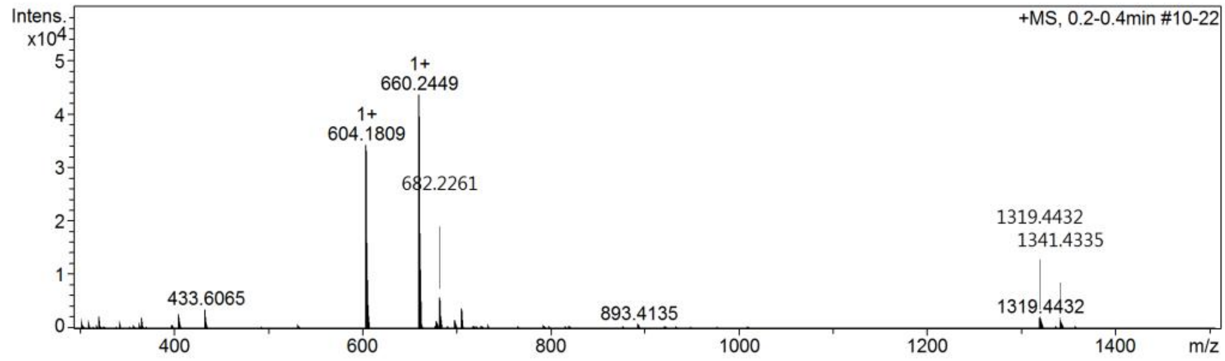
Intens.
x10^4
+MS, 0.2-0.4min #10-22
1+
660.2449
1+
604.1809
682.2261
1319.4432
1341.4335
433.6065
893.4135
1319.4432
400
600
800
1000
1200
1400
m/z

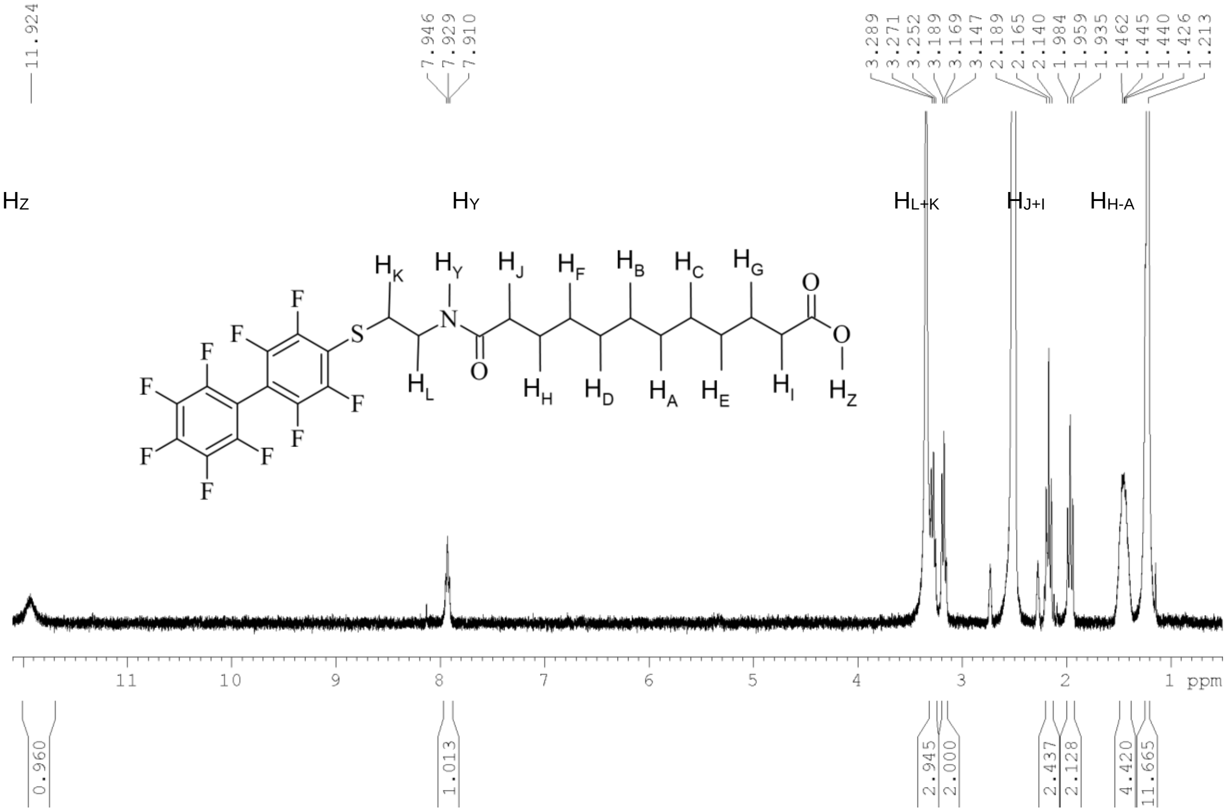
11.924
7.946
7.929
7.910
3.289
3.271
3.252
3.189
3.169
3.147
2.189
2.165
2.140
1.984
1.959
1.935
1.462
1.445
1.440
1.426
1.213
HZ
HY
HL+K
HJ+I
HH-A
ppm
0.960
1.013
2.945
2.000
2.437
2.128
4.420
11.665

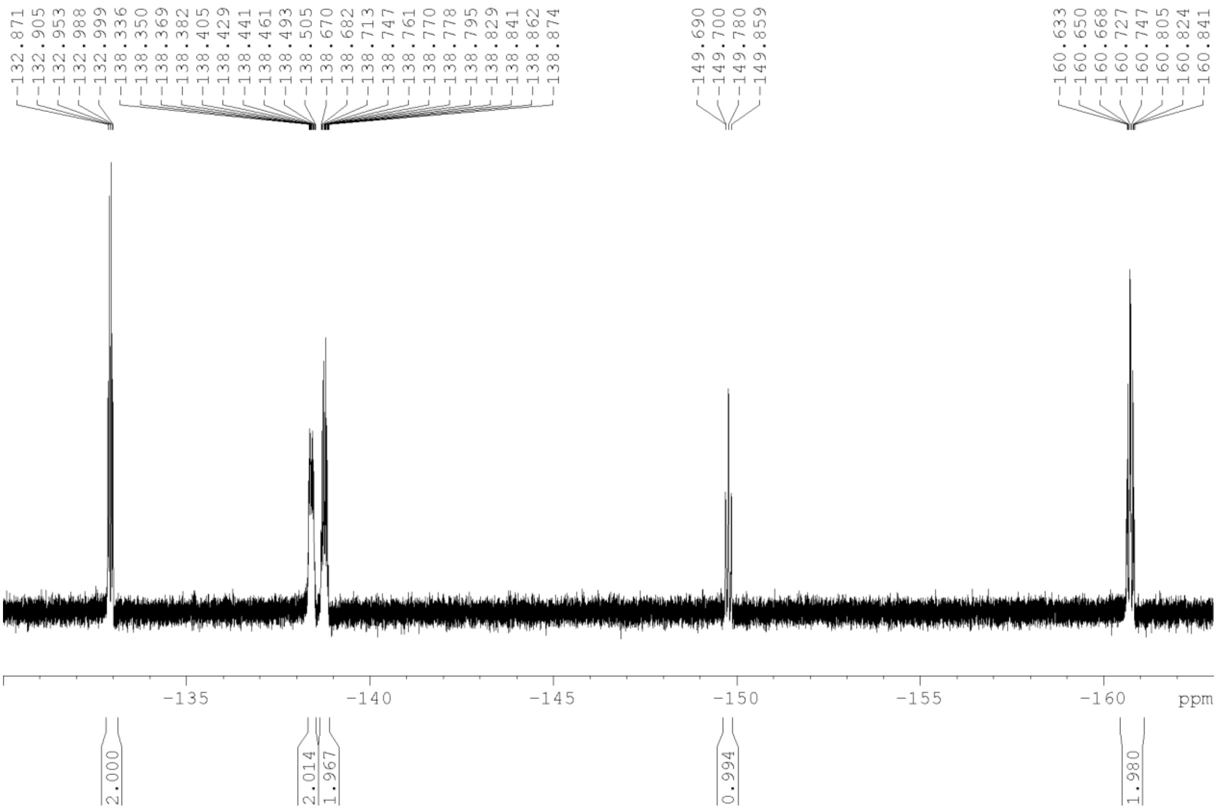
-132.871
-132.905
-132.953
-132.988
-132.999
-138.336
-138.350
-138.369
-138.382
-138.405
-138.429
-138.441
-138.461
-138.493
-138.505
-138.670
-138.682
-138.713
-138.747
-138.761
-138.770
-138.778
-138.795
-138.829
-138.841
-138.862
-138.874
-149.690
-149.700
-149.780
-149.859
-160.633
-160.650
-160.668
-160.727
-160.747
-160.805
-160.824
-160.841
-135
-140
-145
-150
-155
-160
ppm
2.000
2.014
1.967
0.994
1.980

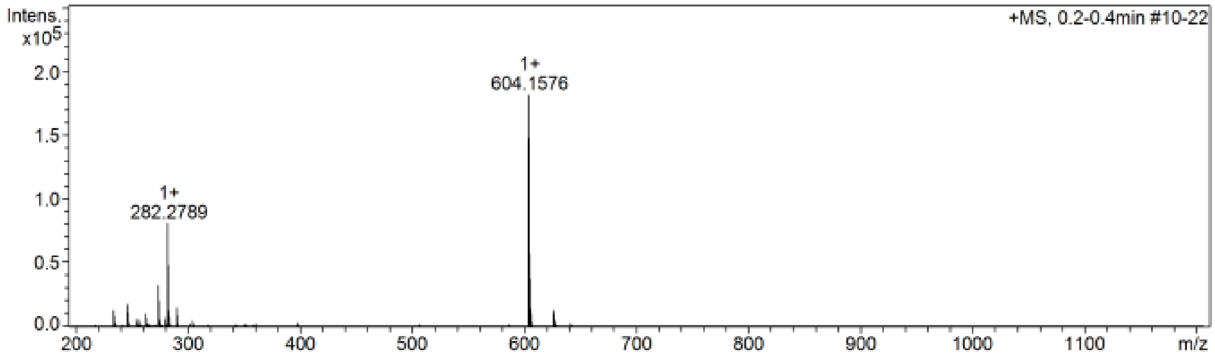
Intens.
x10^5
+MS, 0.2-0.4min #10-22
1+
604.1576
1+
282.2789
m/z

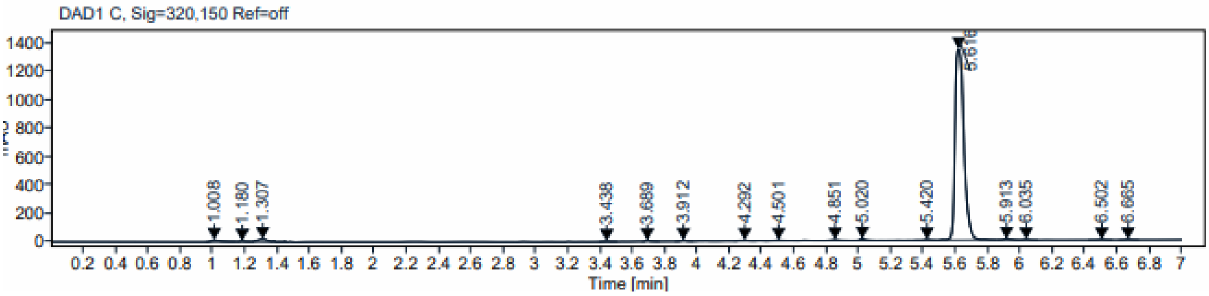
DAD1 C, Sig=320,150 Ref=off
1.008
1.180
1.307
3.438
3.689
3.912
4.292
4.501
4.851
5.020
5.420
5.616
5.913
6.035
6.502
6.665
Time [min]

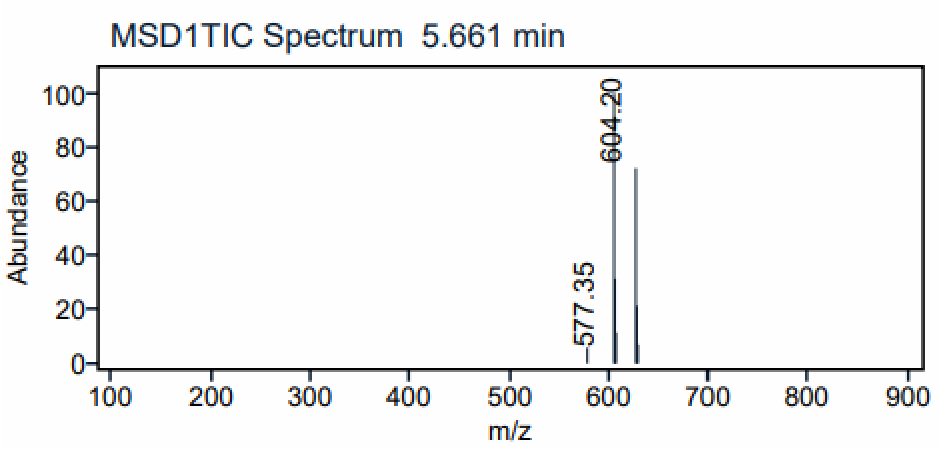

^{1}H-, ^{13}C-, ^{19}F-NMR- and LC-ESI-MS-spectrum of 12-[4-[2-(2-methylimidazo[1,2-b]pyridazin-6-yl)-4-oxo-pyrido[1,2-a]pyrimidin-7-yl]piperazin-1-yl]-12-oxo-*N*-[2-[2,3,5,6-tetrafluoro-4-(2,3,4,5,6-pentafluorophenyl)phenyl]sulfanylethyl]dodecanamide **24 c**

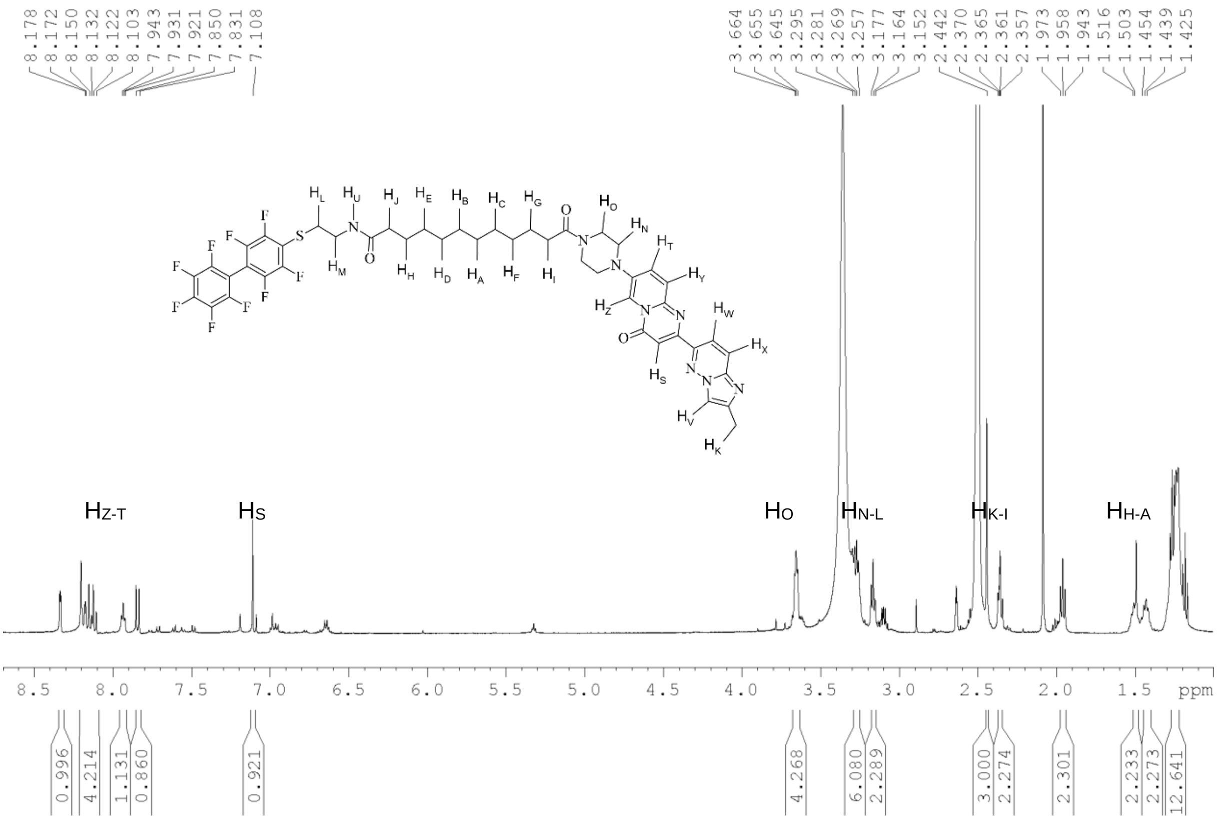

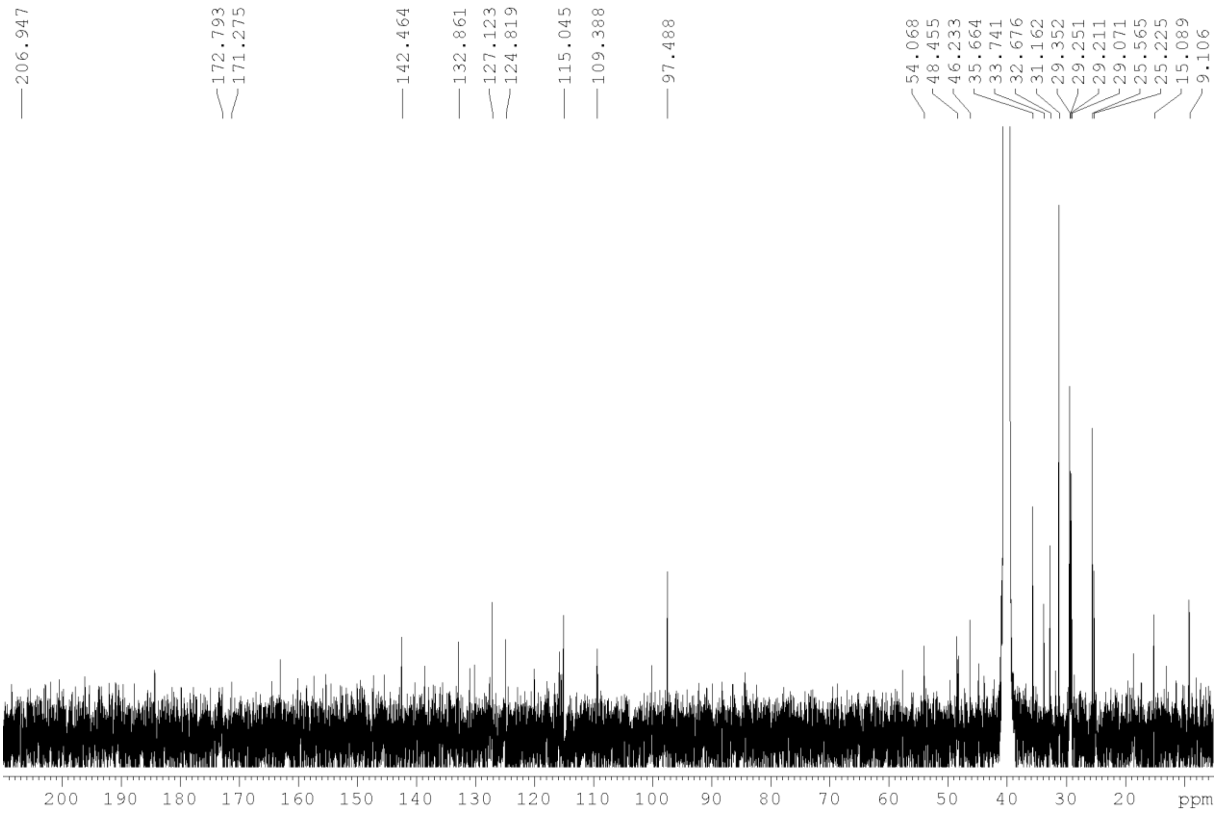
206.947
172.793
171.275
142.464
132.861
127.123
124.819
115.045
109.388
97.488
54.068
48.455
46.233
35.664
33.741
32.676
31.162
29.352
29.251
29.211
29.071
25.565
25.225
15.089
9.106
200 190 180 170 160 150 140 130 120 110 100 90 80 70 60 50 40 30 20 ppm

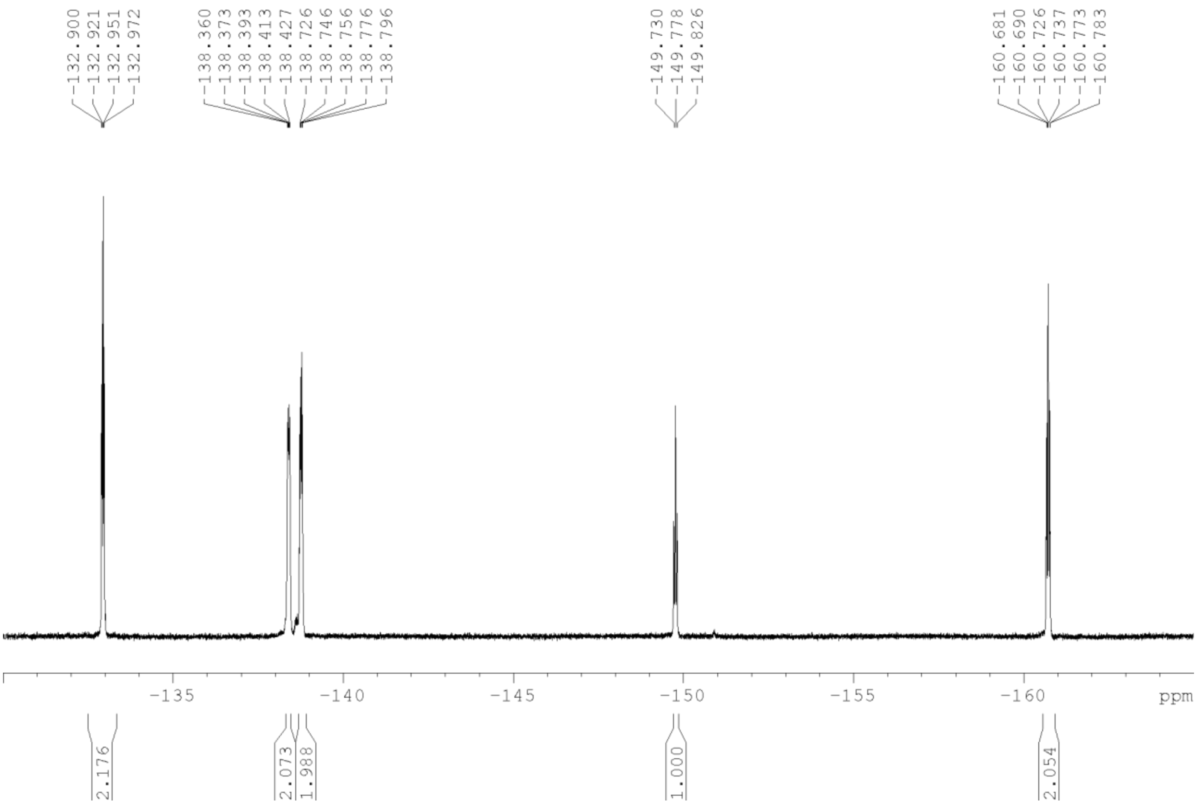
-132.900
-132.921
-132.951
-132.972
-138.360
-138.373
-138.393
-138.413
-138.427
-138.726
-138.746
-138.756
-138.776
-138.796
-149.730
-149.778
-149.826
-160.681
-160.690
-160.726
-160.737
-160.773
-160.783
-135 -140 -145 -150 -155 -160 ppm
2.176
2.073
1.988
1.000
2.054

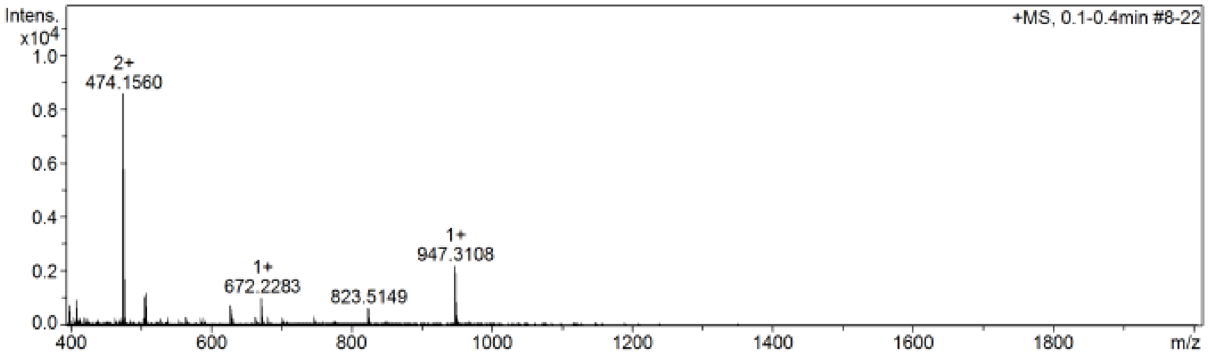

Intens.
x10^4
+MS, 0.1-0.4min #8-22
2+
474.1560
1+
672.2283
823.5149
1+
947.3108
m/z

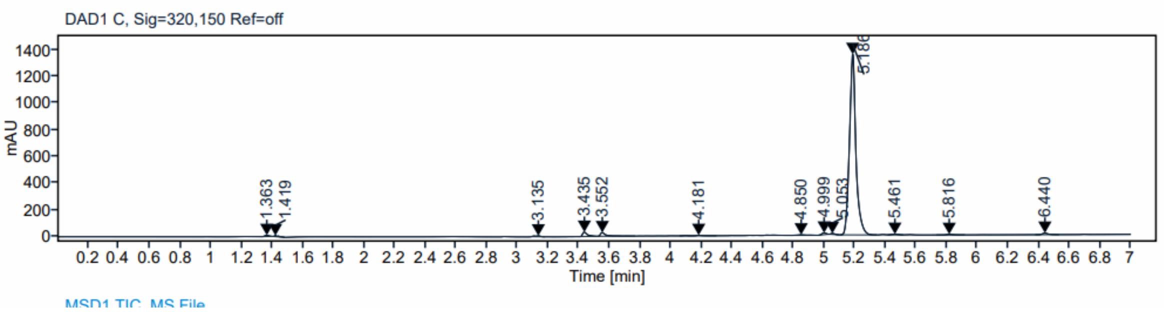

DAD1 C, Sig=320,150 Ref=off
mAU
Time [min]
5.186
1.363
1.419
3.135
3.435
3.552
4.181
4.850
4.999
5.053
5.461
5.816
6.440
MSD1 TIC, MS File

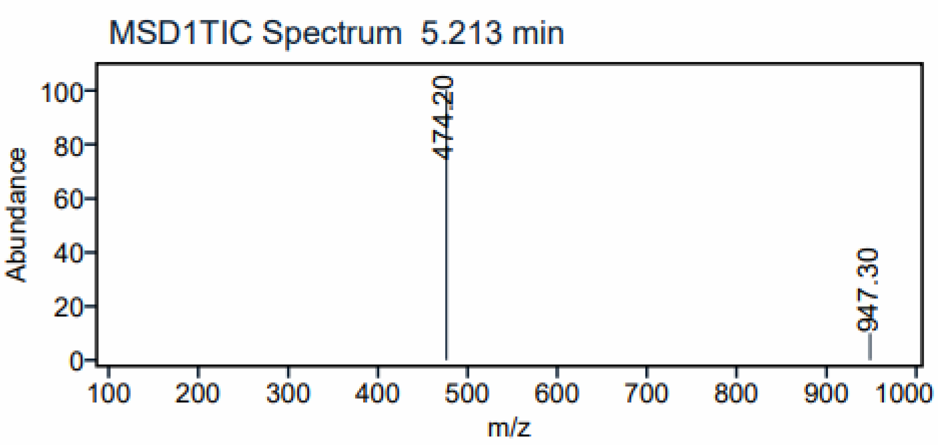

MSD1TIC Spectrum 5.213 min
Abundance
474.20
947.30
m/z
